# Supplementary material for: Inkube: an all-in-one solution for neuron culturing, electrophysiology, and fluidic exchange
Source: Lab Chip. 2026 Mar 2;26(6):2074–89. doi: 10.1039/d5lc00971e (PMC12951282; doi:10.1039/d5lc00971e)
Supplement: LC-026-D5LC00971E-s001 [file LC-026-D5LC00971E-s001.pdf]

Supplementary Information (SI) for Lab on a Chip.
This journal is © The Royal Society of Chemistry 2026

Inkube: An all-in-one solution for neuron culturing, electrophysiology, and fluidic exchange

Benedikt Maurer[†, a], Selina Fassbind[a], Tobias Ruff[a], Jens Duru[a], Giusy Spacone[a], Theo Rodde[a], János Vörös[a], and Stephan J. Ihle[†, a, b, c, ‡]

Assembly Instructions

[a]Laboratory of Biosensors and Bioelectronics, Institute for Biomedical Engineering, University and ETH Zurich, Gloriastrasse 37/39, Zurich, 8092, Switzerland
[b]Department of Neurobiology, University of Chicago, 951 E 58th St, Chicago, 60637, IL, USA
[c]Department of Physics, University of Chicago, 929 E 57th St, Chicago, 60637, IL, USA
[†]These authors contributed equally to this work
[‡]Corresponding author: ihles@uchicago.edu

Contents

A Hardware description (duplicate of main section) 2
A.1 Incubation layer . . . 2
A.2 Ventilation layer . . . 4
A.3 Electronics layer . . . 4
A.4 Fluidics layer . . . 4
A.5 Medium level measurement . . . 5

B Parts list 5

C Assembly overview 8

D PCBs 20

E Housing 26

F MEA Temperature Control 29
F.1 Peltier mounting . . . 29
F.2 RTD assembly . . . 29
F.3 Fan for heat sink and Peltier driver cooling . . . 31

G Electrophysiology 32
G.1 Electrophysiology board . . . 32

H Fluidics 33
H.1 Pump . . . 33
H.2 Multiplexing scheme . . . 34
H.3 Valve multiplexer . . . 35
H.4 Driver board and status LEDs . . . 36
H.5 Liquid reservoir . . . 37
H.6 Tubing and connection . . . 37

I Volume measurement 37
I.1 inkulevel . . . 37

J Environment control 39
J.1 Inkusense board 39
J.2 Reservoir resistive heater 39
J.3 CO_2 valve assembly 40
J.4 Humidity heater 41
J.5 Fan for stable control 41

K Equipment and unspecified components 41

L Installation and operation 42
L.1 SoC 42
L.2 PC connection 42
L.2.1 Ethernet 42
L.2.2 USB 42
L.3 System startup 42
L.4 Inkulevel 43

A Hardware description (duplicate of main section)

inkube consists of an electrophysiology setup, temperature controllers, a humidity controller, a CO_2 controller, a perfusion system, and a volumetric sensor to measure the volume of the culture medium. In total, inkube can measure and control up to 4 MEAs in parallel. The block diagram of the system is presented in Fig. A1A. The system is completely open-source. The assembly instructions as well as the parts-list can be found in Assembly Instructions A.5 of the supplementary information (SI). Pictures are shown that help assemble inkube (Fig. A2 - Fig. A40). The total cost of the system is approximately $12,000 USD, of which approximately $4,000 USD are required for the perfusion system (see cost breakdown in Table A1 in Assembly Instructions). Due to the system's modularity, the end user does not have to assemble the whole system but can instead only rebuild the required subparts to minimize the cost of inkube even further.

inkube uses the Arty Z7 (Digilent, Pullman, WA, USA) development board, which contains a Zynq-7000 SoC (XC7Z020-1CLG400C, Xilinx, San Jose, CA). It is communicating with a GNU/Linux personal computer (PC) via both an Ethernet connection and a USB connection. The Ethernet connection is used mostly to send data from inkube to the PC, while the PC predominantly sends commands to inkube via the USB connection. The Arty Z7 has been programmed in VHDL and C (C11), while the software on the PC side has been written in Python 3 as well as Cython 3. The software layout is presented in Fig. S1. inkube comes with a graphical user interface, which can plot neuronal activity in real time Fig. S4, the status of the system Fig. S5, environment parameters Fig. S6, raster plots Fig. S7, and spike shapes Fig. S8. Python was chosen due to its widespread use in scientific environments as well as its relative ease of use.

All components of inkube fit into a 3D-printed cube. The components are subdivided into 4 layers, where each layer has a unique function. We termed these layers (top to bottom): incubation, ventilation, electronics, and fluidics layer. An overview of the assembled 3D-printed inkube is given in Fig. A1B. The incubation layer contains the 4 MEAs, the sensors, and the electrophysiology components. The ventilation layer is cooling the electronics and is required for temperature regulation. The electronics layer houses most of the electronics. Finally, the components of the perfusion system are located in the fluidics layer, which is the largest and bottom most layer. In the following, each layer is discussed in more detail.

A.1 Incubation layer

The incubation layer is the top most layer and houses up to 4 MEAs, sensors, and the electrophysiology setup. The performance of which are described in Section 3.1.4. A picture of the incubation layer is given in Fig. A1C. The layer can be closed with a 3D-printed lid (see Fig. A37), which has been electrically shielded using aluminum tape to reduce electromagnetic interference on the electrophysiology setup.

inkube has multiple sensors, which are all placed in the incubation layer. These sensors are **1)** a temperature sensor for the air reservoir inside of the incubation layer **2)** a temperature sensor for each of the 4 MEAs, which measures the temperature of the culture medium in its designated MEA **3)** a humidity sensor, **4)** a CO_2 sensor of the air reservoir **5)**, and a surface reflection based height sensor we termed inkulevel, which measures the volume of the culture medium of each of the 4 MEAs. inkulevel is shown in Fig. A1D and is discussed further in Section 2.1.5.

The temperature and humidity of the air reservoir is measured with a SHTC3 sensor (Sensirion, Stäfa, Switzerland), while the CO_2 concentration is measured using a STC31 sensor (Sensirion, Stäfa, Switzerland). In Section 3.1.1, we present the capabilities of the temperature and humidity control. The SHTC3 cannot be used to measure the temperature of the medium reservoirs, as it communicates via digital data protocols which would interfere with the electrophysiology setup. Instead, an analog resistance temperature detector (RTD, PT1000) is being used here in a 4-wire configuration with an impedance measurement IC (MAX31865, Analog Devices, Inc., Wilmington, MA, USA) on the base board. The RTD is wrapped in biocompatible polytetrafluoroethylene (PTFE) tubing to make the sensor

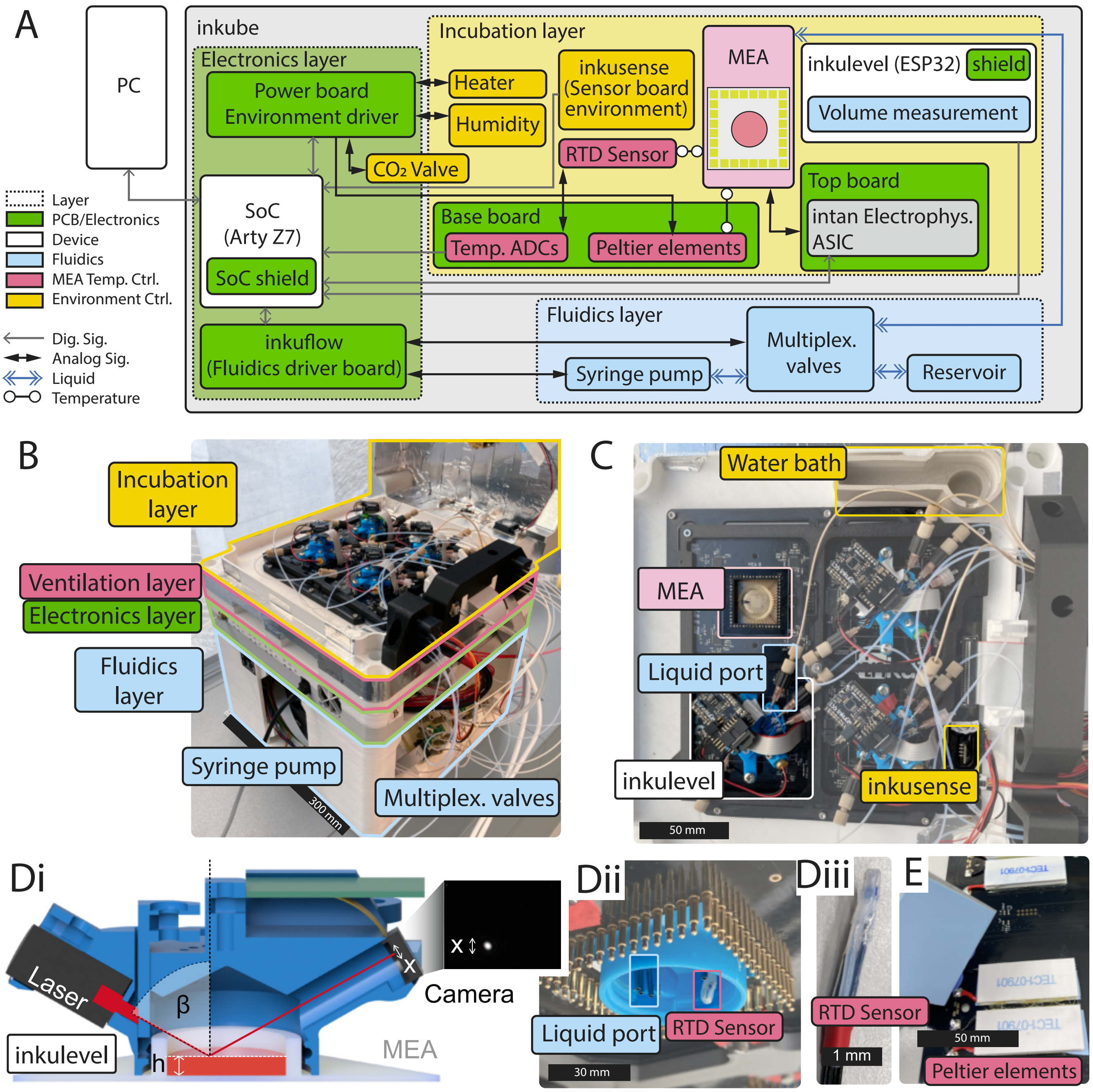

Figure A1 Block diagram and system overview. (A) The block diagram of inkube. The different layers of inkube are color-coded. **(B)** Picture of a 3D-printed inkube with an open lid (top-right) showing the different layers. **(C)** Zoom in of the incubation chamber, with 3 mounted inkulevels and one open MEA. Each of the inkulevels is connected to 4 liquid ports. **(D)** inkulevel can be used to measure the medium height in the well. It is a proxy for the overall culture medium volume. The cross section (i) shows the working principle: a laser is reflected by the liquid surface and projected onto a camera. The change of medium height is then extracted from the white dot in the image shown on the right. (ii) shows inkulevel from below without its top board. In (iii), the RTD sensor in its PTFE tube casing is shown. **E** Bottom view of two Peltier elements (white) below the base board are shown, with removed heat sink (left).

water tight. The RTD is mounted inside of inkulevel (see Fig. A1Dii). A disassembled RTD is shown in Fig. A1Diii, while the assembly instructions are given in Fig. A25. In Section 3.1.3 we discuss the dynamics and performance of this assembly.

inkube has multiple actuators to control temperature, CO_2 concentration, and humidity. Each MEA is located on top of 2 thermoelectric Peltier devices (see Fig. A1E), which can control the temperature of the MEAs independent of each other. This way, MEAs can be both heated and cooled down with respect to the reservoir temperature. The thermoelectric devices are mounted from below on the base board PCB. The reservoir temperature is controlled through resistive elements mounted inside of the lid (see Fig. A37B). In addition, the lid houses fans to circulate airflow

inside the reservoir (see Fig. A40 for assembly instructions). The humidity can be controlled through a waterbath, which is heated with a 3D printer cartridge heater (see Fig. A39). How accurately the actuators and sensors can control the reservoir parameters is described in Section 3.1.1.

The incubation layer also houses the electrophysiology system of inkube. In order to record activity from 4 MEAs with each having 59 electrodes and 1 reference electrode, a setup compatible for a total of 240 electrodes is required. In inkube this is achieved by utilizing 16 Intan RHS2116 ICs (Intan Technologies, Los Angeles CA, USA), as Intan ICs are established for recording neuronal activity from *in vitro* cultures[3]. The ICs communicate through an SPI bus with the SoC, where the clock and chip select signals are shared between all 16 ICs. The clock frequency of the bus is 12.5 MHz, which enables sampling each electrode at a frequency of 17,361 Hz. For stimulating neuronal cultures, supply voltages of +7 V and -7 V are provided from the electronics layer. The recording and logic chip supply of 3.3 V is generated from a 5 V supply with an additional low-dropout regulator (LDO) in order to decrease the noise affecting the recording electronics. The RHS2116 ICs as well as the LDO are mounted on the PCB termed top board. When inkube is running, the MEAs are sandwiched between the top board and the base board, which houses the thermoelectric actuators.

A.2 Ventilation layer

The ventilation layer allows for air flow through inkube. It is required to heat and cool the other layers. Air, sucked in through 4 slits on two opposite sides of inkube, flows past the heat sinks of the Peltier elements (see Fig. A24A for assembly). The flow is powered by a fan at the bottom of inkube (see Fig. A26, which blows the air out through the bottom. To enable undisturbed air flow, inkube is mounted on small rubber feet (see GX5_121_feet in table A2, RND 455-00523, Distrelec Group AG, Nänikon, Switzerland).

A.3 Electronics layer

The electronics layer houses most of the electronics of inkube including the SoC that controls the whole system, which is part of the Arty Z7 development board. The onboard ethernet and USB ports are used for communication between PC and inkube. The peripheries of inkube are also connected to the development board. The electronics layer has an additional fan for cooling, which is shown in Fig. A27. On the wall of the electronics layers LEDs are mounted, which visualize the state of the valves of the perfusion system (see Section 2.1.4).

The CO_2 concentration of the air inside the reservoir is controlled through a valve (PVQ13-6M-06-M5-A, SMC Pneumatics, Tokyo, Japan) placed inside the electronics layer (see Section 3.1.1 for performance). CO_2 is provided through an external high pressure CO_2 source at approximately 1.5 bar. The assembly as well as tubing for the CO_2 is shown in Fig. A38.

There are three PCBs in this layer: **1)** the power board (see Fig. A14), **2)** the perfusion board (see Fig. A18), and **3)** the Arty Z7 shield (see Fig. A13). The power board contains all the electronic components that require high amounts of power, except for the perfusion system. These are the operational amplifiers (PA75CC, Apex Microtechnology, Tucson AZ, USA), that drive the Peltier elements for MEA heating and cooling as well as the reservoir and humidity heater. It also houses the driver of the CO_2 valve and provides stable power sources to the other PCBs. The voltage levels provided are -7, 3.3, 5, and 7 V. The perfusion board contains the drivers for the syringe pump and the valves required for the liquid multiplexing in the fluidics layer. The syringe pump is controlled by a stepper motor driver (A4988, Pololu Robotics and Electronics, Las Vegas NV, USA), while the valves are controlled by switchable current sources (STP04CM05, STMicroelectronics, Plan-les-Ouates, Switzerland). All of these components are controlled by the SoC via a shift register. Finally, the Arty Z7 shield acts as an interface between the SoC and the top board. Its main task is to transform the CMOS logic signals from the SoC into the low-voltage differential signals that the top board requires for the electrophysiology chips. In addition, it acts as an interface between the SoC and the sensor board, the inkulevels, and the 4 medium-temperature sensors.

A.4 Fluidics layer

The fluidics layer is the bottom most layer of inkube and contains a dry bath to chill a 50 mL centrifuge tube through two additional Peltier elements (see Fig. A34). The dry bath is not actively controlled, and once stable, its temperature fluctuates between 4.5 °C and 6.5 °C (see Fig. S19). It further contains a custom perfusion system consisting of a 3D-printed linear perfusion pump designed specifically for inkube and 4 liquid multiplexers. The syringe pump controls up to 4 syringes (1 mL) in parallel and is built on top of a motorized linear stage (DG281-100, RobotDigg Shanghai, Shanghai, China). The assembly of the pump is given in Fig. A29. The perfusion system is capable of delivering 3 different media or drugs to each of the 4 MEAs independently. Simultaneously, it can aspirate culture medium from the MEAs. Therefore, 4 different liquids can be pumped in total.

The liquid path of the perfusion system is shown in Fig. A30. Resin printed multiplexers are used to distribute the 4 different liquids to the relevant MEA. They are printed with biocompatible resin (POWERRESINS SG CLEAR RESIN, 3BFAB BV, Amsterdam, Netherlands). Each of the 4 syringes is connected to a liquid multiplexer. The liquid multiplexer determines which fluidic link is selected. It can either connect the syringe to one of the 4 MEAs or to the corresponding liquid reservoir to refill the syringe. The switching is done through 5 normally-closed 2-way valves (MVL-22-NC-08-14P-PEEK-SIL or for negative pressure MVL-22-NC-08-03P-PEEK-SIL, Memetis GmbH, Karlsruhe, Germany). Since all

4 syringes are controlled by the same syringe pump, one of the 5 paths of the multiplexer needs to be open for all 4 liquids at any given point. Otherwise, pressure will build up in the perfusion system. Hence, each liquid that is not supposed to be pumped into a culture must be connected to its corresponding reservoir. As there are 5 valves per multiplexer and 4 multiplexers, a total of 20 valves is required. For visual feedback, LEDs on the side of the electronics layer indicate which of the valves is currently open (see Fig. A33). Before an experiment, the tubes and multiplexers of all liquids are flushed for cleaning with first 1.4 % sodium hypochlorite in aqueous solution (7681-52-9, VWR International, Radnor, PA, USA) diluted with ultrapure water, followed by a flush with ultrapure water. Afterwards, the tubes and multiplexers were flushed a final time with the type of liquid to be pumped during the experiment, except for the path for liquid retrieval.

A.5 Medium level measurement

The medium level measurement system, named inkulevel, provides feedback about the liquid level in the measurement wells with help of a laser (PICO 70125658, Reichelt electronics GmbH, Sande, Germany). We discuss the performance of inkulevel in Section 3.1.2. A cross section of inkulevel is shown in Fig. A1Di. Its PCB is shown in Fig. A21. The height h of the medium defined in this figure is a proxy of the volume of the medium inside the well. While the absolute value is not exact as the surface tension of the medium with regard to the rim of the MEA may have inconsistent curvatures, relative changes can be tracked. The height can be calculated using a camera to capture the reflection point of a laser beam on the water surface. For that, the brightest pixel of the camera image could be used. However, sub-pixel resolution is possible by utilizing the entire shape of the measured reflection and deriving sub-pixel shifts.

To calculate the changes in signal for inkulevel, let x be the measured location of the reflection and β be the angle of incidence. For inkulevel, the angle of incidence is 60 °. Ignoring surface tension effects, we can calculate the changes in medium level height Δh as:

$$\Delta h = \Delta x \cdot \cos(\beta), \tag{1}$$

where Δx is the change of the measured reflection dot on the camera sensor. Using an angle of incidence of 60 °we get that changes of the medium height are half as big as the changes on the camera sensor. As a camera, the ESP32-CAM board (Ai-Thinker, Shenzhen, Guangdong, China) with the OV2640 camera sensor (OmniVision Technologies, Santa Clara, CA, USA) is used without a lens. This board is also programmed to perform the image analysis to extract the location of the laser beam (based on[2]), which it then sends to the SoC through a universal asynchronous receiver-transmitter (UART) connection. The laser and the ESP32-CAM board are mounted together with the liquid inlets and outlets on a 3D-printed cap, that can be placed on top of the MEA. The 3D-printed parts are made of Phrozen Aqua Resin and printed on a Sonic Mini 4K (Phrozen, Hsinchu, Taiwan). The mounting steps for inkulevel are given in Fig. A36. A spring loaded screw is used to adjust the pinhole height for the laser in order to compensate for small variations caused by the 3D-printing. When an image is captured by the camera the location of the laser reflection is determined by integrating the pixel values in each row of the sensor. The maximal integral is considered as the center of the laser reflection if it is larger than a threshold (standard value set to 10×255) and the brightest pixel has a value larger than 200. For subpixel resolution, a weighted average is then taken of the 20 rows around the maximum value, multiplied by 10 and rounded to an integer. Therefore, a difference Δx of 4.5 µm on the sensor translates to 10 bit. The value is then transmitted to the SoC. For an invalid measurement the maximum (6000 as the image resolution is set to 800 by 600 pixels) is transmitted to the SoC.

B Parts list

Table A1 Component costs breakdown

**Category**	**Cost (USD)**
General (G)	1,004
Electrophysiology (P)	6,576
Environment control (E)	144
Fluidics (F)	3,936
Volume feedback (V)	83
MEA Temperature control (M)	333
**Grand Total**	**12,076**

Table A2 Parts list

File Name	Description	Linked assembly
FA5_001_dry_bath	dry bath for cooling	-
FA5_002_valve_mux	multiplexer with valves	-
FA5_003_pump	syringe pump with stepper motor	-
MA5_004_bot_fan	Fan for peltier cooling	-
FA5_005_inkuflow	Valve and pump driver	-
GA5_006_fpga	FPGA with shield	-
GA5_007_power	power management and drivers	-
EA5_008_co2	CO2 valve and connectors	-
FA5_009_led	LED status for fluidics valve state	-
GA5_010_electronics_fan	fan to cool electronics and drivers	-
GA5_011_base	bottom board with MEA holder and ...	-
PA5_012_phys	electrophysiology board	-
VA5_013_inkulevel	volume sensor	-
EA5_014_inkusense	temperature, hum, co2 sensor	-
MA5_015_mea_sensor	RTD for MEA temperature	-
FA5_016_tube_fitting	fitting to seal the incubation ch...	-
EA5_017_humidity	water bath with heater for humidi...	-
EA5_018_lid_fan	fan in the lid for better control	-
EA5_019_res_heater	resistive reservoir heater	-
GD1_020_inkube_pwr_v4	System power and environment drivers	GA5_007_power
GD1_021_inkube_fpga_v4	FPGA shield	GA5_006_fpga
GD1_022_inkube_bot_v4	MEA base board with temperature c...	GA5_011_base
PD1_023_inkube_top_v4	MEA contacting and electrophysiology	PA5_012_phys
FD1_024_inkube_flw_v4	Valve and pump driver	FA5_005_inkuflow
FD1_025_inkube_led_v4	Valve multiplexer status LEDs	FA5_009_led
FD1_026_inkube_valve_v4	Valve multiplexer connector	FA5_002_valve_mux
VD1_027_inkube_lvl_v4	ESP32 shield	VA5_013_inkulevel
GD1_028_inkube_mea_v4	Holder for MEA	GA5_011_base
ED1_029_sensor_board	sensor board	EA5_014_inkusense
GA5_030_housing	housing	-
FD2_031_housing_0	fluidics layer	GA5_030_housing
GD2_032_housing_1	electronics layer	GA5_030_housing
GD2_033_housing_2	ventilation layer	GA5_030_housing
GD2_034_housing_3	mea layer	GA5_030_housing
FD2_035_housing_4A	side access with tube openings	GA5_030_housing
GD2_036_housing_4B	side access closed	GA5_030_housing
GD2_037_housing_5A	lid	GA5_030_housing
GD2_038_housing_5B1	lid with open top	GA5_030_housing
GD2_039_housing_5B2	acrylic glass for lid	GA5_030_housing
FA4_040_005_to_002	14-pin ribbon cable	-
FA4_041_005_to_009	20-pin ribbon cable	-
FA4_042_006_to_005	12-pin ribbon cable	-
GA4_043_006_to_007	8-pin ribbon cable	-
GA4_044_006_to_011	12-pin ribbon cable	-
GX4_045_007_to_006	Molex cable (4-pin) Microfit 3.0	-
PX4_046_007_to_012	Molex cable (4-pin) Microfit 3.0	-
FX4_047_007_to_005	Molex cable (4-pin) Microfit 3.0	-
GA4_048_007_to_011	14-pin Power cable	-
GX4_049	Molex Microfit 3.0 Crimp	GA4_048_007_to_011
GX4_050	Molex Microfit 3.0 14CKT receptable	GA4_048_007_to_011
PA4_051_006_to_012	68-pin ribbon cable	-
VA4_052_011_to_013	10-pin ribbon cable	-
PX4_053	Ribbon cable (68-pin, 0.025')	PA4_051_006_to_012
PX4_054	68-pin connector	PA4_051_006_to_012
GX4_055	8-pin connector	GA4_043_006_to_007
VX4_056	10-pin connector	VA4_052_011_to_013
FX4_057	14-pin connector	FA4_040_005_to_002
FX4_058	12-pin connector	FA4_042_006_to_005

GX4_059	12-pin connector	GA4_044_006_to_011
FX4_060	20-pin connector	FA4_041_005_to_009
GX4_061	ribbon cable 0.05’, 30m	-
GX5_062	cable for Molex	-
GX4_063_006_to_PC	USB-A to USB-A, 3.0, 1.8m	-
GX4_064_006_to_PC	Patch cable right angle	-
GX5_065_power_supply	Power supply 9V	-
GX3_066_soc	Zynq FPGA board	GA5_006_fpga
VX3_067_esp	ESP32-CAM AI Thinker Camera Module	VA5_013_inkulevel
FX3_068_motor_driver	pump driver AD4988	FA5_005_inkuflow
MX5_069_sink	Heat Sink	GA5_011_base
MX5_070_epoxy	Araldite Instant Epoxy	GA5_011_base
MX5_071_pad	Thermal gap pad	GA5_011_base
MX5_072_pt	PT1000 2.0x4.0 B	MA5_015_mea_sensor
MX6_073_seal	PTFE heat shrink tube	MA5_015_mea_sensor
EX5_074_valve	CO2 Valve	EA5_008_co2
EX6_075_tube	CO2 tubing, 50m	EA5_008_co2
EX5_076_con	CO2 wall connector	EA5_008_co2
FX5_077_valve	memetis microvalves	FA5_002_valve_mux
FX5_078_valve_ov	memetis microvalves negative pres...	FA5_002_valve_mux
EX5_079_hum_heater	Cartridge Heater Kit	EA5_017_humidity
MX5_080_bot_fan	Axial Fan peltiers	MA5_004_bot_fan
GX5_081_el_fan	Axial Fan electronics	GA5_010_electronics_fan
EX5_082_lid_fan	Axial Fan lid	EA5_018_lid_fan
FX5_083_sink_bath	heat sink for dry bath	FA5_001_dry_bath
FX5_084_pad_sticker	Thermal Gap Pad 0.13mm thickness	FA5_001_dry_bath
ED2_127_bath	bath for humidity	GA5_030_housing
PD2_085_spacer_r	spacer between base and e-phys. B...	PA5_012_phys
PD2_086_spacer_l	spacer between base and e-phys. B...	PA5_012_phys
PD2_087_spacer_r_flex	spacer between base and e-phys. B...	PA5_012_phys
PD2_088_spacer_l_flex	spacer between base and e-phys. B...	PA5_012_phys
PD2_089_spacer_bot	spacer between base and e-phys. B...	PA5_012_phys
PD2_090_spacer_bot_flex	spacer between base and e-phys. B...	PA5_012_phys
PX5_092_spring_contact	spring contact for MEA	PA5_012_phys
PD2_091_spacer_cover	spacer between base and e-phys. B...	PA5_012_phys
VD2_094_bending_helper	to bend syringe needles	VA5_013_inkulevel
VX5_095_clamp	Clamp Syringe	VA5_013_inkulevel
VX5_096_laser	Laser module 650 nm, 6-12 VDC	VA5_013_inkulevel
FX5_097_needle	Blunt needle Sterican® 18G / 1,2 ...	VA5_013_inkulevel
FX5_098_syringe	Syringe 1mL	FA5_003_pump
FD2_099_pump_sledge	Sledge for syringe pump	FA5_003_pump
FD2_100_pump_back	Back part syringe pump	FA5_003_pump
FD2_101_pump_front	Front part syringe pump	FA5_003_pump
FD2_102_pump_base	Base plate syringe pump	FA5_003_pump
MX5_103_peltier	TEC device	GA5_011_base
FX5_104_peltier	TEC device	FA5_001_dry_bath
FX5_105_luer	Luer Fingertight	-
FX5_106_luer_f	Luer female	-
FX5_107_luer_m	Luer male	-
FX6_108_tube	PTFE Liquid tubing ID 1mm, x1m	-
PX5_109_tape	Aluminum adhesive tape for shielding	GA5_030_housing
FD2_110_valve_splitter	multiplexer with valves print	FA5_002_valve_mux
FX5_111_motor	Mini T6 LeadScrew Linear Motion S...	FA5_003_pump
FD2_112_holder	syringe holder counter	FA5_003_pump
FX5_113_switch	safety switch, Micro Switch, DG, ...	FA5_003_pump
FX5_114_bearing	6 mm linear bearings	FA5_003_pump
FX5_115_shaft	6 mm precision shafts	FA5_003_pump
FD2_116_dry_bath_back	dry bath	FA5_001_dry_bath
FD2_117_dry_bath_cap	dry bath	FA5_001_dry_bath
EX5_118_adapter	CO2 tube adapter	EA5_008_co2
EX5_119_angle	CO2 tube angle	EA5_008_co2

GX5_120_feet	rubber feet	GA5_030_housing
VX5_121_spring	spring for inkulevel pinhole	VA5_013_inkulevel
VD2_122_inkulevel_body	main part for volume sensor	VA5_013_inkulevel
VD2_123_inkulevel_pinhole	height adjustable pinhole for laser	VA5_013_inkulevel
VD2_124_inkulevel_top	counter for pinhole	VA5_013_inkulevel
PX5_125_mea	MCS MEA 60MEA500/10iR-Ti -gr	-
EX6_126_seal_hum	wrapping for humidity heater	EA5_017_humidity
PD2_127_mounting_helper	to mount spring contacts to e-phy...	PX4_046_007_to_012
EX5_128_heater_sink	4 Ohm resistor for heating with h...	EA5_019_res_heater
EX5_129_heater_tht	4 Ohm resistor high power	EA5_019_res_heater
FD2_130_dry_bath_cover	dry bath cover to prevent condens...	FA5_001_dry_bath
FD2_131_tube_lid	cap for falcon tube with hole for...	FA5_001_dry_bath
GD2_132_lid_hook	holder for lid when open	GA5_030_housing
FD2_133_tube_fitting	fitting to seal the incubation ch...	FA5_016_tube_fitting
MX6_134_seal_small	PTFE heat shrink tube	MA5_015_mea_sensor

The partslist lists all components required to build the inkube hardware. The filename serves as a unique identifier. The first letter indicates the functionality, i.e. for which feature of inkube the parts are required. The encoding is as follows:

- G (**G**eneral), these are essential components that are always required
- P (Electro**p**hysiology), required to record and stimulate neuronal cultures
- E (**E**nvironment control), required for temperature, humidity, and CO_2 control in the reservoir
- F (**F**luidics), required to pump and retrieve liquid into and from the MEA
- V (**V**olume feedback), required to obtain feedback about the volume in the MEA
- M (**M**EA Temperature control), required to individually control the medium temperature inside the MEA

The second letter indicates whether this is an external component to be bought (X), a design file for a CAD or PCB that is provided in the supplementary data for this paper (D), or an assembly of components (A). The third digit describes the component category listed below:

1 PCB

2 CAD

3 Electronic Devices

4 Cable

5 Component

6 Tube

A more detailed list containing quantity, suppliers, and cost can be found in the Supplementary Data (01_Hardware/01_BOM_system). The design files of the PCBs can be found in the Supplementary Data at `01_Hardware/02_PCB` and the design files for the CAD `01_Hardware/03_CAD`. The parts lists for assembly of the PCBs can be found in the same folder as the PCB design `BOM_PCBs.ods`.

C Assembly overview

This section contains an overview of how the system is arranged with all components marked as assemblies. The first figure shows the CAD model in a side view. Next, the housing is depicted in top view from lowest layer (fluidics) to topmost layer (incubation) with annotations where to place other assemblies and cables connecting these. All parts are marked with the unique identifiers from the parts list. Finally, the same layers are shown with annotations for screws.

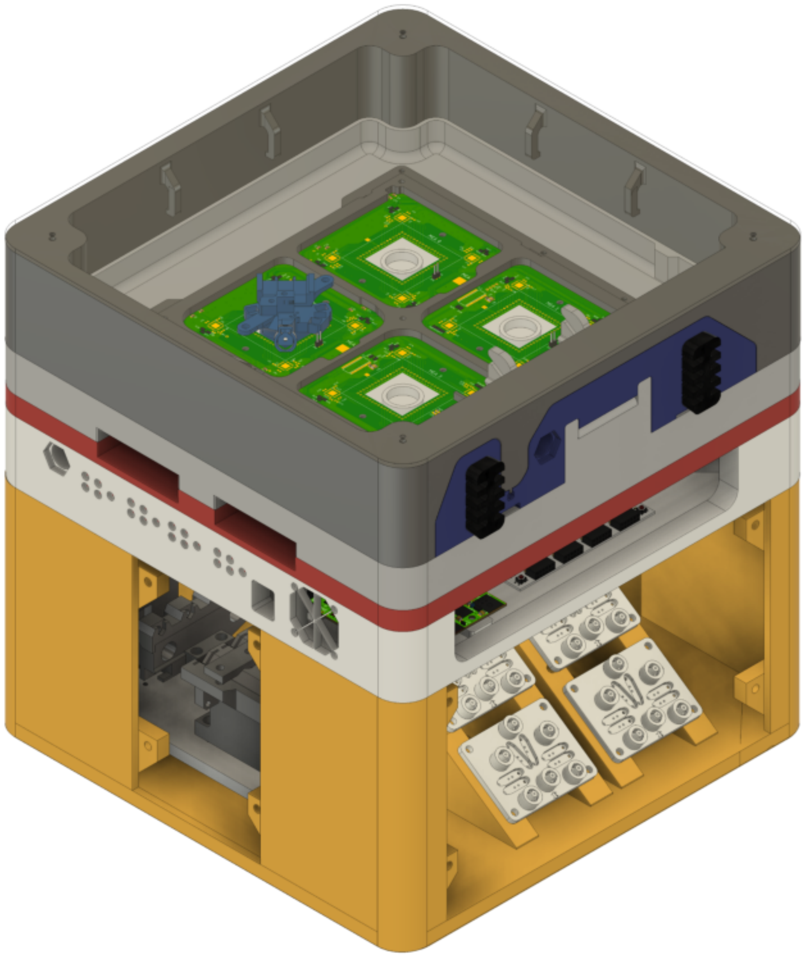

Figure A2 Side view of inkube layers in the CAD model

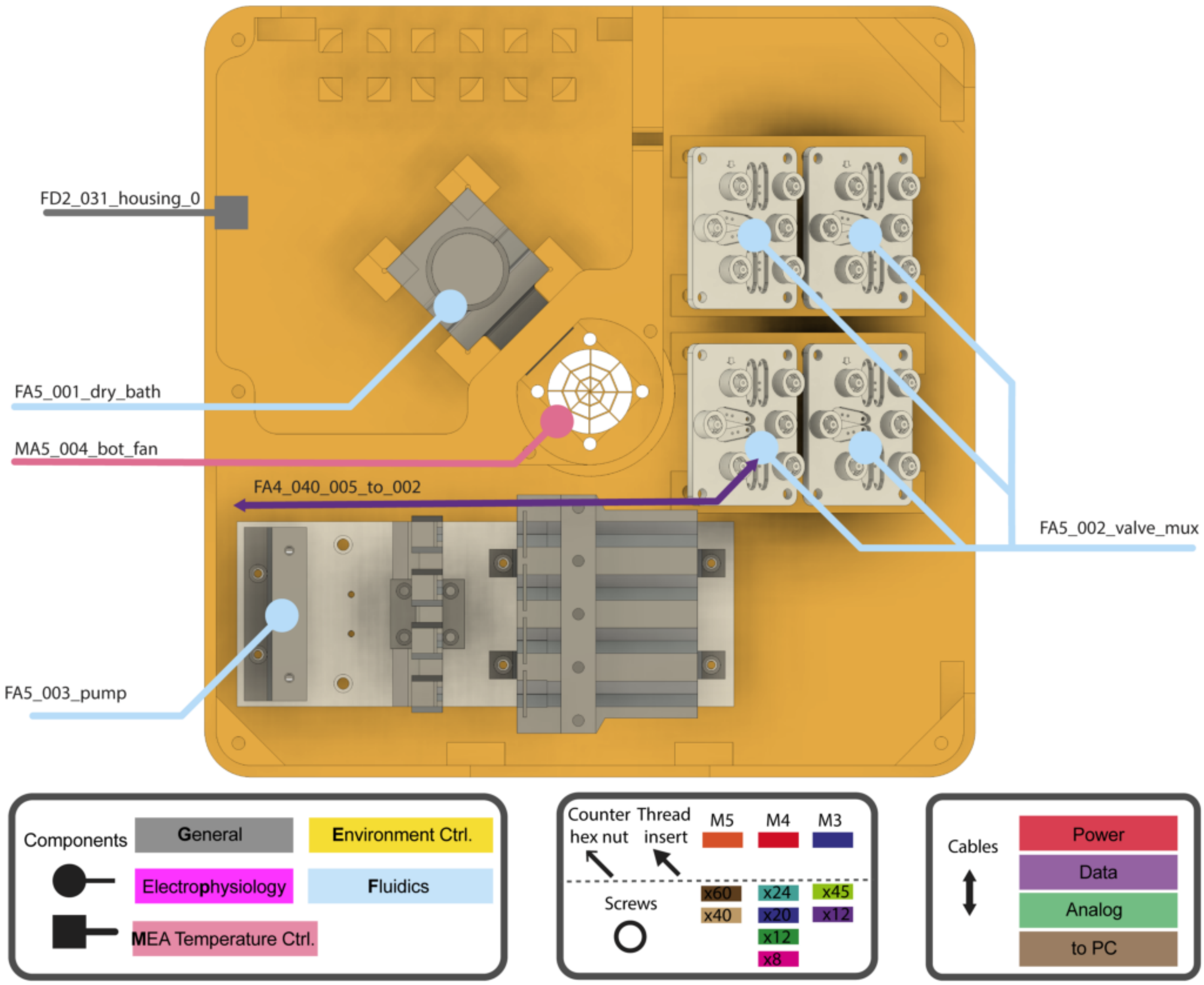

Figure A3 Fluidics layer (Layer 0) overview with components and cables

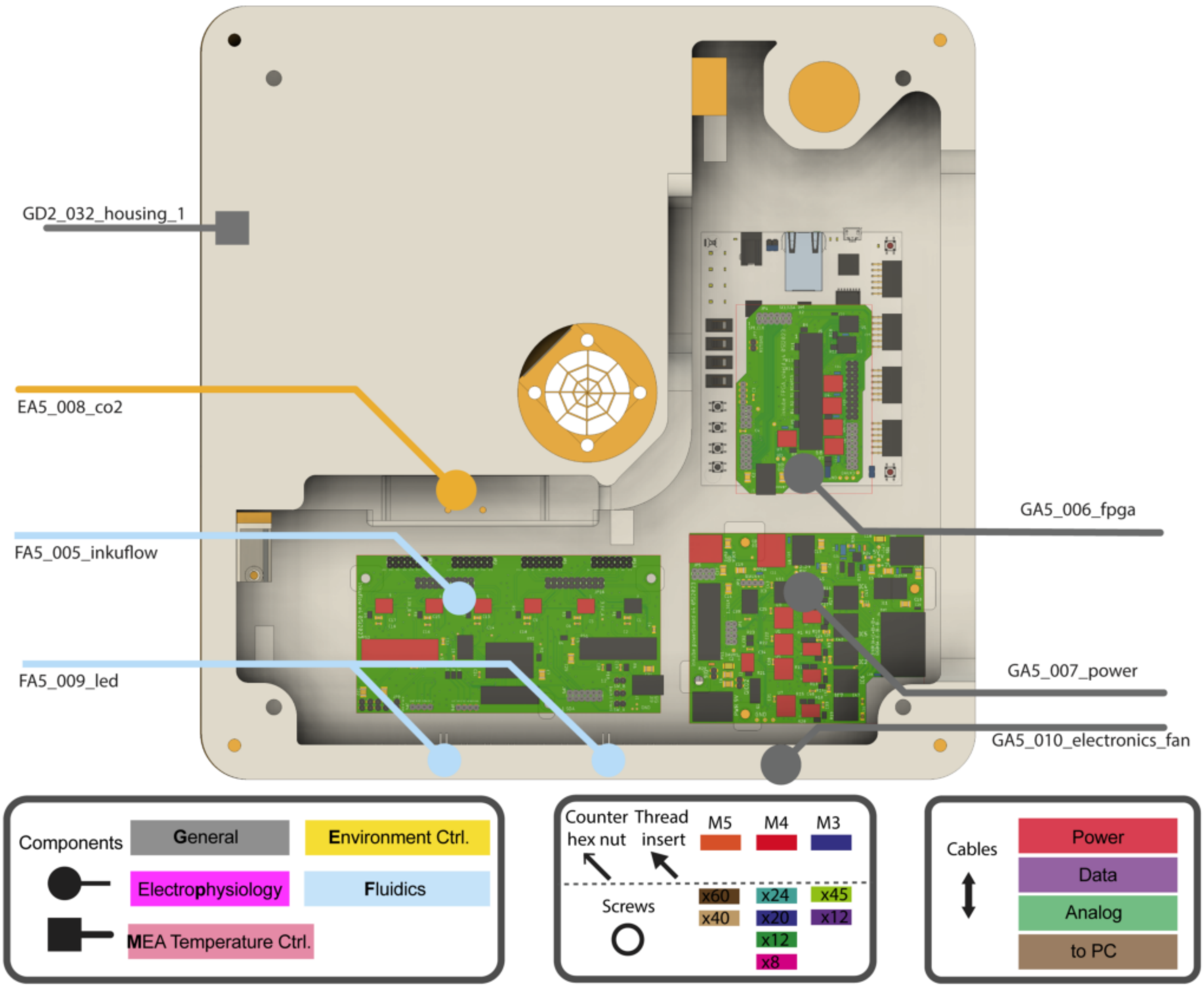

Figure A4 Electronics layer (Layer 1) overview with components

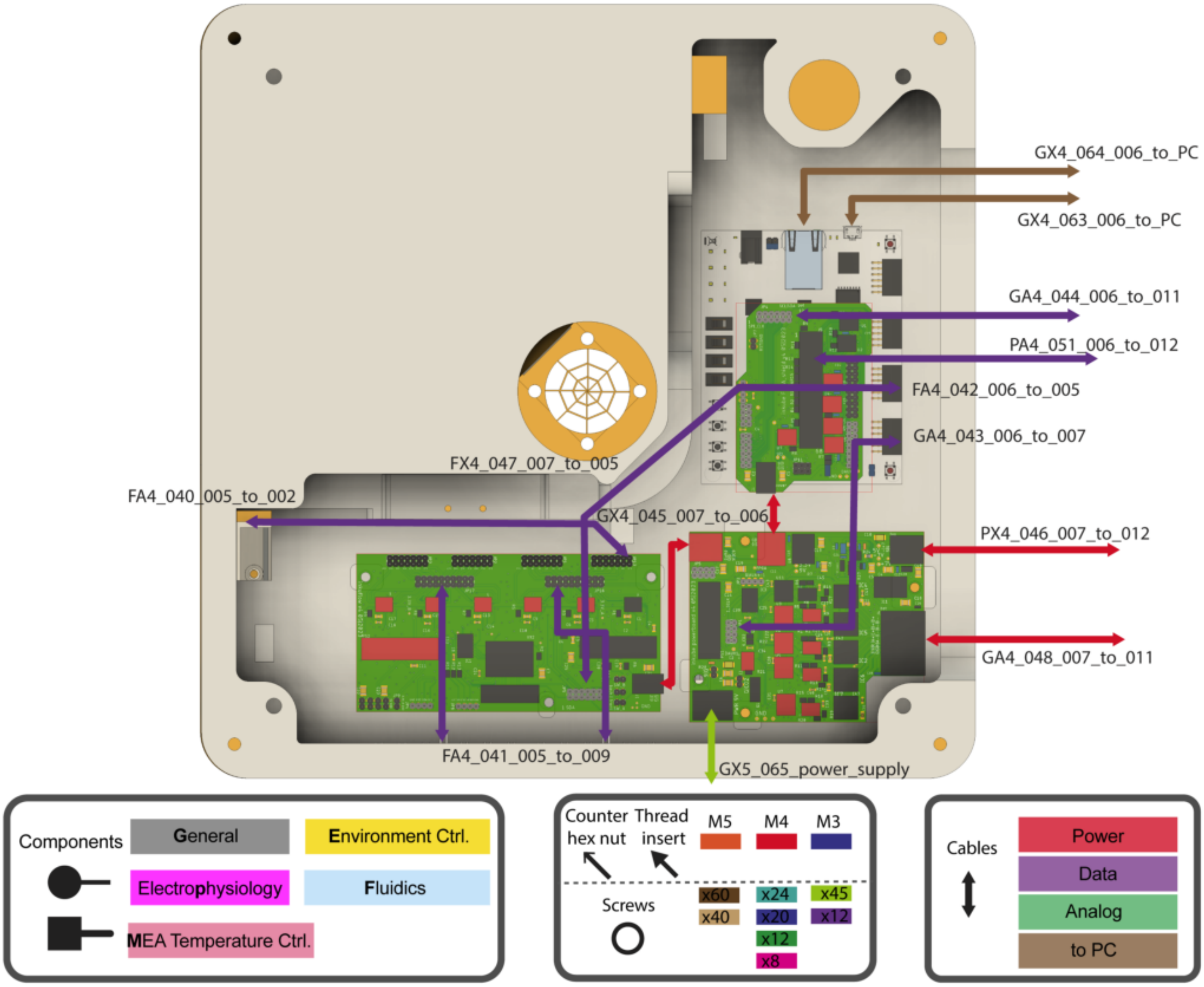

Figure A5 Electronics layer (Layer 1) overview with cables

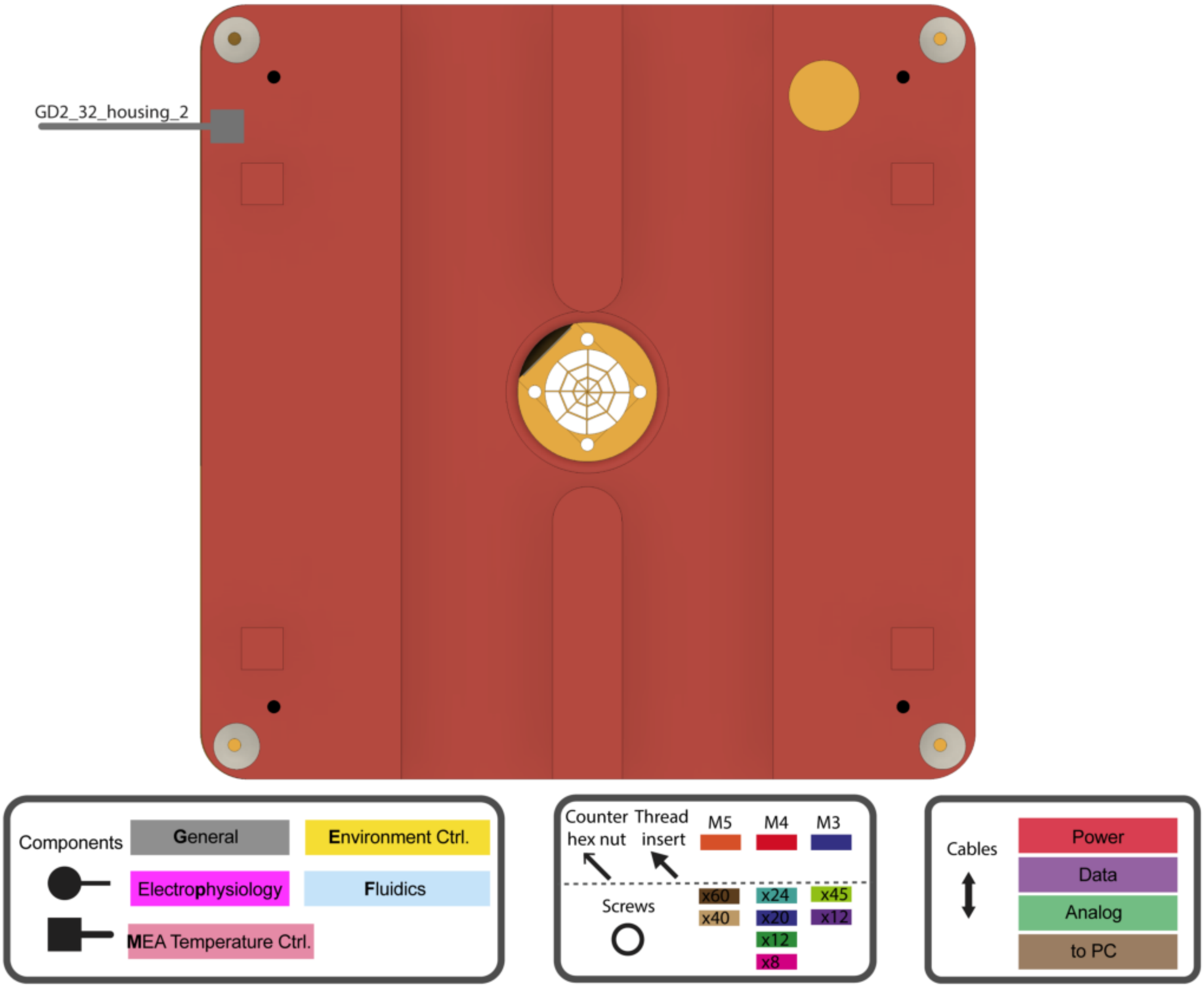

Figure A6 Ventilation layer (Layer 2) overview with components

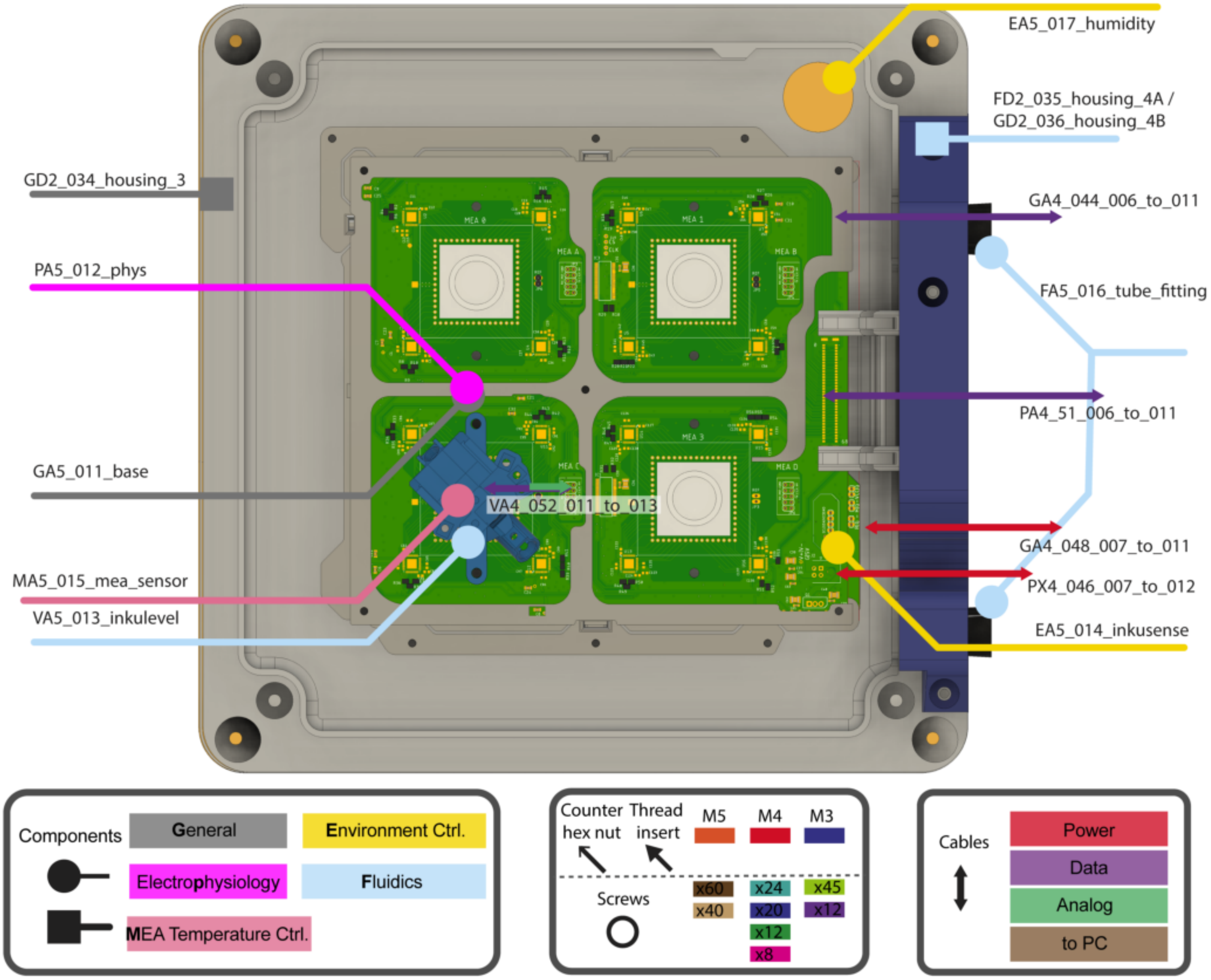

Figure A7 Incubation layer (Layer 3/4) overview with components and cables

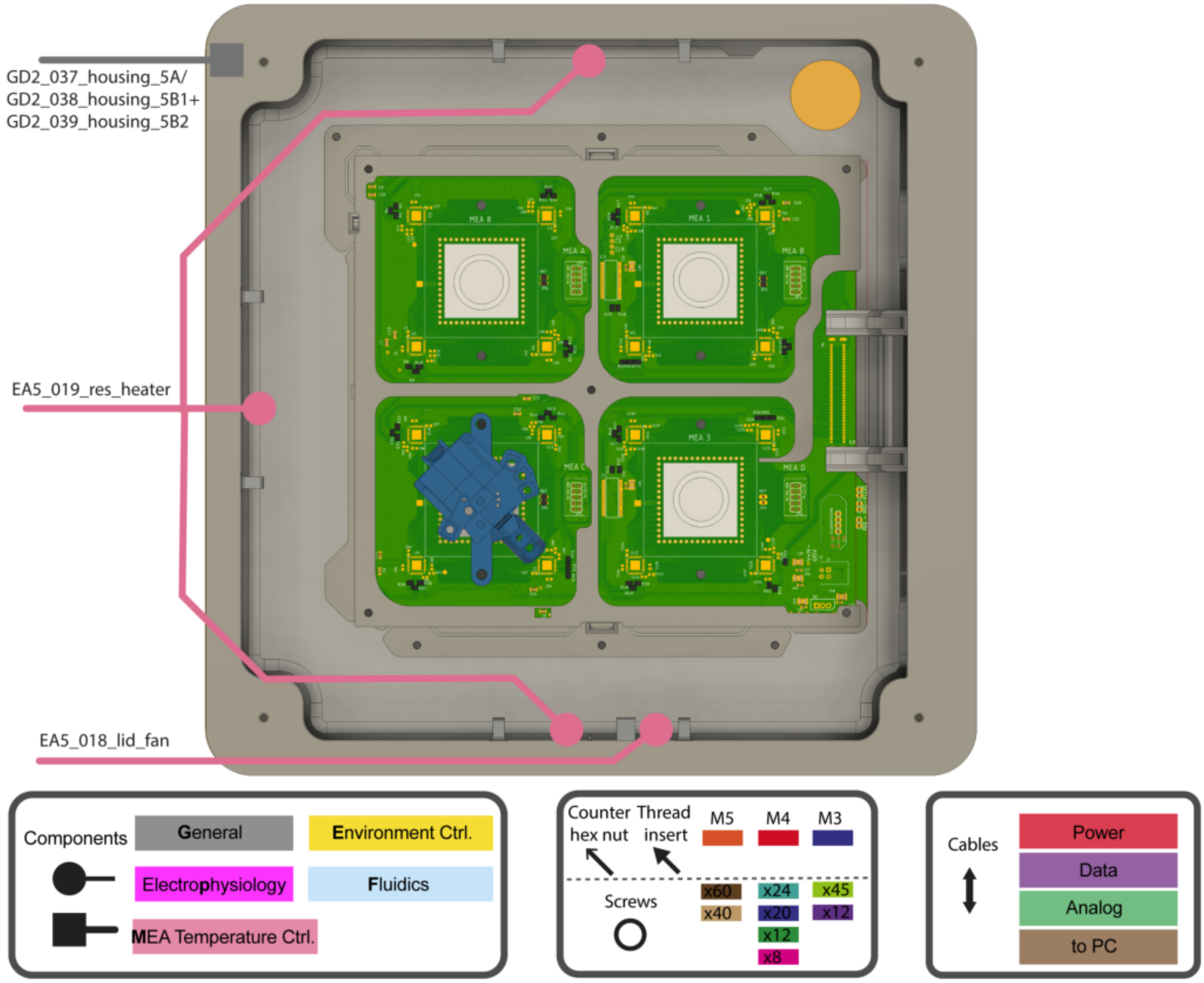

Figure A8 Lid overview with components

Next, the layers are shown with locations for hex nuts, thread inserts in the housing, and screws to attach assemblies to the housing.

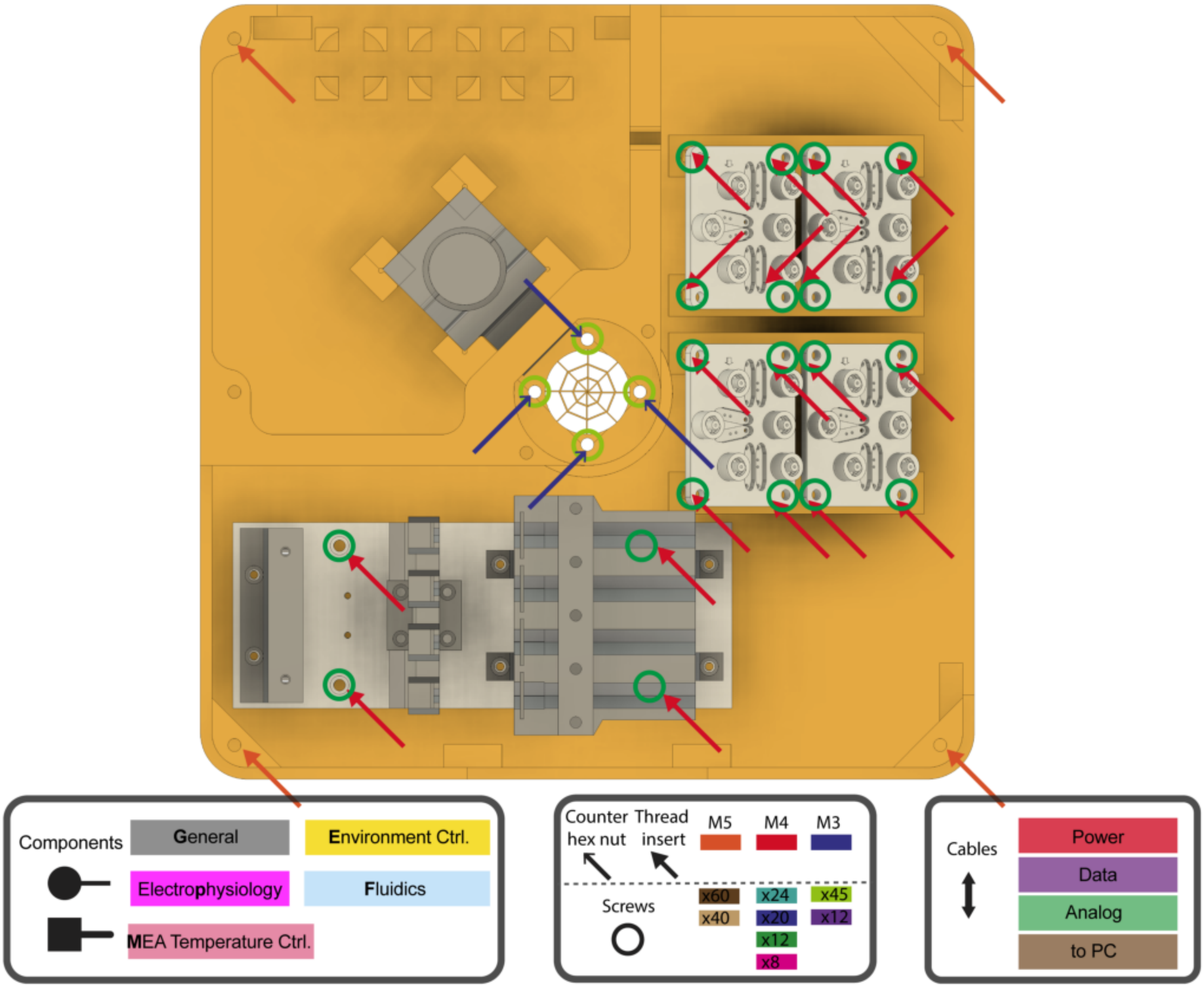

Figure A9 Fluidics layer (Layer 0) locations for screws

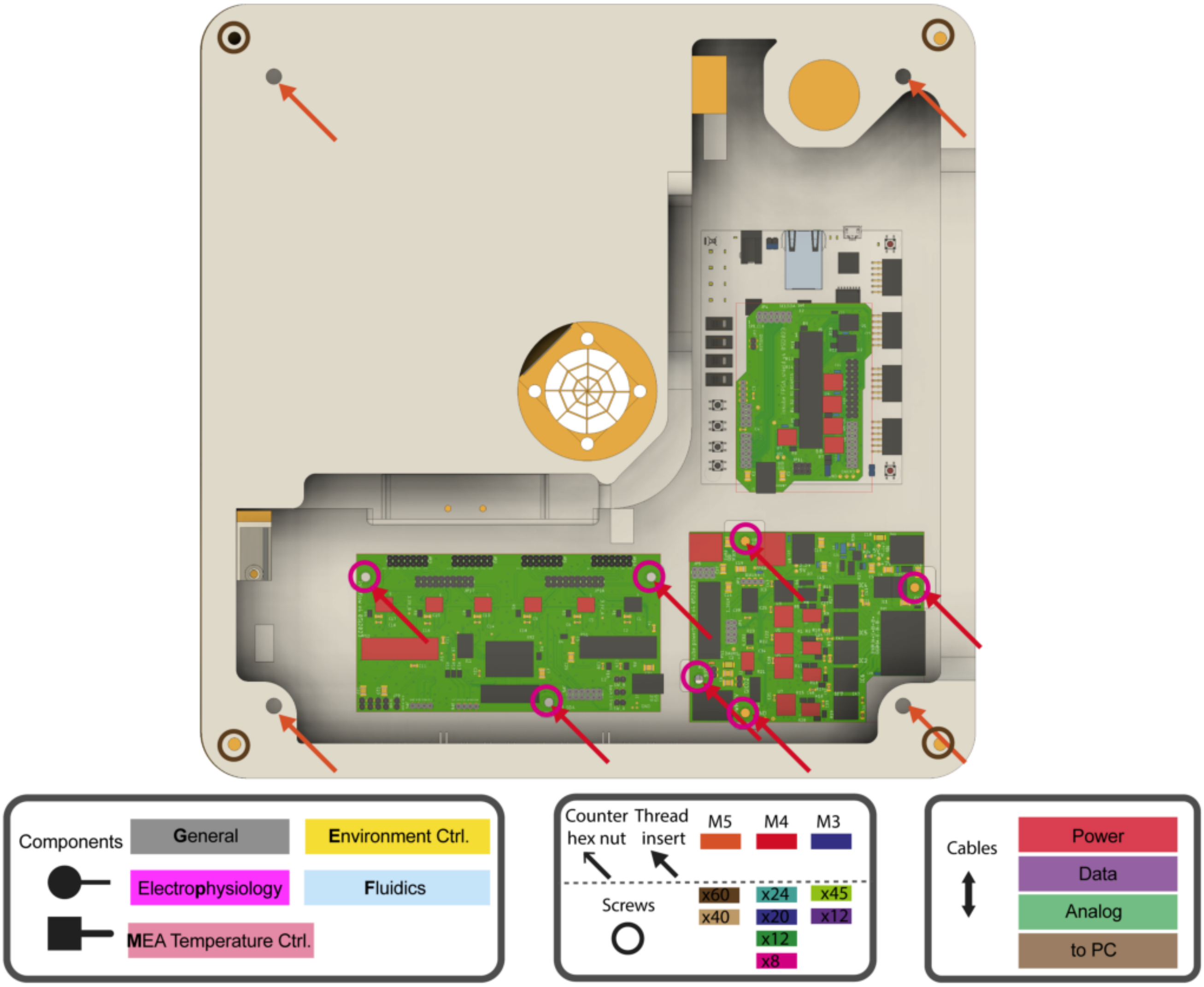

Figure A10 Electronics layer (Layer 1) locations for screws

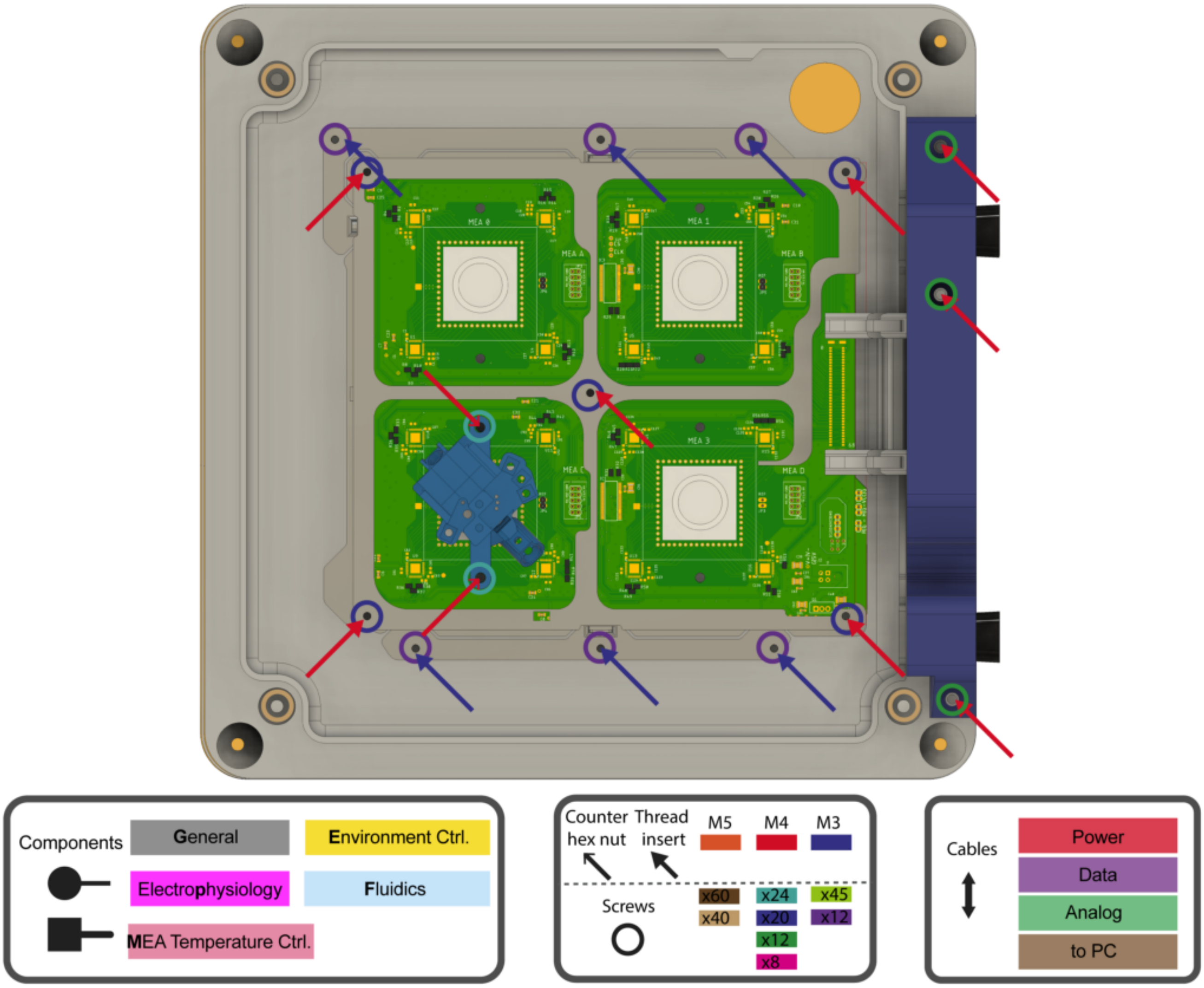

Figure A11 Incubation layer (Layer 3/4) locations for screws

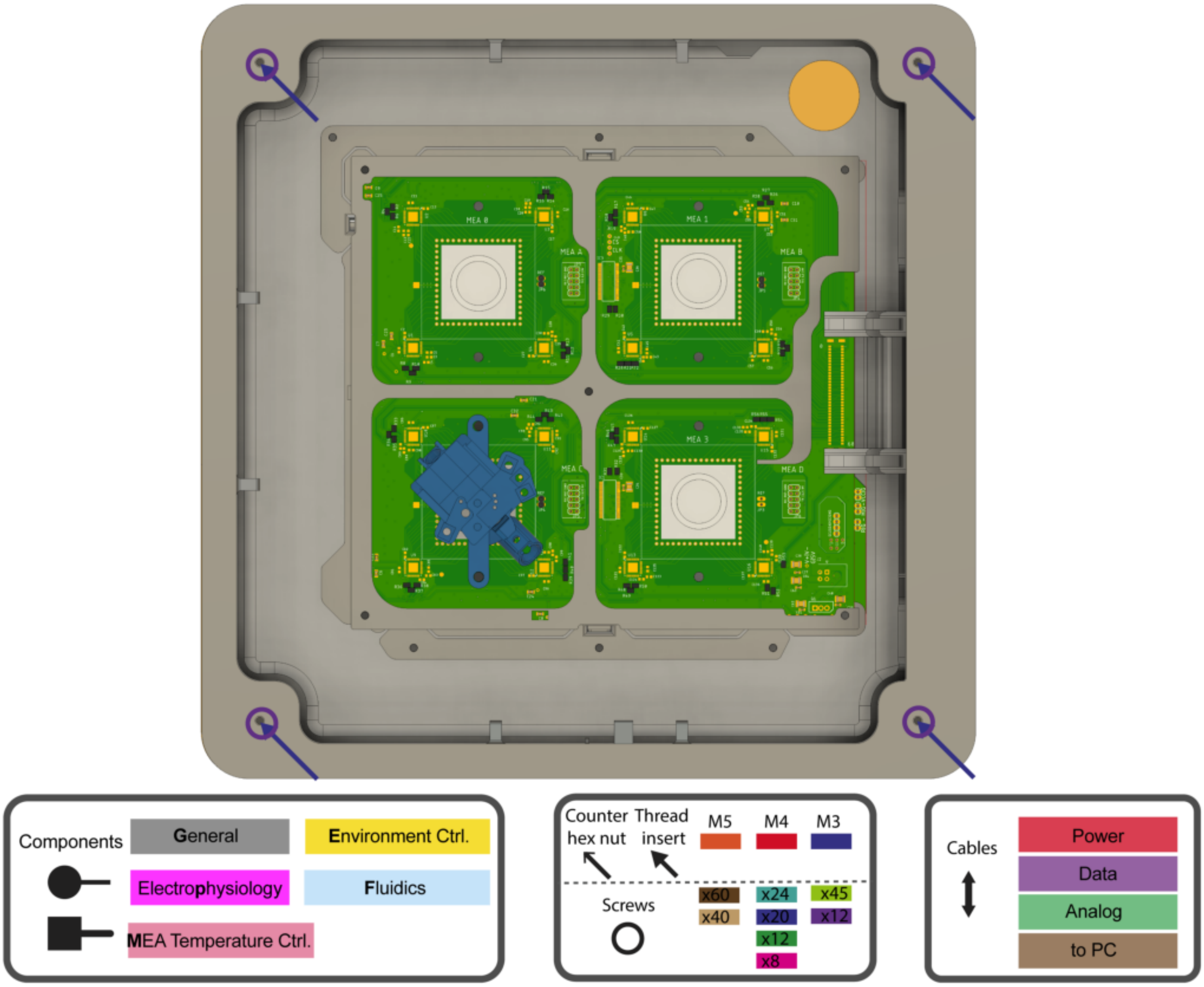

Figure A12 Lid locations for screws

D PCBs

In this section all PCB designs are shown. All boards are provided in version 4 of inkube. The unique identifier used in the parts list can be seen in the figure caption.

First the boards required for the general functionality are provided.

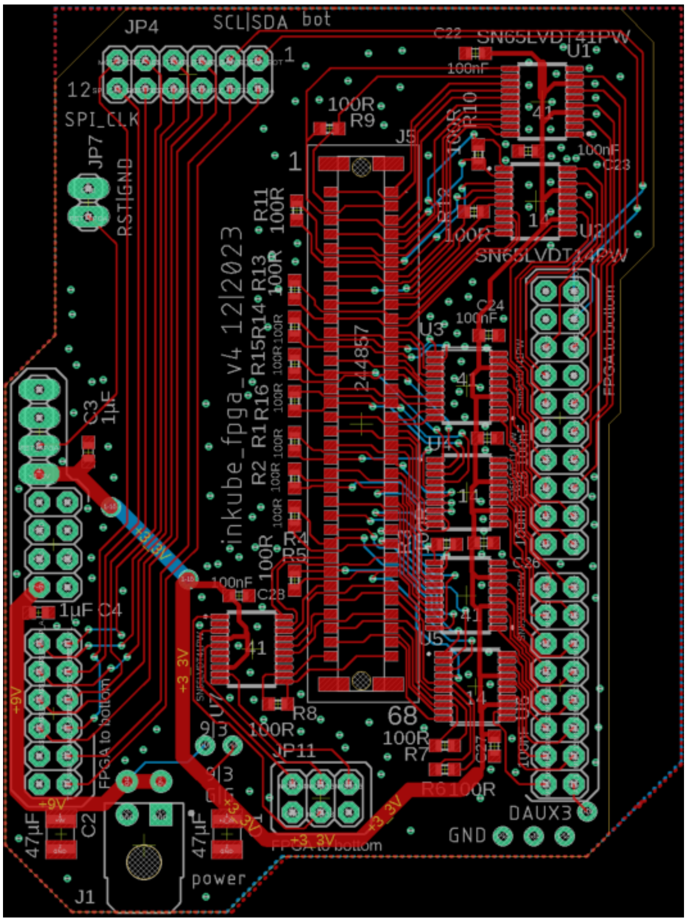

Figure A13 GD1_021_inkube_fpga_v4

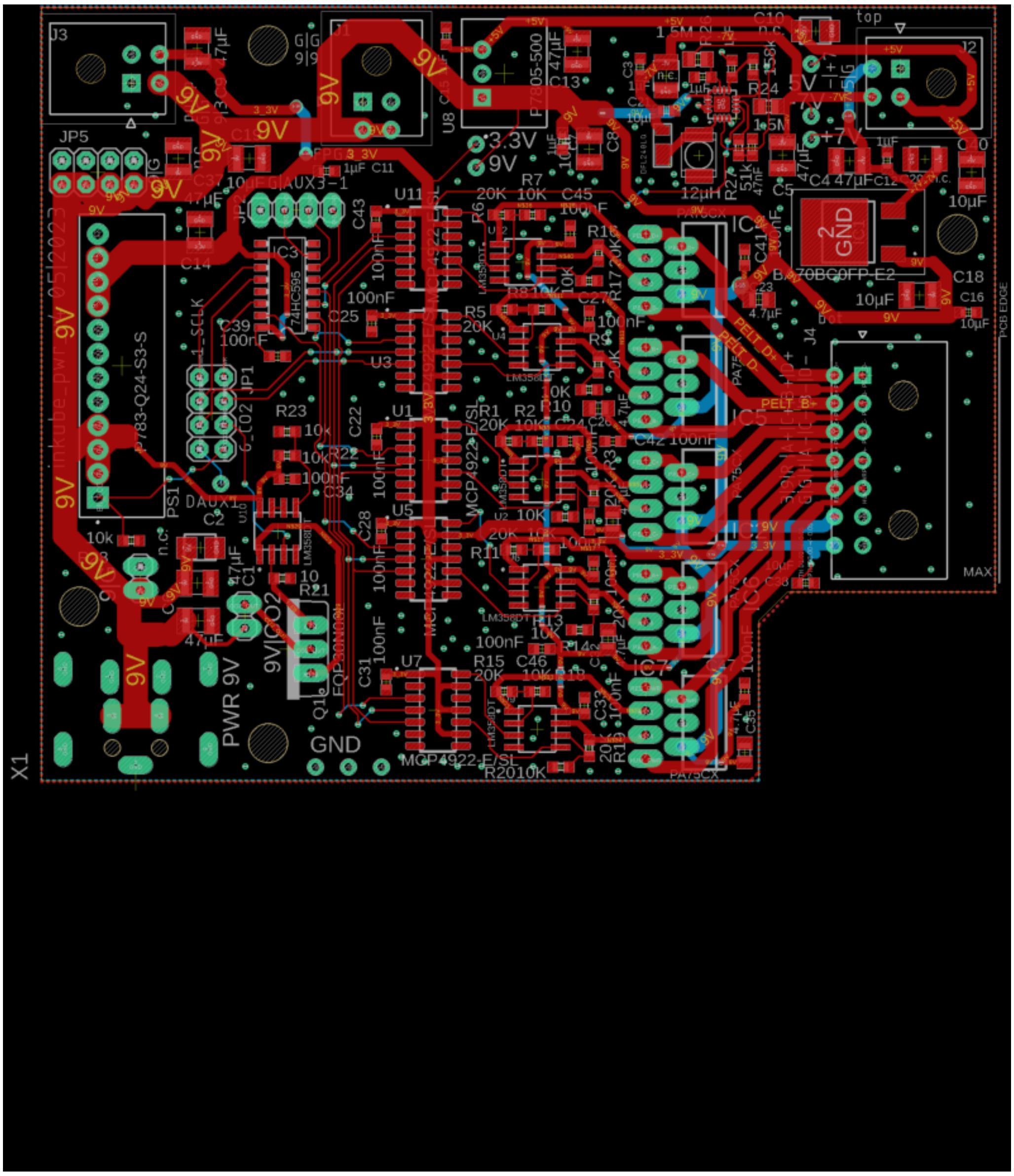

Figure A14 GD1_020_inkube_pwr_v4

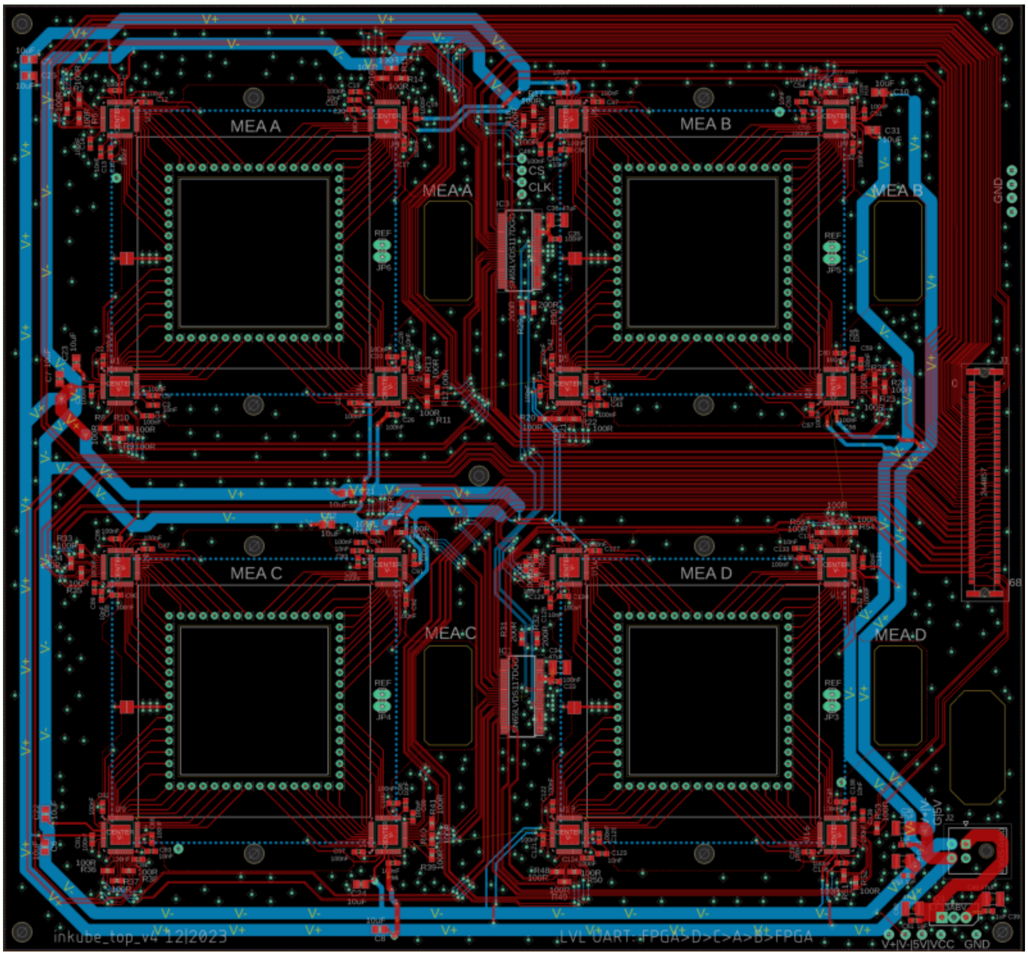

Figure A15 PD1_023_inkube_top_v4

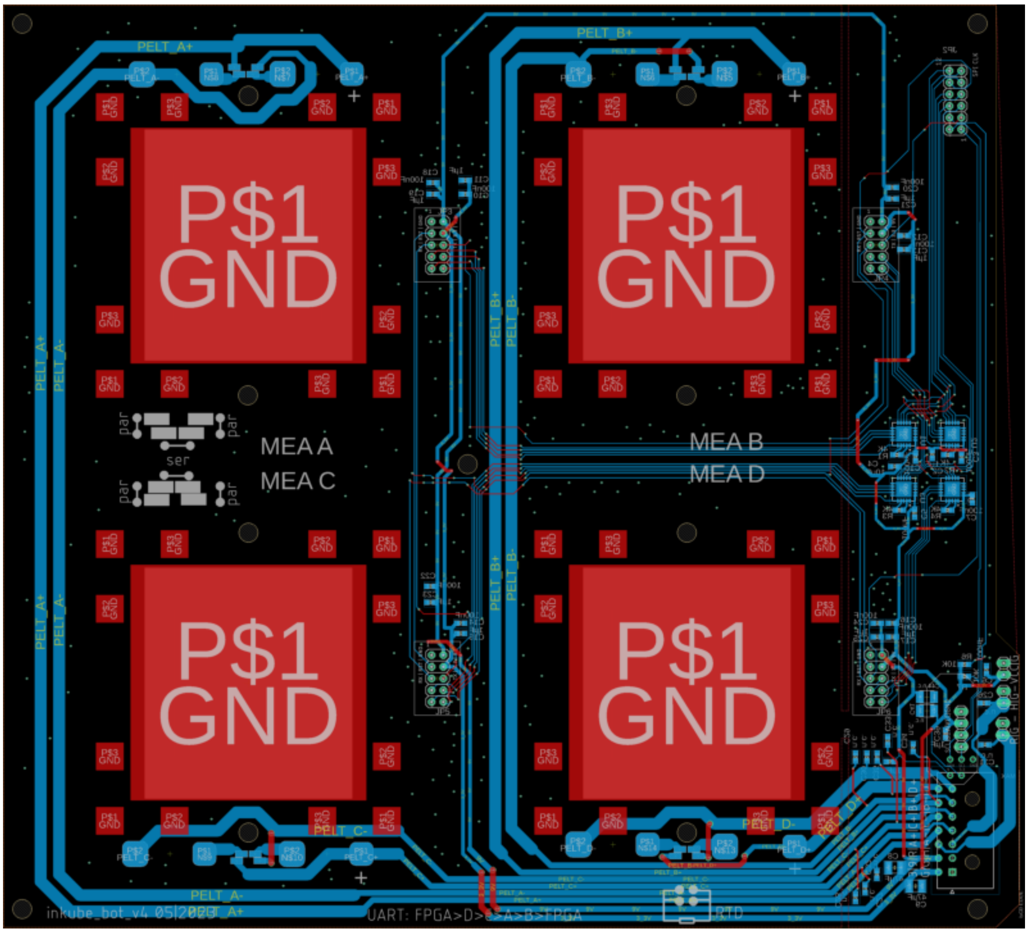

Figure A16 GD1_022_inkube_bot_v4

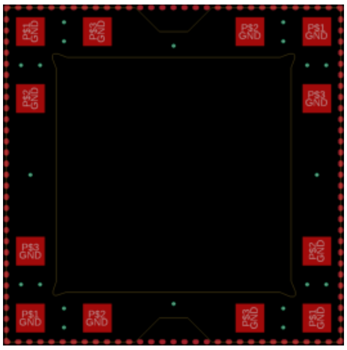

Figure A17 GD1_028_inkube_mea_v4

Here the boards for the fluidic system are listed.

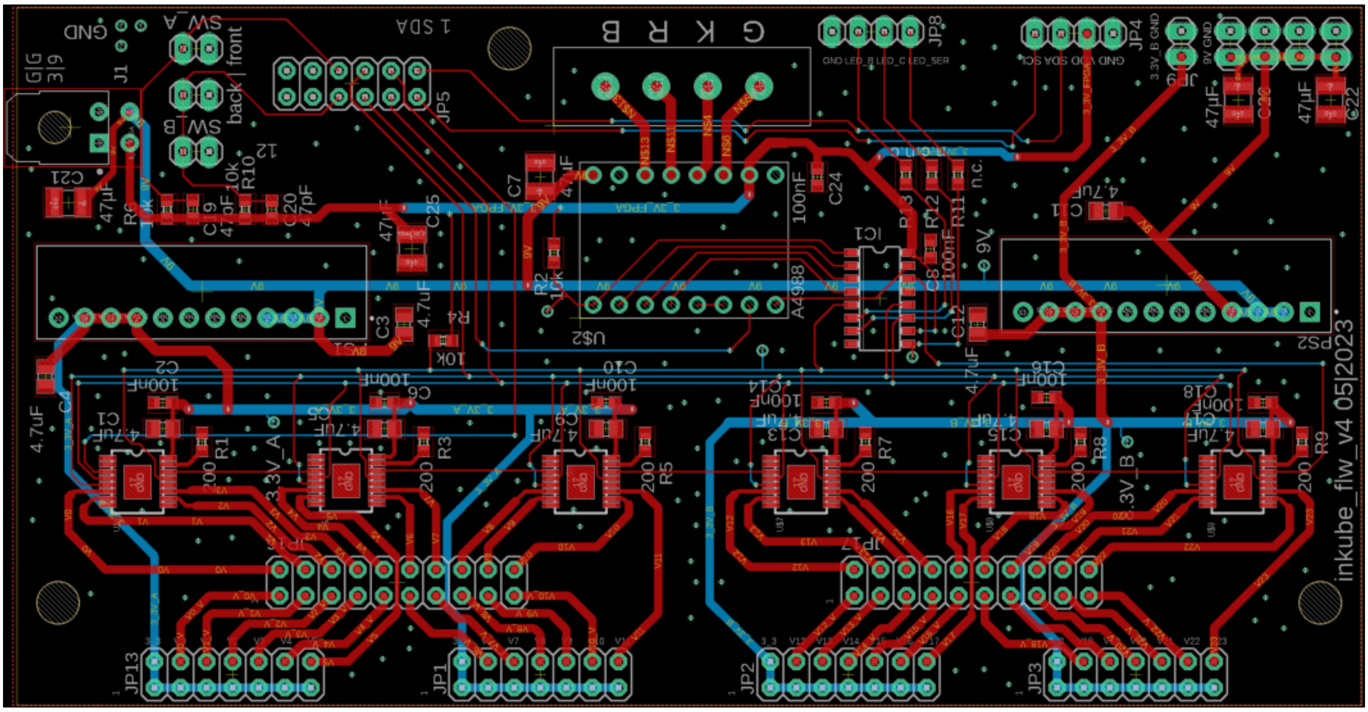

Figure A18 FD1_024_inkube_flw_v4

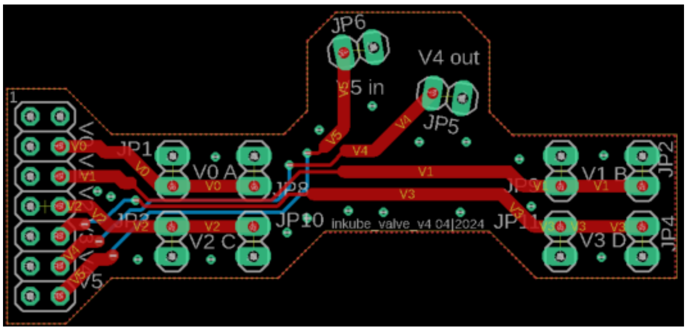

Figure A19 FD1_026_inkube_valve_v4

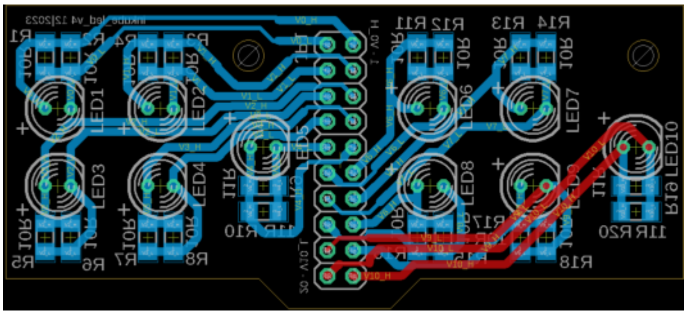

Figure A20 FD1_025_inkube_led_v4

Inkulevel is the only additional board required for volume feedback.

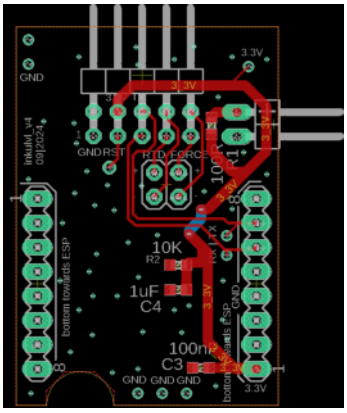

Figure A21 VD1_027_inkube_lvl_v4

E Housing

The assembly instructions are provided here for the general components, the housing, and the SoC shield.

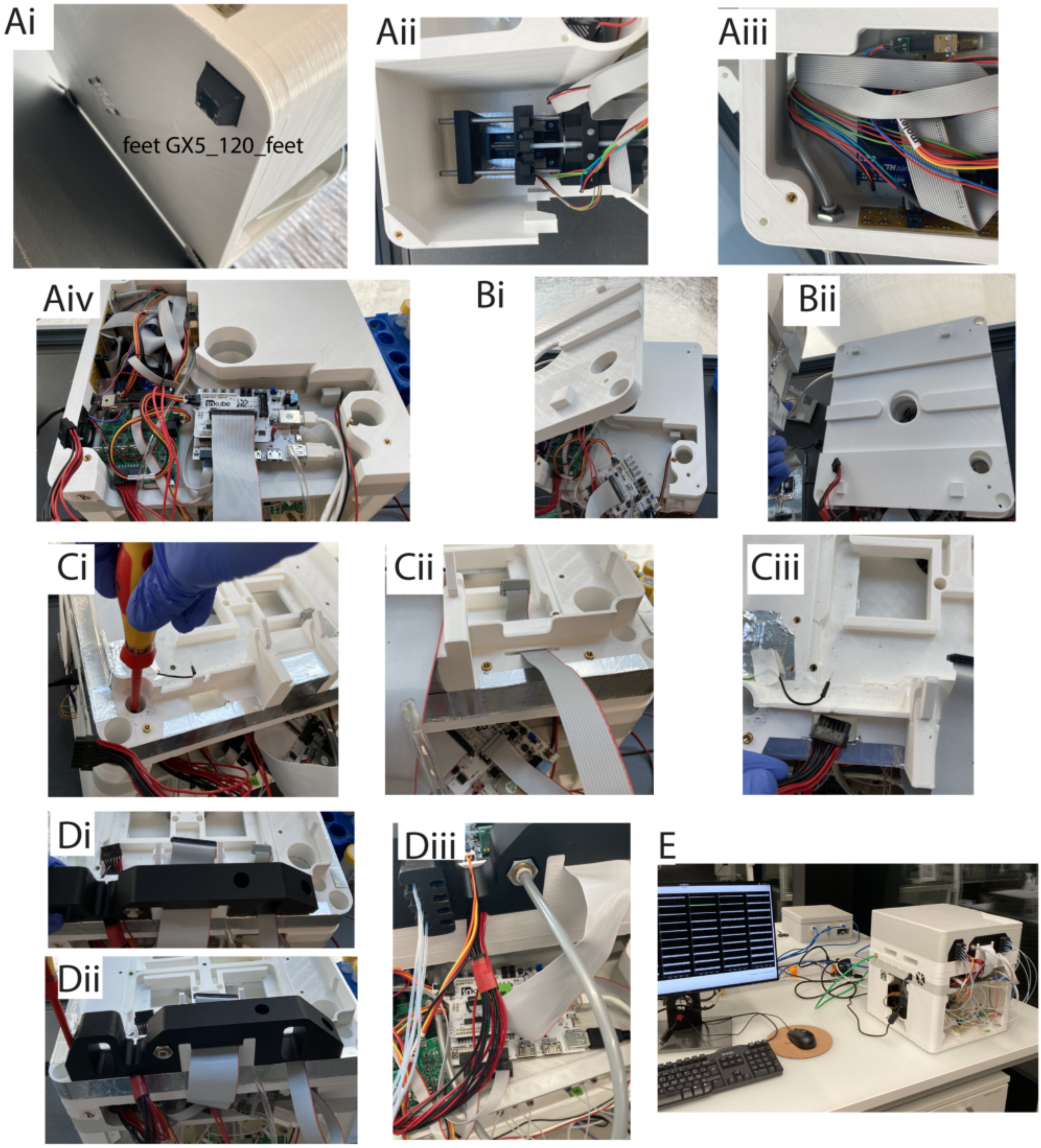

Figure A22 Housing assembly

To assemble the housing, prepare all layers with thread inserts and screws as shown in Fig. A9 to Fig. A12. The order in which to assemble the parts is listed here.

- Attach the feet as shown in Fig. A22Ai
- Assemble the fan as described in Fig. A26
- Assemble the dry bath as described in Fig. A34
- Assemble the pump as described in Fig. A29

- Place the pump as shown in Fig. A3 and Fig. A22Aii
- Mount the electronics layer and fix it with the outer screws
- Place the power board
- Place the inkuflow board
- Mount the electronics fan as described in Fig. A27
- Mount the CO_2 valve as described in Fig. A38
- Lead the cables for pump, dry bath, and fan up from the fluidics layer
- Attach the cables for the valve multiplexers and lead them down as shown in Fig. A22Aiii
- Attach all cables connecting the boards within the electronics layer
- Attach the cables to the host PC
- Attach the 2 power and 2 data cables leading up to the incubation layer as shown in Fig. A22Aiv
- Mount the ventilation layer as shown in Fig. A22Bi and Bii
- Mount the incubation layer and fix it with the inner screws as shown in Fig. A22Ci
- Lead the data and power cables for the bottom board through the gaps in the layer as shown in Fig. A22Cii and Ciii, respectively
- Lead the data cable for the top board through the gap
- Mount the incubation layer wall as shown in Fig. A22Di and Dii on top of the cable connections with the 3 inside screws
- Mount the base board and spacers as described in Fig. A24
- Mount the electrophysiology board as shown in Fig. A28
- Mount sensor board as shown in Fig. A24C
- Mount the CO_2 tube and the top board power cable as shown in Fig. A22Diii
- Mount the bottom layer valve multiplexers as shown in Fig. A3
- Mount the tube fittings as shown in Fig. A35
- Mount the lid fan as described in Fig. A40

To start an experiment complete these final steps:

- Remove the top board
- Place the MEAs as shown inFig. A28Di
- Mount the top board as shown in Fig. A28Dii and Diii
- Mount the inkulevel as shown in Fig. A36
- Connect the lid cables as shown in Fig. A40B
- Mount the lid (Fig. A22E)

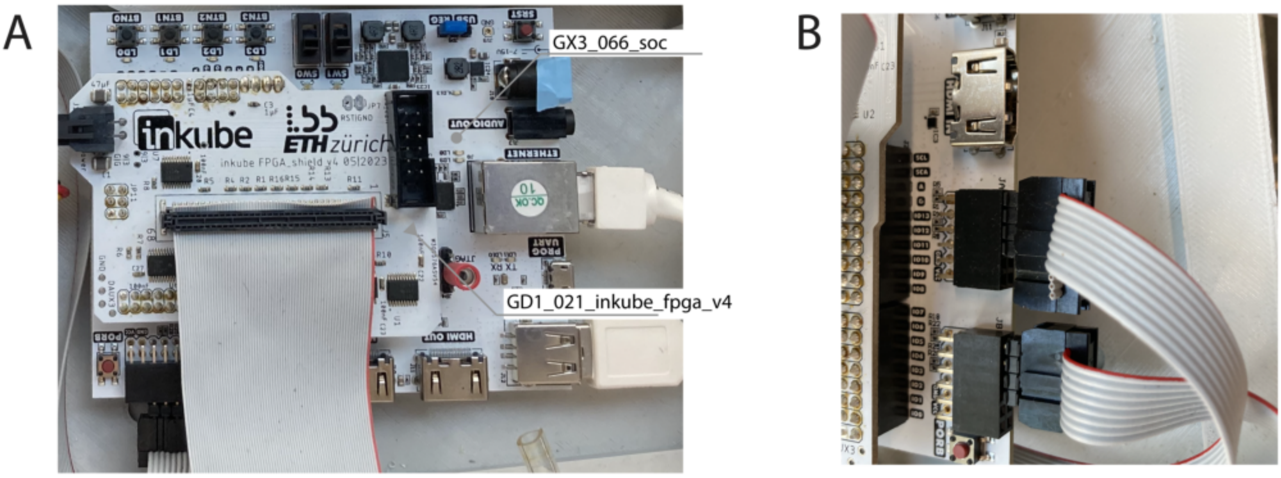

Figure A23 A Mount the shield and insert the PC cables, the data cable, and the power board cable **B** Insert the data cables for the power board and inkuflow. Pin 1 of the cables always goes to the top right pin of JA and JB.

F MEA Temperature Control

In this section the components for temperature control on the MEAs are listed.

F.1 Peltier mounting

The thermoelectric Peltier elements are mounted on the bottom board. The steps are shown in Fig. A24A.

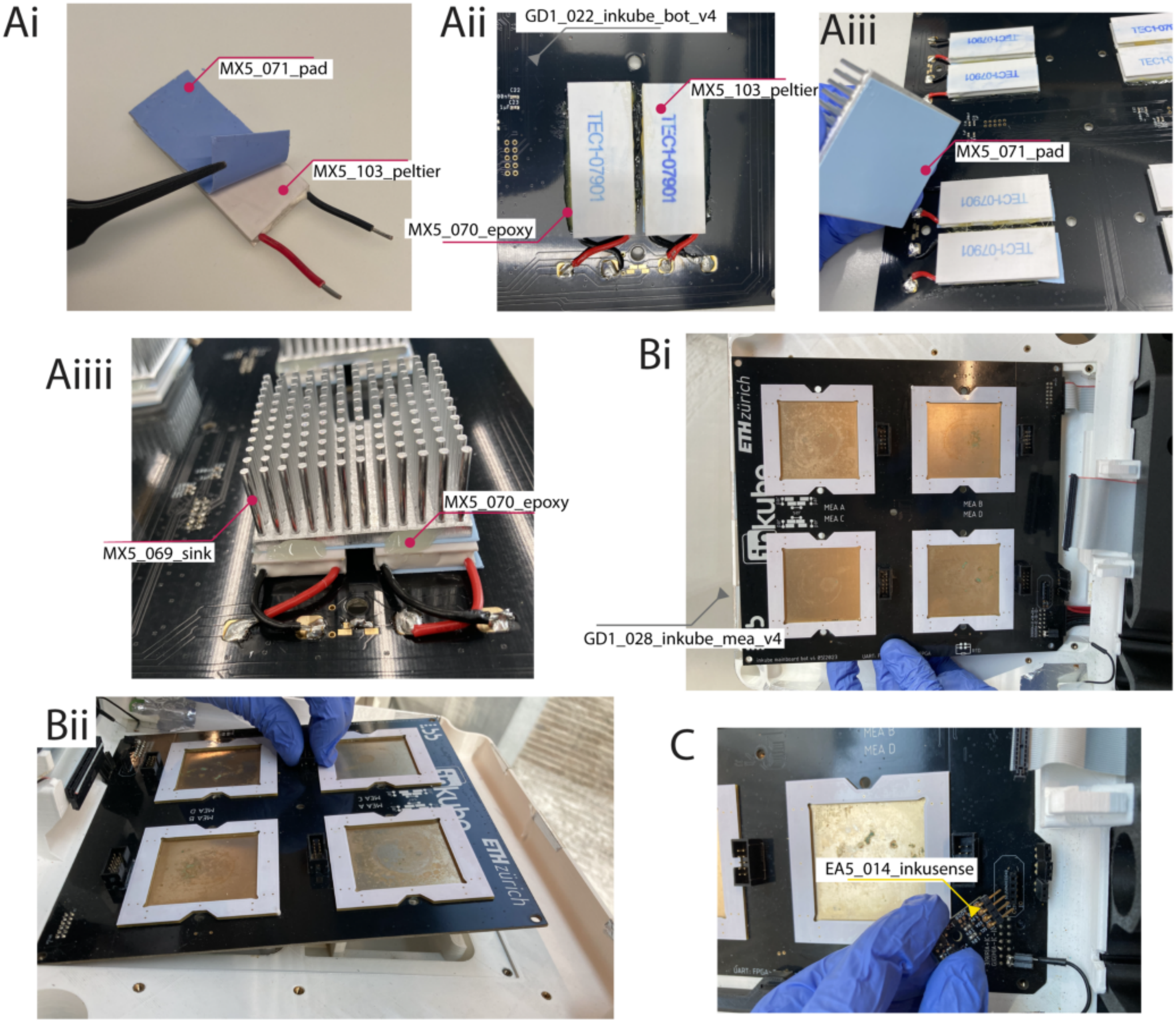

Figure A24 Ai) cut the cables to about 40 mm. Attach the thermal gap pad to the not-labeled side of the Peltier element. ii) Place Peltier elements on the markings of the bottom board. When the writing points up, connect the red cable to the pad marked with '+' and the second device with similar polarity. For standard operation, shorten the two closer pads horizontally as shown on the bottom board print to use the thermoelectric devices in serial connection. Use the epoxy glue to fix them. iii) mount the heat sink with another piece of the gap pad. iv) Use the epoxy for fixation. **B**i) connect the data and power cable. ii) Slide the board in place from the side opposite of the cable connections. **C** Connect sensor board.

F.2 RTD assembly

The PT1000 temperature sensor is wrapped in a biocompatible tubing and connected in a 4-point measurement. The steps are shown in Fig. A25.

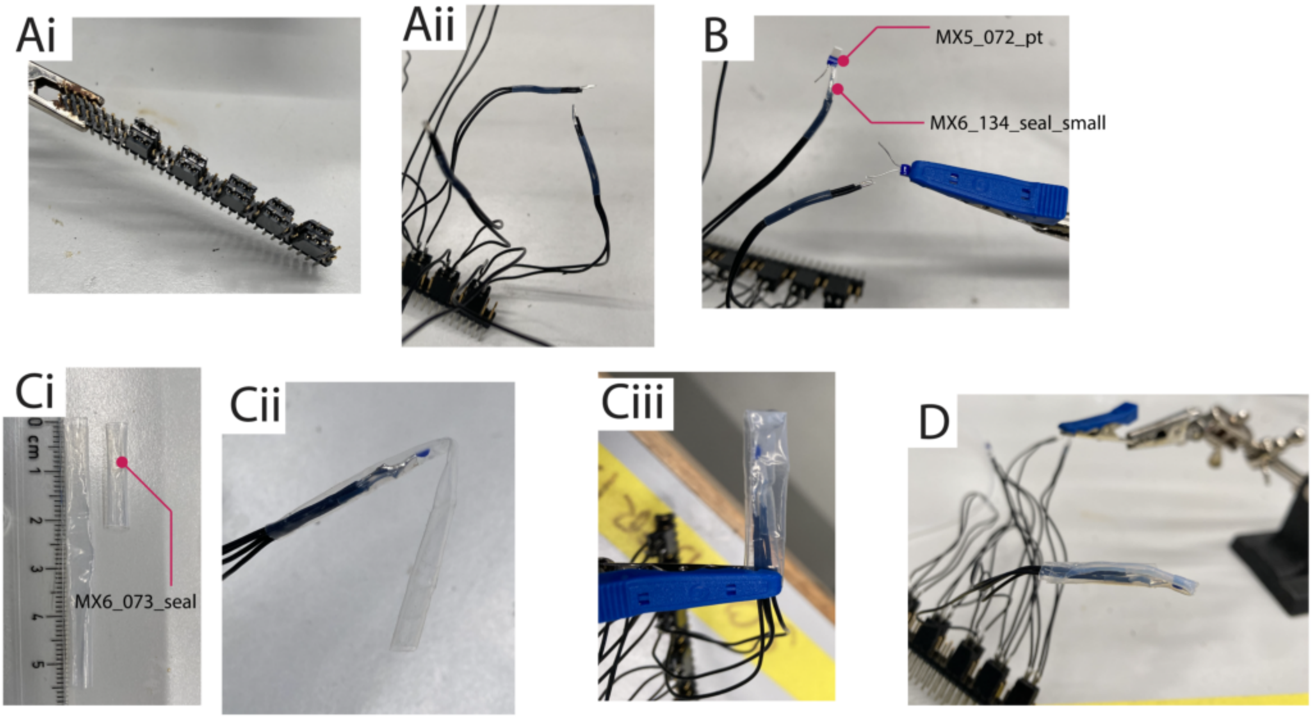

Figure A25 **A**i) Cut a standard 2.54 mm spaced 2 by 2 female header. ii) Attach 4 wires to the the pins (small diameter and heat resistant PTFE insulation recommended). **B** Connect 2 of the wires to one contact of the PT1000, insulate it with shrink tubing, and connect 2 wires to the other contact. **C**i) Cut 2 pieces of biocompatible shrink tubing. ii) Push one half of the long tube over the sensor and fold it to the side. iii) Push the short tube over the folded tube to hold it in place. **D** Use a heat gun at 400 °C to shrink the tube. Take care to not melt the solder tin.

F.3 Fan for heat sink and Peltier driver cooling

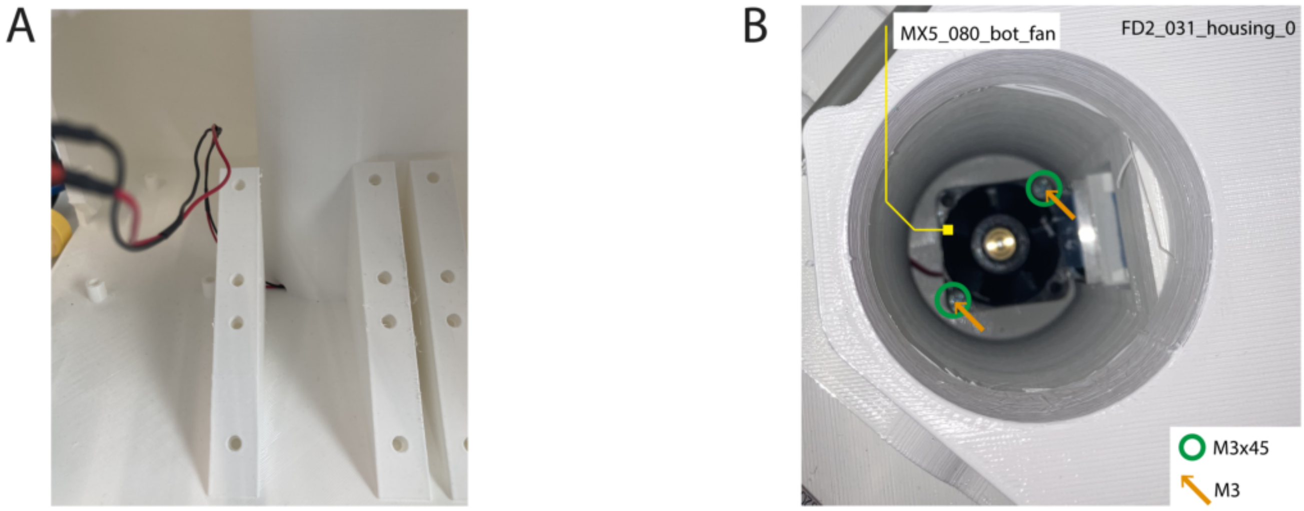

Figure A26 A Cut the fan wire and guide it out through the whole. Attach an extension to reach a 9 V connector on the power board. **B** Place the fan and insert the screws from the top. Counter the screws with a hex nut on the other side. Ideally use a magnetic screw driver to bring the screws in place.

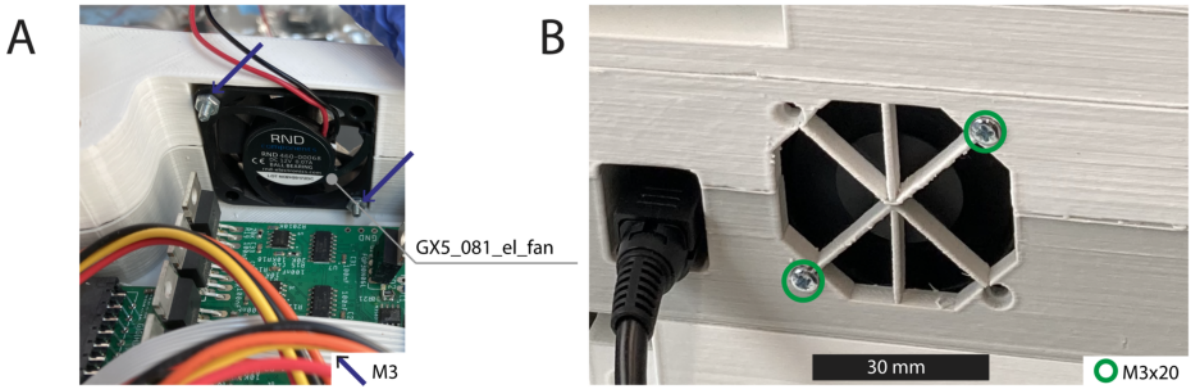

Figure A27 A Place the fan and attach a connector to the cables to plug it in to some 9 V power connector on the inkuflow or power board. **B** Insert the screws and use a hex nut as shown in **A** to counter it.

G Electrophysiology

G.1 Electrophysiology board

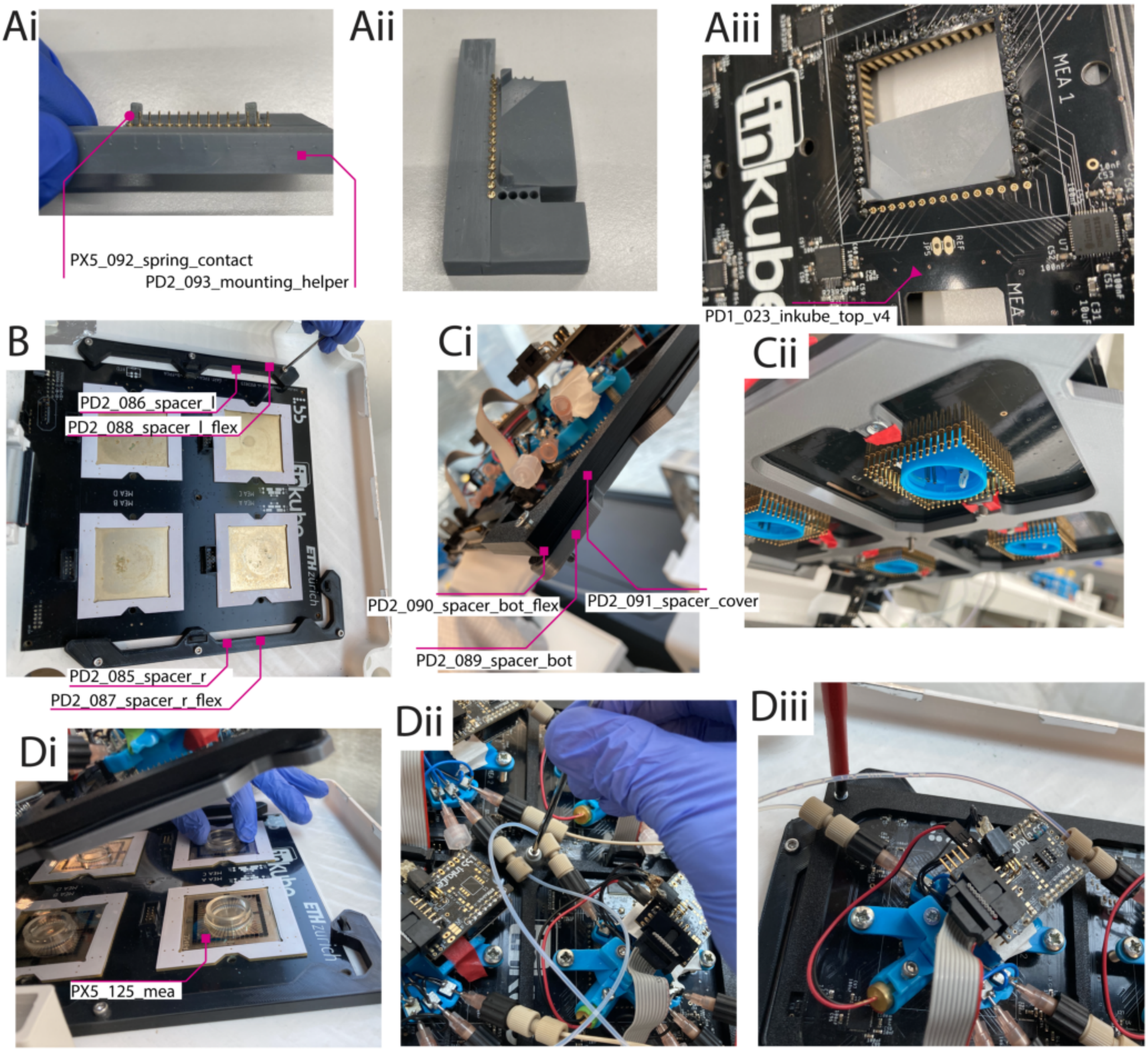

Figure A28 **A**i) Assemble the two parts of the mounting helper. ii) Use tweezers to place all pins such that the top rim sticks out. iii) Place the helper under the top board and solder the pins. Use low temperatures for soldering (depending on the solder tin 300 °C). **B** Mount the spacers on top of the bottom board. **C**i) Mount the spacers to the top board. ii) Insert the hex nuts to the top board spacer. Depending on printing tolerance hold them in place with some tape. **D**i) Slide the top board into the tilted slit on the side of the cable connections. Place the MEA. Take care of the reference electrode location. ii) Place the top board. Start tightening the center screw. iii) Continue with the corner screws.

H Fluidics

H.1 Pump

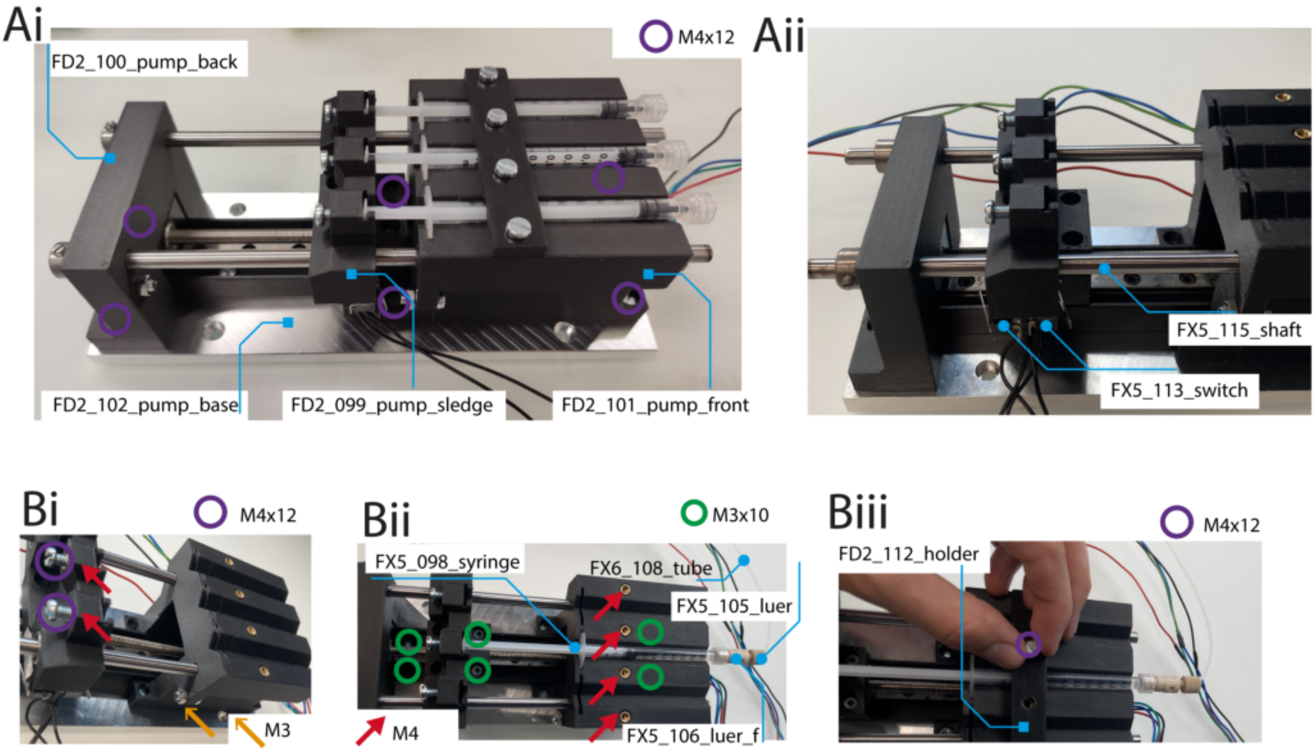

Figure A29 **A**i) Mount linear motor stage to base plate. ii) Mount the front part. Glue the safety switches to the sledge. Mount the sledge. Mount the back part. Insert shafts. **B**i) Mount screws to fix syringes. ii) Insert syringe. iii) Mount holder to hold syringes in place.

H.2 Multiplexing scheme

In Fig. A30 the multiplexing scheme of inkuflow is described. For each liquid a new path consisting of a syringe, a multiplexer, and a reservoir is required and for every MEA an extra port on the multiplexer is needed. Every multiplexer has one port for the syringe, which is connected through one valve to a reservoir, and an arbitrary number (in this case 4) valves with ports for the MEAs. The multiplexers always have a single valve open while the pump is being moved.

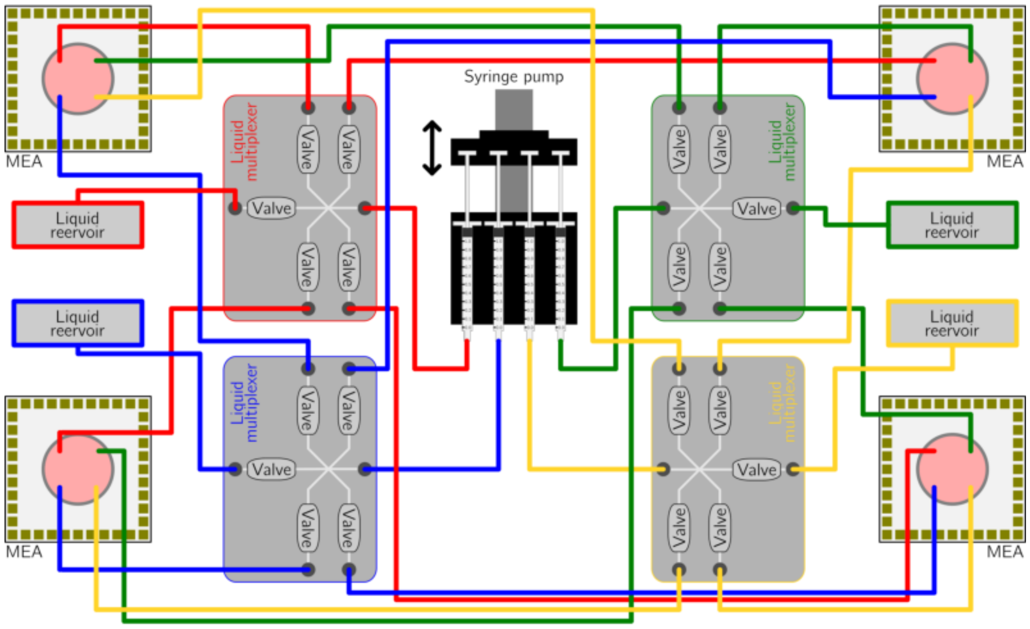

Figure A30 Detailed block diagram of the inkube perfusion. The perfusion system of inkube consists of 4 liquid reservoirs, 4 liquid multiplexers, and a single syringe pump. Pathways corresponding to the same liquid are shown in the same color.

H.3 Valve multiplexer

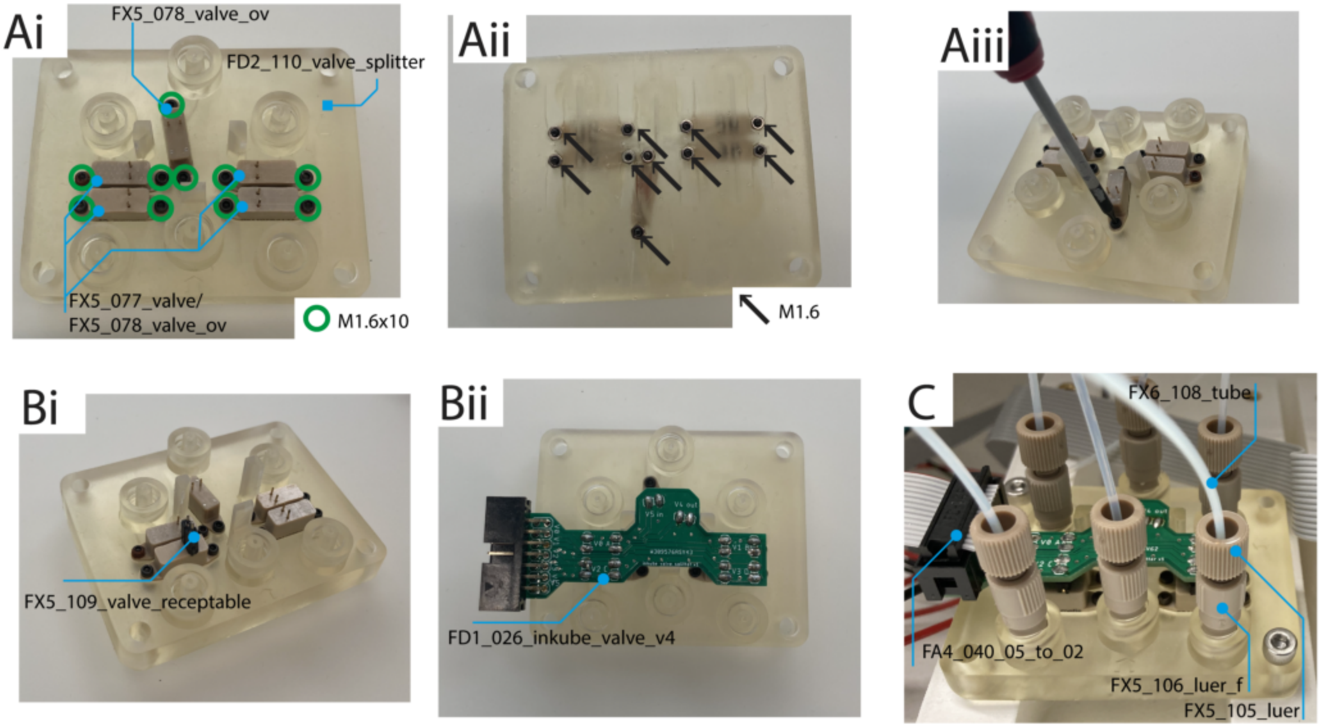

Figure A31 **A**i) Place valves on multiplexer. ii) and iii) Insert screws and counter with hex nuts. **B**i) Place preci pins (from valve PCB). ii) Mount PCB and solder connections. **C** Connect tubing through Luer locks.

H.4 Driver board and status LEDs

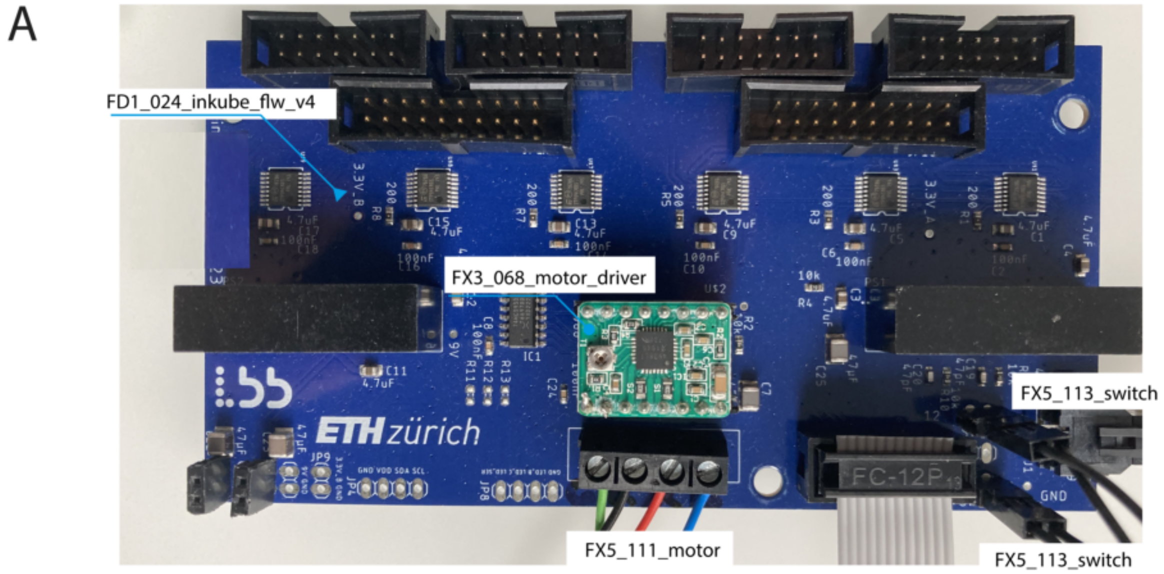

Figure A32 inkuflow board. Mount the motor driver PCB. Screw in the cables for the motor according to the labeling on the PCB. Connect safety switches for front and back according to labeling.

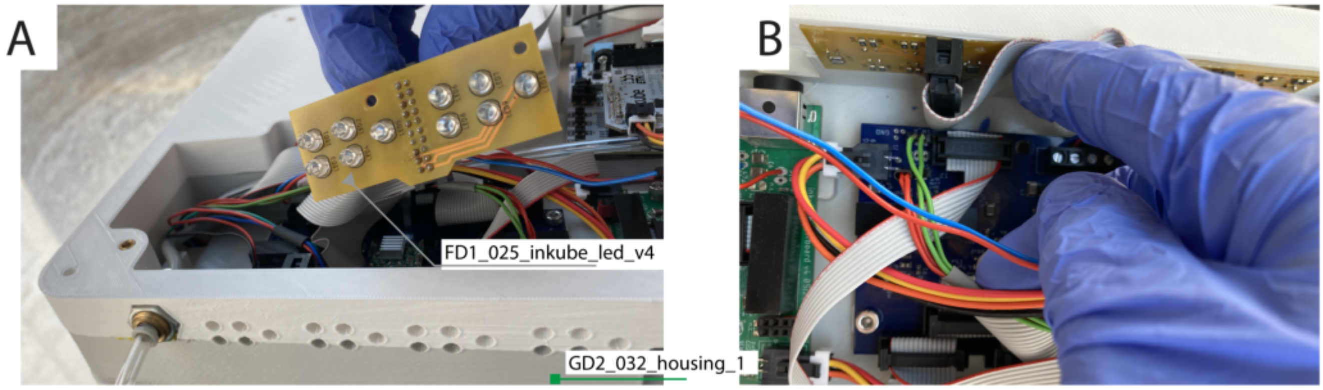

Figure A33 A Connect cable to back of assembled PCB. **B** Push PCB with LEDs into the wall of the electronics layer.

H.5 Liquid reservoir

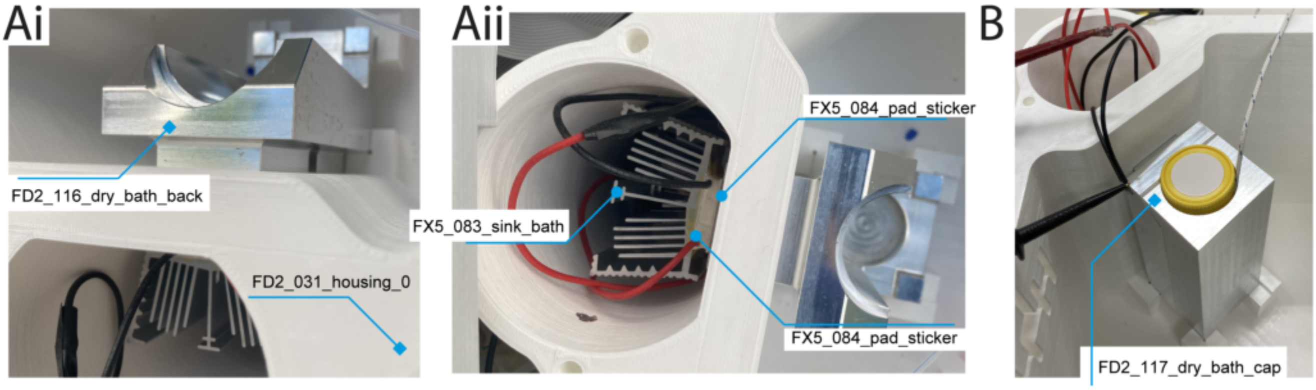

Figure A34 **A**i) Attach stickers to dry bath back. Insert back part through holes to ventilation channel in the fluidics layer. ii) Mount a second set of stickers on the Peltier elements. Mount the Peltier elements on the stickers of the dry bath back with the labeled side and the cables pointing upwards. **B** Mount the 50 mL falcon tube and place dry bath cap. Optionally add the insulation shield (FD2_130_dry_bath_cover) to prevent condensation.

H.6 Tubing and connection

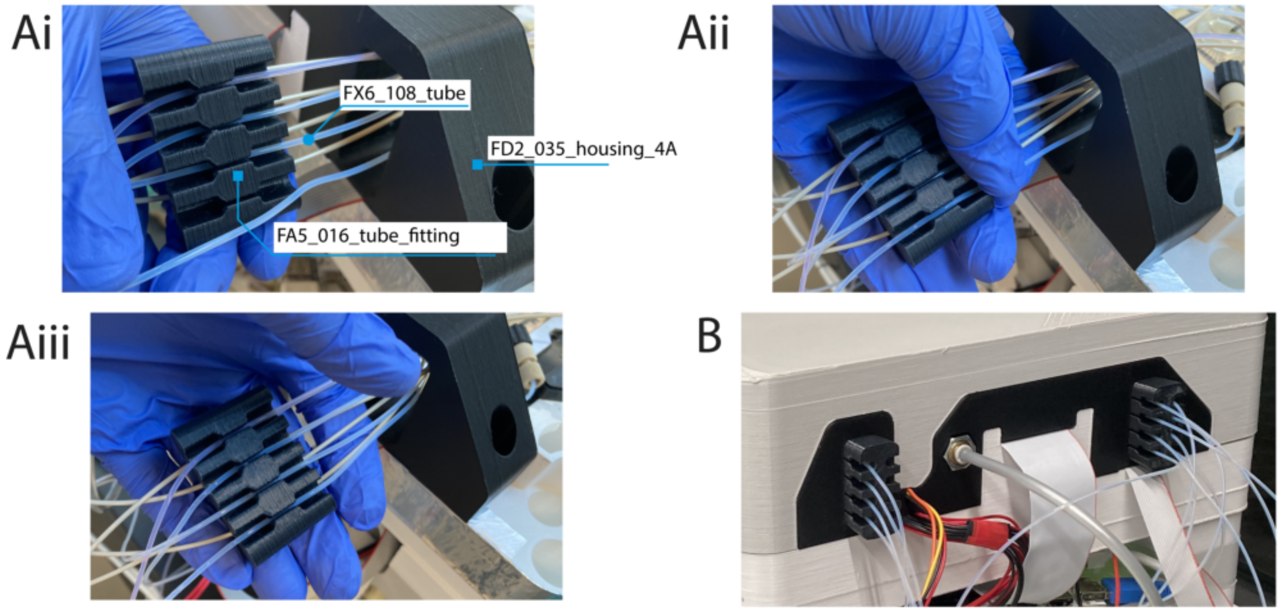

Figure A35 **A**i) Lead all tubes through the gaps in the incubation layer wall. ii) and iii) Bend and clip in the tubes. **B** Push the fittings in. Pay attention to not blocking the tubes, which can happen when pushing the tube fitting in too far.

I Volume measurement

I.1 inkulevel

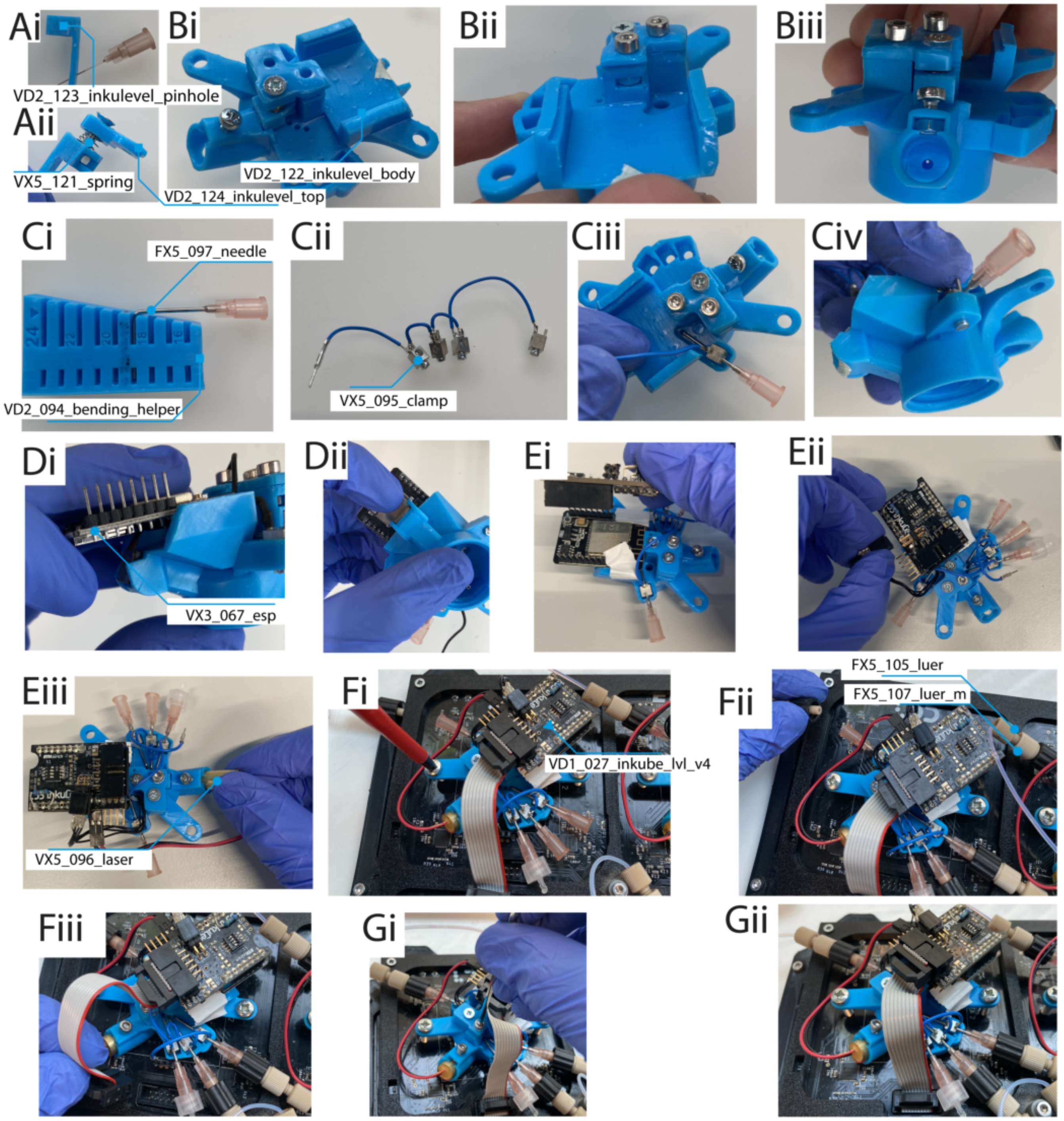

Figure A36 **A**i) Ensure the correct pinhole size by inserting a 0.45 mm syringe needle. ii) Mount the pinhole to the inkulevel top with a spring, a screw, and a M3 hex nut. **B**i) Insert this into the slit in the inkulevel body. ii) and iii) Insert hex nuts to screw the top part into place. **C**i) Bend the needles to the desired length. To insert the needle into the liquid 19 mm are recommend and used throughout this work. ii) Connect the clamps for grounding. iii) Insert the needle through the clamp. Place it on the inkulevel body. iv) Screw it into place. **D**i) Slide the ESP board in from the side. ii) attach the camera to the body. It should nicely click in. **E**i) Mount the shield. ii) Insert the temperature sensor and connect it according to the label on the shield. iii) Insert and connect the laser. **F**i) Screw inkulevel into place on top of the MEA. Insert the clamp cable to the reference connection on the top board. When removing the top board to exchange MEAs, these can be kept in place. ii) Connect the liquid tubes. iii) Make the bottom board connection. **G**i) Adjust the laser height until the reflection appears on the camera sensor. ii) Final setup.

J Environment control

J.1 Inkusense board

Use the sensor board as previously published with[1]. Solder a pin header to the GND, V_{SS}, SDA, and SCL pin and connect as shown in Fig. A24C.

J.2 Reservoir resistive heater

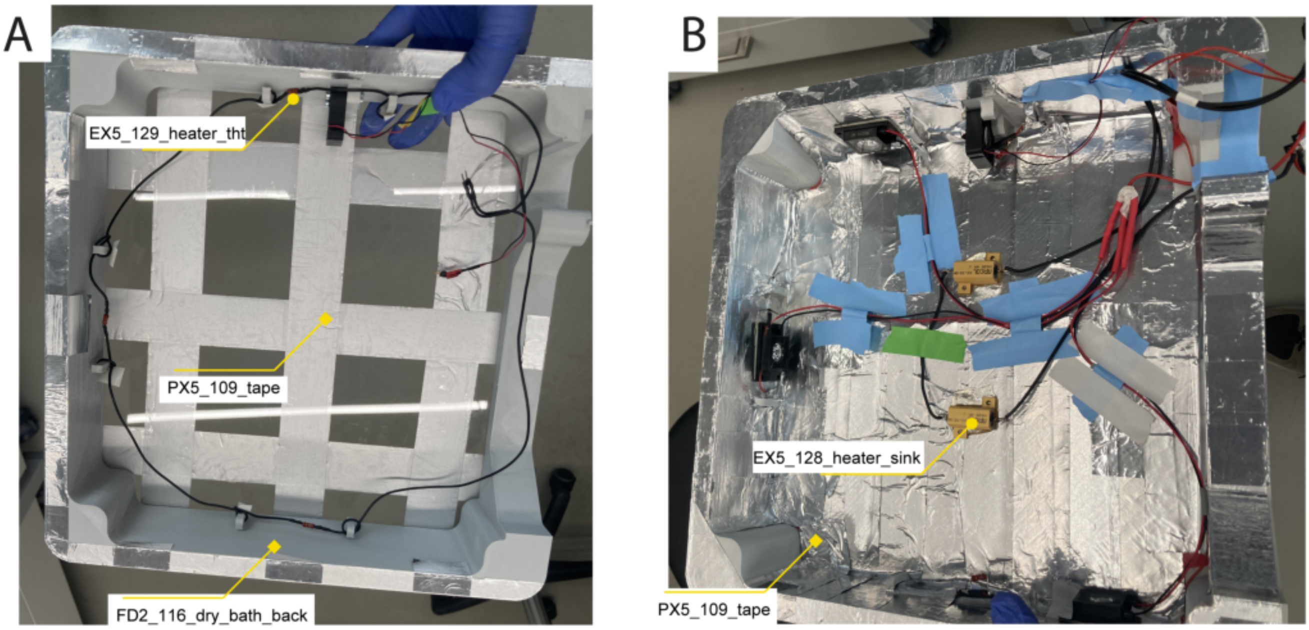

Figure A37 A Connect 3 of the 4 Ω resistors in series. Wrap the cable around the hooks to hold them in place. **B** Additionally, the resistors with heat sinks can be placed in the center and glued with epoxy.

J.3 CO_2 valve assembly

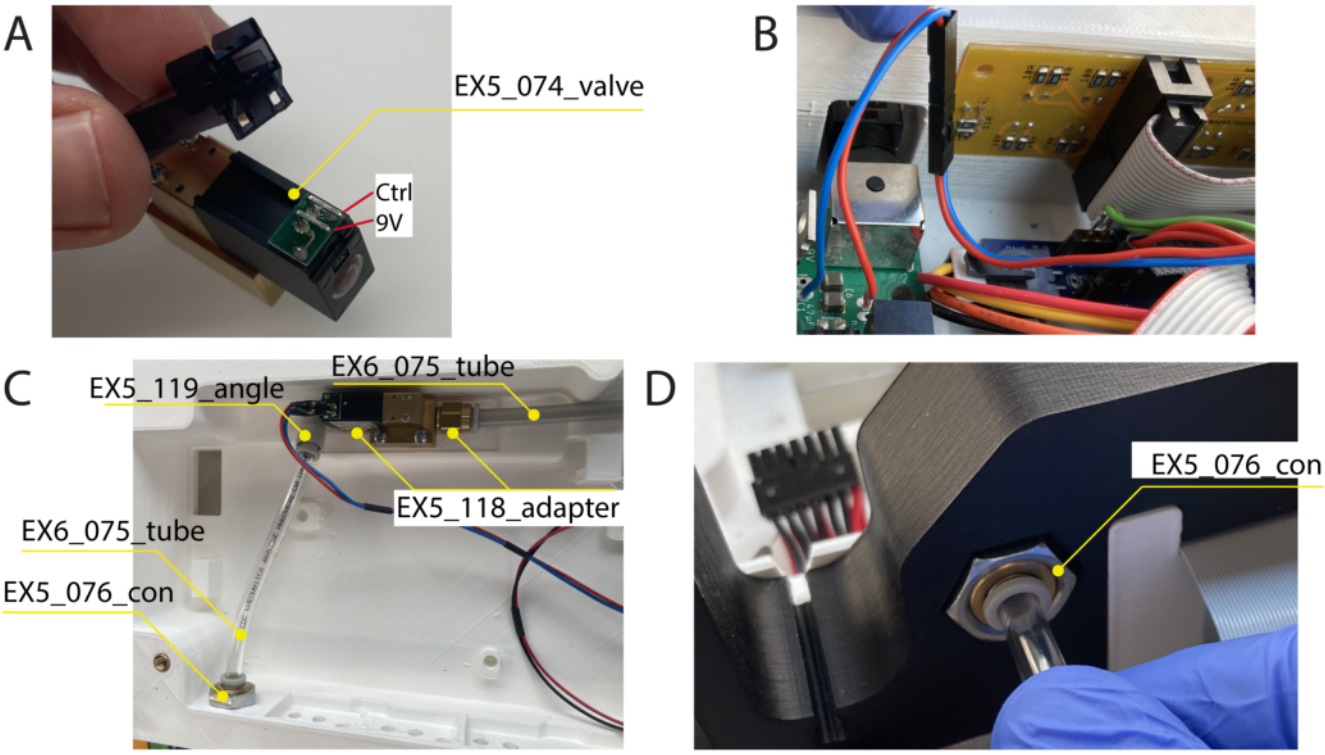

Figure A38 **A** Remove the clip on top of the connector pins of the valve. **B** Connect the valve to 9 V and the CO_2 control pin on the power board. **C** Screw in the tube adapters on both ends. Attach the angle to the input end. Mount the valve with the screws as shown in Fig. A10. Cut a piece of tube and connect the input with the wall connector in the electronics layer. Cut a second longer piece, connect it to the output, and lead it out the electronics layer. **D** Connect the output tube to the wall connector in the incubation layer.

J.4 Humidity heater

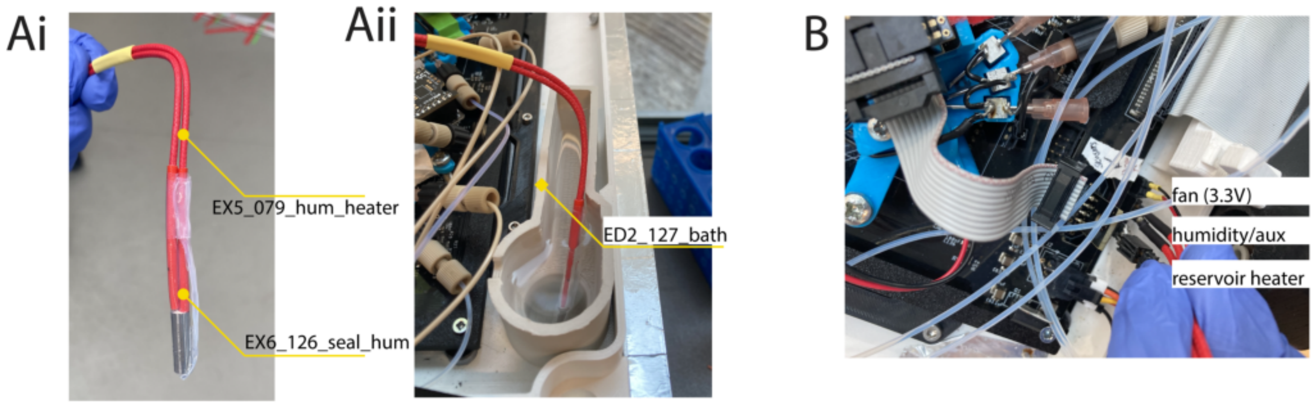

Figure A39 Ai) Cut the cable of the heating cartridge to about 25 cm. Solder a pin header to the cable ends. Cut 2 pieces of PTFE shrink tube. Push the first piece half over the cartridge, bend it and hold it in place with the second piece. Use a heat gun at 400 °C to shrink the tube. ii) Fill the bath and insert the heater. **B** Connect the heater to the base board.

J.5 Fan for stable control

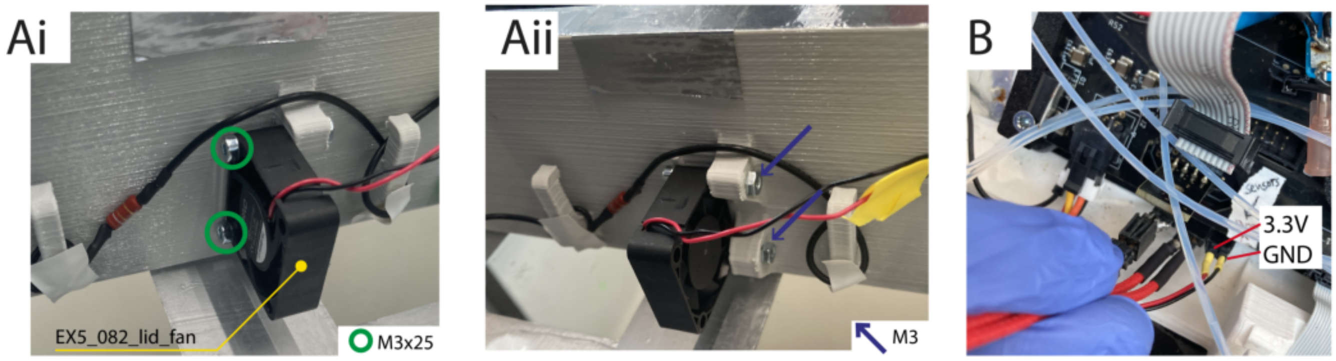

Figure A40 Ai) and ii) Use 2 screws to mount the fan. **B** Connect the fan to the base board.

K Equipment and unspecified components

- soldering iron and reflow oven
- LCD resin printer (Sonic Mini 4K, PHROZEN TECH CO., LTD., Hsinchu City, Taiwan), resins: PowerResinEu SG (Surgical Guide) (PowerResinsEU, Istanbul, Turkey), Phrozen Aqua Resin 4K PHR-RS1000AQG4K (PHROZEN TECH CO., LTD., Hsinchu City, Taiwan)
- extrusion printer for large parts (Ender 5 Plus, Shenzhen Creality 3D Technology Co Ltd, Shenzhen, China)
- extrusion printer for finer parts (Prusa i3 MK3S, Prusa Research a.s., Prague, Czech Republic)
- screwdrivers
- shrinking tube
- Crimping tool
- UART FTDI programmer or ESP32-CAM-MB Micro USB Programmer with CH340G Serial Chip (for flashing ESP32)
- screws as described layer figures
- M1.6, M2.5, M3 hex nuts
- M3, M4, M5 thread inserts

- cables and crimp contacts
- SD card for SoC

L Installation and operation

This section contains information on how to flash the embedded devices, enable USB and network communication of the SoC and the PC, and install and run the software.

L.1 SoC

The compiled SoC code can be found in the inkube git under `ArtyZ7_SoC/BOOT.bin`. Move the file to a micro SD card and insert the card into the Arty Z7 board. Set the jumpers: JP4 to SD and JP5 to REG.

L.2 PC connection

In order to enable the readout from the SoC, the USB and network communication with the PC have to be established. For this, power the SoC and follow the instructions in this section. Initially, the 2 RGB LEDs of the SoC should light up in blue. They indicate the status of the ethernet and USB connection.

L.2.1 Ethernet

Connect the ethernet cable to the PC. Adapt the settings of the port as shown in Fig. A41. It might be necessary to reset the SoC afterwards. Once communication has been established, one blue LED turns off. Proceed with the next section.

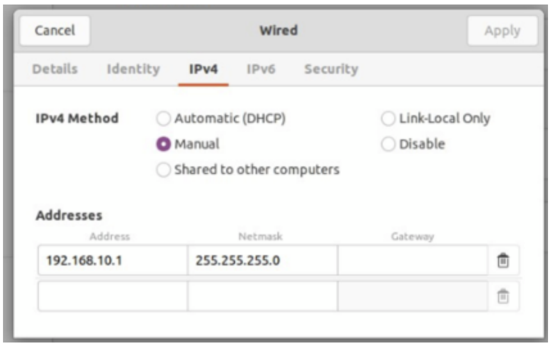

Figure A41 Ethernet settings to connect to FPGA.

L.2.2 USB

Connect the USB cable to a USB port of the PC. Follow the instructions below to allow communication of the PC with the SoC. The instructions can also be found in `USB_communication.py`.

- Open a terminal and execute the command: `lsusb`
- The result should contain a line with: `Bus 00x Device 00y: ID 33ff:1234 Inkube This is the downlink USB connection of Inkube` with `00x` being the bus ID and `00y` the device ID.
- Show the permissions by executing the command: `ls -l /dev/bus/usb/00x/00y`
- if no access rights add the rules file `01-inkube_usb_permissions.rules` with content: `SUBSYSTEM=="usb", ATTR\{idVendor\}=="33ff",ATTR\{idProduct\}=="1234",MODE="0666",GROUP="plugdev"` to `/etc/udev/rules.d` by executing the command: `sudonano/etc/udev/rules.d/01-inkube_usb_permissions.rules`
- you might have to reboot the PC for the changes to take effect
- once the communication has been established the second blue LED should turn off
- as a sanity check you can execute the `USB_communication.py` file, this should make the LEDs on the FPGA blink (LD0 – LD3)

L.3 System startup

In order to use the GUI and control class interface, the Python readout process has to be running. For this follow the steps below.

- clone the inkube git repository

- install pip requirements
- build cython code for spike readout by executing in the directory `Readout_python`: `pythonsetup_filter.pybuild_ext--inplace`
- Execute `pythonReadout_python/main.py` to start readout and open the GUI. You can now use the example jupyter notebooks to change the environmental parameters, use the closed loop stimulation, and use the fluidic system.

L.4 Inkulevel

To receive feedback about the volume inside the MEA, inkulevel has to be flashed. For this one of the options listed in can be used together with the arduino IDE . With the FTDI chip, the GPIO 0 has to be pulled low. If the device is correctly flashed, the red LED should be constantly on once the board is powered. In case of a blinking red LED, an error occurred upon booting.

It is recommended to start by adjusting the laser while the image is streamed via bluetooth with the `bt_all_script_integral.py` script, which can be found in the inkube git under `inkulevel/Python_bt`. Be aware that this requires additional libraries. Then the settings can be tweaked for optimal detection. Standard parameters and values can be found in the example jupyter notebook `Experiments/02_Fluidics_Environment_Control.ipynb` in the inkube git.

References

[1] Duru, J., Maurer, B., Ruff, T., Vulić, K., Hengsteler, J., Girardin, S., Vörös, J., Ihle, S.J., 2024. A modular and flexible open source cell incubator system for mobile and stationary use. HardwareX 20, e00571.

[2] Espressif Systems, 2023. Camerawebserver. https://github.com/espressif/arduino-esp32/tree/09a6770320b75c219053aa19d630afe1a7c61147/libraries/ESP32/examples/Camera/CameraWebServer. Accessed: 2024-11-13.

[3] Garma, L.D., Matino, L., Melle, G., Moia, F., De Angelis, F., Santoro, F., Dipalo, M., 2019. Cost-effective and multifunctional acquisition system for in vitro electrophysiological investigations with multi-electrode arrays. PloS one 14, e0214017.
