# Supplementary material for: Inkube: an all-in-one solution for neuron culturing, electrophysiology, and fluidic exchange
Source: Lab Chip. 2026 Mar 2;26(6):2074–89. doi: 10.1039/d5lc00971e (PMC12951282; doi:10.1039/d5lc00971e)
Supplement: LC-026-D5LC00971E-s002 [file LC-026-D5LC00971E-s002.pdf]

Supplementary Information (SI) for Lab on a Chip.
This journal is © The Royal Society of Chemistry 2026

Inkube: An all-in-one solution for neuron culturing, electrophysiology, and fluidic exchange

Benedikt Maurer[†, a], Selina Fassbind[a], Tobias Ruff[a], Jens Duru[a], Giusy Spacone[a], Theo Rodde[a], János Vörös[a], and Stephan J. Ihle[†, a, b, c, ‡]

Supplementary Information

[a]Laboratory of Biosensors and Bioelectronics, Institute for Biomedical Engineering, University and ETH Zurich, Gloriastrasse 37/39, Zurich, 8092, Switzerland
[b]Department of Neurobiology, University of Chicago, 951 E 58th St, Chicago, 60637, IL, USA
[c]Department of Physics, University of Chicago, 929 E 57th St, Chicago, 60637, IL, USA
[†]These authors contributed equally to this work
[‡]Corresponding author: ihles@uchicago.edu

A Software

An overview of the software architecture of inkube is provided in Fig. S1. It can be subdivided into 3 main parts: the software performing the low-level hardware communication on the SoC, the user interaction and electrophysiology analysis on the host PC, and the point detection on inkulevel. All parts and interfaces will be described in the following sections.

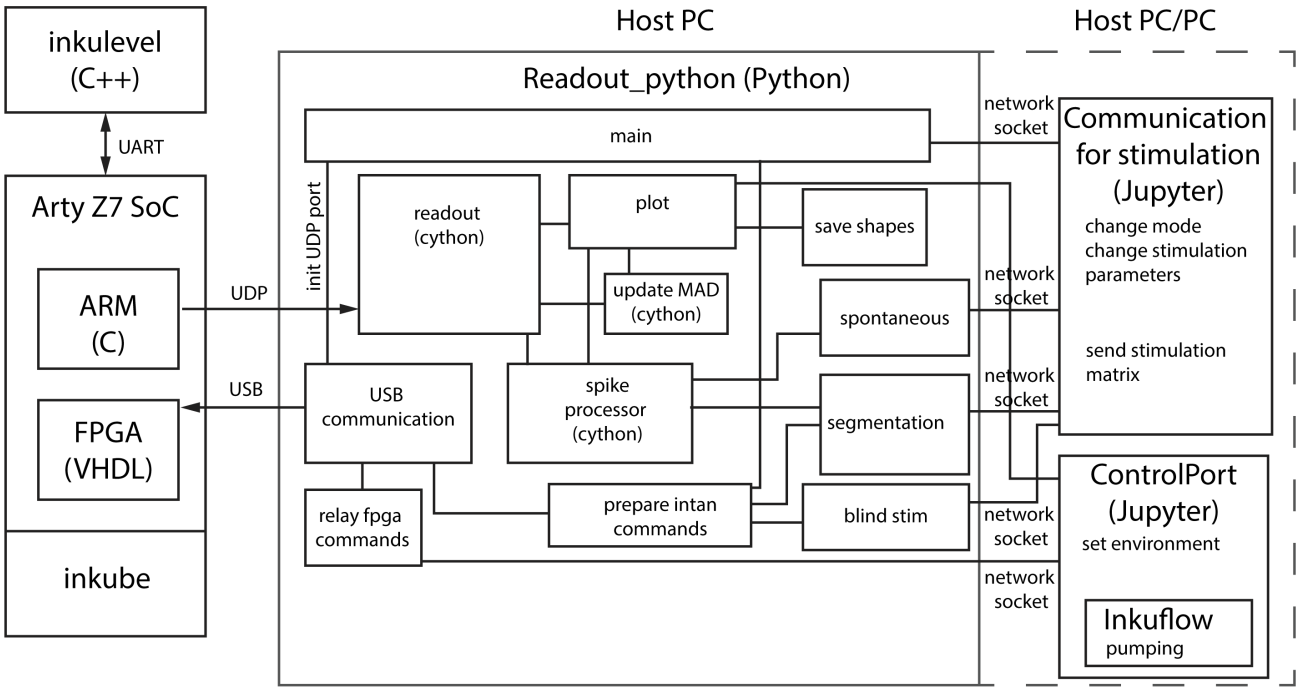

Figure S1 Overview block diagram of the software for the SoC, the host PC, and inkulevel.

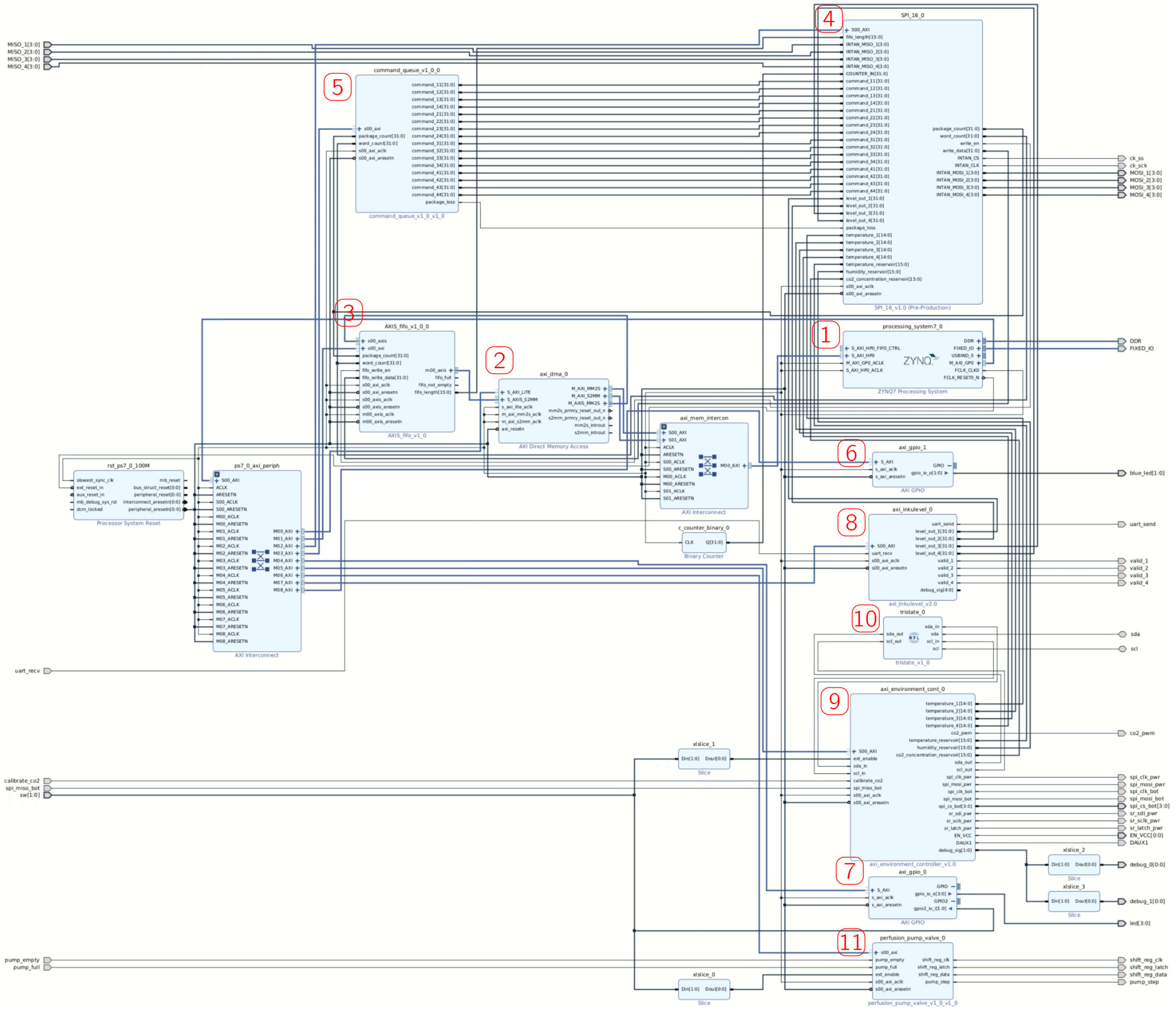

Figure S2 Block diagram of the SoC. **(1)** Hard core of the system. **(2)** Direct memory access to send data via the UDP protocol to the PC. **(3)** FIFO that is transforming the data from the INTAN chips into an AXI4-Stream. **(4)** Interface to control the INTAN ICs. **(5)** Interface to control the commands to be used for the INTAN ICs. **(6)** Interface to control the blue channels of the 2 Arty Z7 RGB LEDs. **(7)** Interface to interact with the switches and the mono-color LEDs of the Arty Z7 board. **(8)** Interface used to communicate with the 4 inkulevels. **(9)** Interface to measure and control the environment parameter. **(10)** Tristate Interface to implement allow for a high-impedance input for the I^{2}C interface. **(11)** Interface for the perfusion system.

A.1 SoC

inkube is built around the Arty Z7-20 (Digilent, Pullman, WA, USA), which contains a ZYNQ-7000 SoC (XC7Z020-1CLG400C, Xilinx, San Jose, CA). The SoC is programmed in VHDL and C. The block diagram of the SoC is given in Fig. S2.

The CPU of the ZYNQ SoC (see Fig. S2) is running at 650 MHz, while the DDR is running at 525 MHz. The Programmable Logic fabric clock runs at 100 MHz. The system uses two different types of Advanced eXtensible Interfaces (AXIs) for inkube. Most of the interfaces use AXI4-Lite (AXI4L), except for the datastream sent from inkube to the PC, which uses an AXI4-Stream (AXI4S). The address ranges of different AXIs are given in Fig. S3.

Name	Interface	Slave Segment	Master Base Address	Range	Master High Address
Network 0					
/axi_dma_0					
/axi_dma_0/Data_MM2S (32 address bits : 4G)					
/processing_system7_0/S_AXI_HP0	S_AXI_HP0	HP0_DDR_LOWOCM	0x0000_0000	512M	0x1FFF_FFFF
/axi_dma_0/Data_S2MM (32 address bits : 4G)					
/processing_system7_0/S_AXI_HP0	S_AXI_HP0	HP0_DDR_LOWOCM	0x0000_0000	512M	0x1FFF_FFFF
Network 1					
/processing_system7_0					
/processing_system7_0/Data (32 address bits : 0x40000000 [1G])					
/axi_dma_0/S_AXI_LITE	S_AXI_LITE	Reg	0x4040_0000	64K	0x4040_FFFF
/axi_environment_cont_0/S00_AXI	S00_AXI	S00_AXI_reg	0x43C3_0000	64K	0x43C3_FFFF
/axi_gpio_0/S_AXI	S_AXI	Reg	0x4120_0000	64K	0x4120_FFFF
/axi_gpio_1/S_AXI	S_AXI	Reg	0x4121_0000	64K	0x4121_FFFF
/axi_inkulevel_0/S00_AXI	S00_AXI	S00_AXI_reg	0x43C5_0000	64K	0x43C5_FFFF
/AXIS_fifo_v1_0_0/s00_axi	s00_axi	reg0	0x43C0_0000	64K	0x43C0_FFFF
/command_queue_v1_0_0/s00_axi	s00_axi	reg0	0x43C2_0000	64K	0x43C2_FFFF
/perfusion_pump_valve_0/s00_axi	s00_axi	reg0	0x43C4_0000	64K	0x43C4_FFFF
/SPI_16_0/S00_AXI	S00_AXI	S00_AXI_reg	0x43C1_0000	64K	0x43C1_FFFF

Figure S3 Address ranges of different AXIs of inkube. While large 64K address ranges are reserved for all AXI4Ls, not all of these addresses are actually used.

The AXI4S is controlled through a direct memory access (DMA, see Fig. S22), which packages data directly into UDP data. The UDP/IP headers and checksum are created by the processor of the ZYNQ SoC. For that, the IPv4 (192.160.10.10) and MAC (00:0a:35:01:02:03) address of inkube are hard-coded but can be changed in the C code. Similarly, for simplicity the PC connecting with inkube must have the IPv4 address of 192.168.10.1. In order to connect to inkube, an ARP request with these IP addresses must be sent to inkube. Inkube is capable of responding to ARP requests. However, ICMP is not implemented for inkube and cannot be used. Furthermore, to build up a connection with inkube, it also must be connected to the PC. Both the Ethernet and USB connection must be correctly set up for inkube to be capable of communicating with the PC to interact with inkube. The AXI4S is controlled by AXIS_fifo (see Fig. S23, which is containing a FIFO of width 32 bits and a depth of 32,768 entries. The status of the FIFO can be read out with an additional AXI4L (address range: 43C0XXXXh). The FIFO predominantly receives its data from the SPI_16 interface.

The SPI_16 interface (see Fig. S24 is communicating directly with the INTAN ICs through a Serial Peripheral Interface (SPI) with a shared chip select and clock signal, but 16 independent data channels; one for each IC. The SPI is running at 12.5 MHz. The whole interface is controlled by an AXI4L (address range: 43C1XXXXh). This interface also initializes the INTAN ICs at startup and sends data to the AXIS_fifo interface, which is then transformed into UDP packages through the DMA. Each UDP package also contains the parameters of the inkube environment such as CO_2 concentration, temperatures, humidity, and inkulevel values, which are also sent to the FIFO. Finally, this interface is also sending commands to the INTAN ICs. With each measurement cycle, where all 16 electrodes of an INTAN are measured and read out, 4 commands can be sent to each INTAN IC in addition. However, inkube currently only adds the INTAN responses of the first two commands to the FIFO. However, all commands are fed into the FIFO as a read back and to allow for frame perfect timing reconstructions. The commands are controlled by the command_queue interface. The SPI_16 interface is creating global counters “word_count” and “package_count”. The package_count counter is counting once for every measurement frame (32 bit, cycles approx. every 90 sec), while the word_count counter is used as a synchronization tool within a measurement frame.

The command_queue interface (see Fig. S25 is responsible for sending out the commands to the SPI_16 interface at the requested time points. The data is sent to the interface through a FIFO of a width of 2080 bit and a depth of 32. Each entry is a set of 64 commands (4 for each INTAN IC). These are the 4 commands used for a single measurement cycle. Since each command has 32 bit, this requires a total of 2048 bit. The remaining 32 bit decide on the time when the command shall be executed, which is based on the package_count counter. Since this counter cycles every 90 s, the commands need to be added at the appropriate time to hit the correct cycle. In addition, only the oldest element

in the FIFO is considered at any time. As a consequence, commands need to be added in the order that they need to be executed. The FIFO itself does not sort the commands by time. The commands are sent to the command_queue interface through an AXI4L (address range: 43C2XXXXh) from the processor, which in turn received commands from the PC through the USB connection.

inkube is using the switches and LEDs that are connected to the ZYNQ on the ARTY-Z7. The blue channels of the two RGB LEDs of ARTY-Z7 are controlled by the axi_gpio_1 interface (see Fig. S26), controlled by an AXI4L (address range: 4121XXXXh). If the two blue channels are switched off, this means that inkube has received data through the USB connection. In addition, the axi_gpio_0 interface (see Fig. S27) is connected to the two switches and the 4 mono-color LEDs. The 4 LEDs encode (from 0 to 2) the state of the left switch, whether the command queue is full, and whether the command queue is not empty. Finally, the 3rd LED flashes every time an Ethernet package is received. The two switches are enabling environment control and are discussed further in later paragraphs.

The 4 inkulevels communicate with the ZYNQ SoC through the axi_inkulevel interface (see Fig. S28). It is controlled with an AXI4L (address range: 43C5XXXXh). Specifically, a daisy-chain has been implemented through a universal asynchronous receiver-transmitter (UART) protocol. The measured level values are sent to the SPI_16 interface, while the validity of the measurement of the 4 levels is visually described by the color of the red and green channels of the 2 RGB LEDs.

To control the MEA temperatures, as well as the temperature, CO_2 concentration, and humidity levels of the reservoir, inkube is using the axi_environment_cont interface (see Fig. S29). This interface is controlled with an AXI4L (address range: 43C3XXXXh) and controls an adjustable PI controller. The controller needs to be enabled by the Arty Z7 switch 1. This way, the environment control can be easily switched off while the rest of inkube continues running. In addition, the controllers can be switched on and off through the AXI4L. The axi_environment_cont interface is communicating with the temperature, humidity and CO_2 sensors as well as the environment actuators. For that, both an I^2C interface and SPI has been implemented. An I^2C interface is requiring a tristate input, which is provided by the tristate interface (see Fig. S210).

The perfusion system is controlled through the perfusion_pump_valve interface (see Fig. S211). An AXI4L (address range: 43C4XXXXh) controls this interface, too. The controller needs to be enabled by the Arty Z7 switch 0. Both the syringe pump and the valves of the liquid multiplexers are controlled through shift registers, except for the output signal, which controls the step count of the pump. The interface also receives end-of-range information from two switches integrated into the syringe pump.

A.2 Python

The Python software can be divided into the readout process and 2 classes for interacting with the process, for example within a jupyter notebook. The readout process is started by executing `main.py`. This will establish communication with the SoC via USB and UDP. Subsequently, the child processes are started. The inkube software can run in 4 different modes, which can be set through the `ControlPort` interface.

0 idle: standard mode of operation, no spike times are recorded and no stimulation is executed

1 spontaneous: mode for spontaneous recording, spike times are continuously recorded and sent out in milliseconds since package ID reset in chunks of up to 1.5 s to the `Communication` instance

2 closed-loop stimulation: mode for stimulating the network and record spikes for a pre-defined period after stimulation as latency after recording start, spike times are sent to the `Communication` instance and a new stimulus is received. Stimulation can be applied at up to 8 different timepoints before recording start.

3 blind stimulation: mode for only stimulation, no spike times are recorded. Stimulation pulses are sent in chunks, which circumvents the limit of 8 timepoints

`readout` The data stream from the SoC arrives at a frequency of 17,361 Hz. One data frame consists of the package ID, received commands counter, status information, sensor data, and the data of the electrophysiology chip (see Supplementary Data 02_Code/03_Additional_Code_Documentation/package_format.ods). It is received through the `socket` class in the process `readout`, which is written in Cython. The package ID and the counter for the received commands are copied into shared variables. The status and sensor data is copied from the frame into shared arrays with the plot process. The voltage traces are processed in a parallelized for loop. There, the filtering is applied and spike detection is performed. The filtered data, the data for thresholding, and the spike times are then copied into shared arrays with the plot process. Additionally, there is the option to extract the spike waveforms and send them to the plot process. The detected spikes are also put on a pipe to the spike processor.

`update MAD` The threshold is computed on the data shared with the plotting process. Furthermore, the noise levels are computed here. Update rate and sampling can be adapted in the process call. The threshold update can be enabled or disabled through a checkbox in the GUI.

`plot` The plot process runs multiple threads that update the data for plotting. In the same thread, temperature, level, and spike shape data can be saved in chunks. The main thread hosts the application with the GUI, based on the PyQt6 library. The GUI is organized into different tabs, which can be selected on the top left.

The first tab is the signals tabs, where the voltage traces of 60 electrodes are plotted. In the standard settings the last 500 ms are shown in oscilloscope mode at an updated rate of 4 Hz. When spikes are detected, a triangle is plotted at $y = 0$ on the voltage trace. An example plot is shown in Fig. S4.

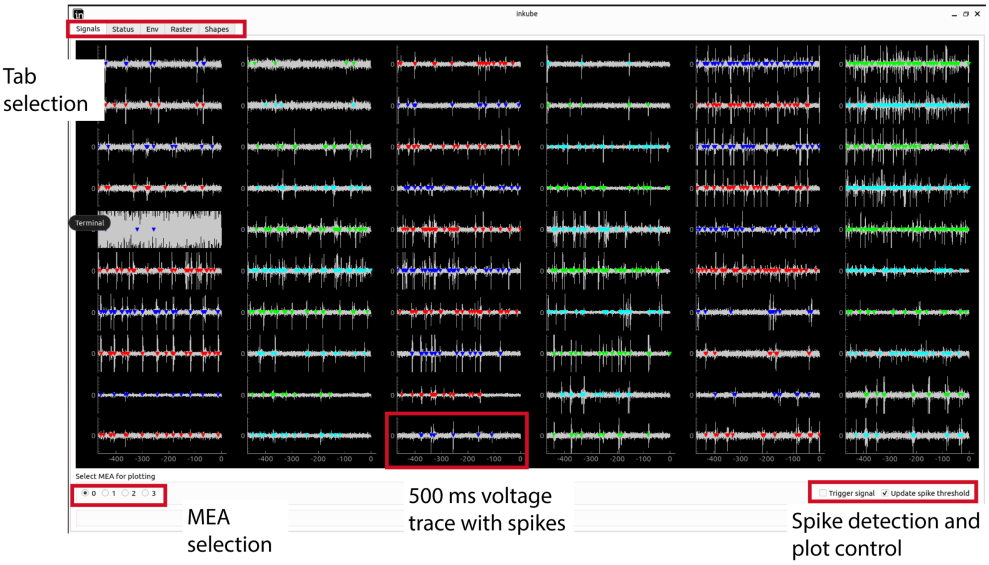

Figure S4 Signals tab of the inkube GUI showing the past 500 ms of the voltage signals of all 60 electrodes of an MEA. Detected spikes are marked with triangles. The y-axis is plotted in µV.

In the status tab depicted in Fig. S5, selected status data is shown over the past 500 ms. The first plot always shows the incrementing package ID. Additional information can be extracted from the aforementioned UDP package documentation.

The third tab shown in Fig. S6 provides information about the culture environment, the medium volume, and the noise levels of the electrophysiology data. In the update thread of this tab, the sensor data of the culture environment and medium volume can be saved in chunks of roughly 5 min. Additionally, the data can be shared utilizing a queue accessible through a network interface with a `ControlPort` instance to enable feedbacked control of all parameters.

The raster tab can be seen in Fig. S7. In case of stimulation, the post stimulus time histogram is shown for a single network. Spikes on the different electrodes are depicted in the respective color as a single dot at the measured latency in milliseconds after the recording start. The applied stimulation up to 10 ms before the recording start is plotted at a negative latency. The y-axis comprises 90 s of data and is then reset.

The spike shapes tab depicted in Fig. S8 shows cutout spike waveforms of roughly 2 ms of a full MEA. The shapes can be saved together with the respective package ID to enable spike sorting.

`spike processor` The spike processor process receives the spike time and electrode through a pipe. It can operate in 3 different modes. For mode 0 and 3, spikes are read from the pipe interface and discarded. For mode 1, all spikes are sent to the spontaneous readout process. For mode 2, spike times are compared to the recording start and discarded, if they lie outside the recording window (usually 20 ms after stimulation). Once the first spike past the window is detected or an external segmentation signal indicates that the window is over, all spike times are translated into latencies after window start and passed to the segmentation process.

`spontaneous` The spontaneous thread receives spike times from the `spike processor` and shares them through the network interface with the `Communication for stimulation`. The spontaneous thread utilizes the `ElectrodeMapping` class, which translates the channel IDs into network and MEA position. The spike times are shared as tuples for each electrode in a numpy array of dimension $network \times electrode$.

`segmentation` The segmentation process is used for the closed-loop stimulation (mode 2). It divides the recording time, base don the package ID, into periods of usually 250 ms. At the start of a period, spike times are recorded during the recording window (usually 5 to 25 ms). Afterwards, the recorded spikes are organized by network and electrode, similar to the spontaneous thread, and translated into latencies with respect to the window start. The spike matrix is subsequently sent out to the `Communication for stimulation` instance together with information about the period: a flag whether a stimulus has been received and executed for this period, the executed stimulation matrix, and the ID of the period). The process then waits for a stimulation command from the `Communication for stimulation` instance, up until either a stimulation command is received with the period ID corresponding to the following period

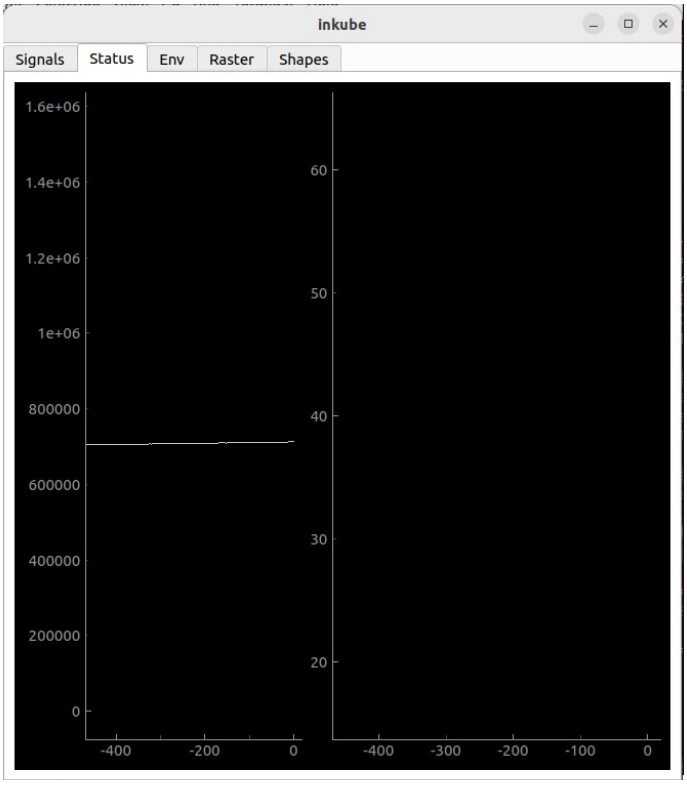

Figure S5 Status tab of the inkube GUI. Different status words can be plotted here over the past 500 ms. The left plot shows the linearly increasing package ID.

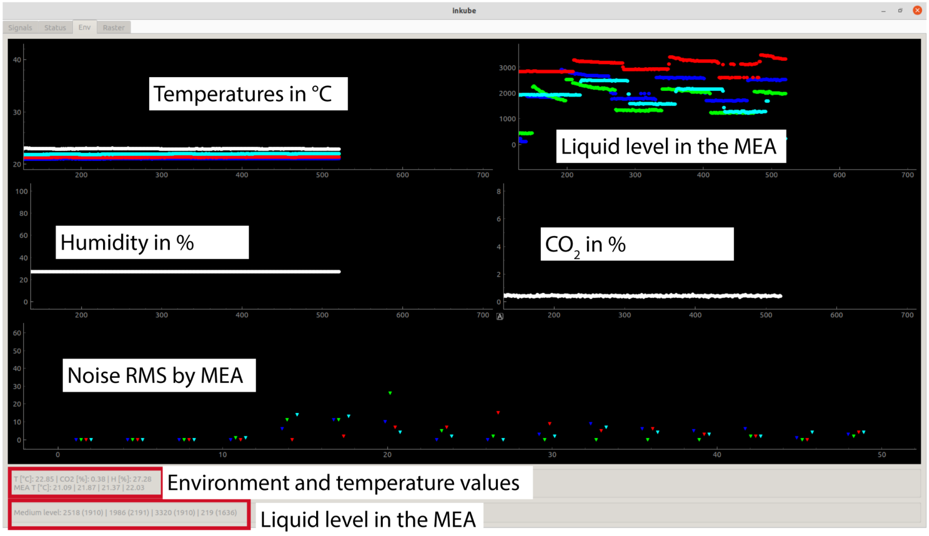

Figure S6 Env tab of the inkube GUI. The top 4 plots show data of the culture environment for up to 1 h. The x-axis unit is time in minutes. The top left plot shows the temperature of the reservoir in white and the 4 MEAs in the MEA colors (blue, green, red, cyan). The top right plot shows the inkulevel values for the 4 MEAs. The bottom left plot shows the relative humidity in the reservoir in % V/V and the bottom right plot show the CO_2 concentration in % V/V. All current values are also shown in the text fields in the bottom. The bottom plot shows the RMS noise for each MEA in the respective color with the bins in µV on the x-axis and the electrode count on the y-axis.

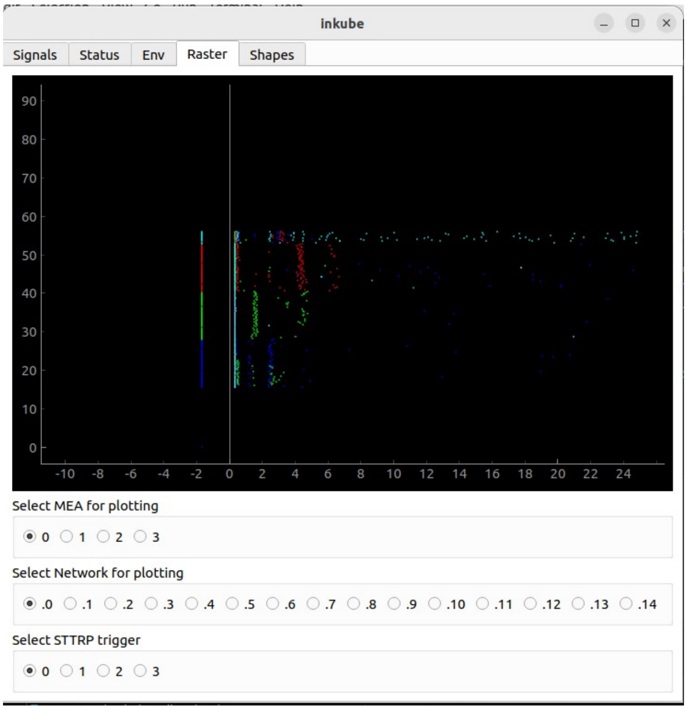

Figure S7 Raster tab of the inkube GUI. During stimulation (mode 2), the post-stimulus raster plots are shown, with the applied pattern up until 10 ms before recording start

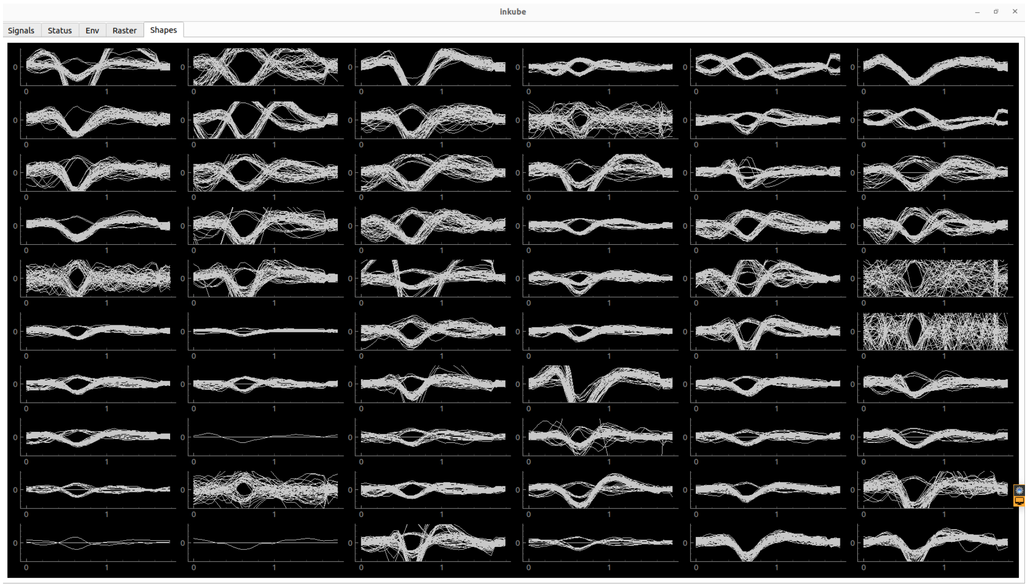

Figure S8 Shapes tab of the inkube GUI. When a spike is detected, about 2 ms are stored of the voltage signal and plotted on top of each other.

or the maximum wait time is over. If a valid stimulation command has been received, the stimulation is sent to the `prepare intan commands` process. The stimulation time points are received as relative values in delay in samples before recording start and translated into the corresponding package IDs for the commands. Once the stimulation is forwarded, the period ID is incremented and the corresponding updated recording window times are passed to the `spike processor`.

`blind stimulation` The blind stimulation process is an adapted version of the segmentation process. Here, no spikes are recorded and therefore no synchronization with the spike processor has to be achieved. The information sent to the `Communication for stimulation` instance is reduced to the period ID, stimulation flag, and stimulation matrix. Stimulation commands are however not executed in the current period before recording start but in the following period. In case the stimulation has to be performed at more than 8 different timepoints, the stimulation matrix is chunked and sent dynamically to the `prepare intan commands` process.

`prepare intan commands` This process receives the electrode IDs and timepoints as package IDs where a stimulation pulse should be sent. Additionally, a flag is received whether this is a first or last pulse of a stimulation sequence. For the first and last pulse, commands are sent to all chips for fast settle and high-pass filter pole shift in order to reduce the stimulation artifact. From the electrode IDs the chip ID is derived. The stimulation pulses are sent to the respective electrodes. An active discharge is performed after the pulse on all electrodes of a whole chip of which at least one electrode has been stimulated. The commands are then passed to the `USB communication` process.

`relay fpga commands` This process receives commands for the pump, valve multiplexers, and environment set values from a `ControlPort` instance and sends them to the `USB communication` process.

`USB communication` This process receives commands in the final format together with an ID indicating the command type and sends them to the SoC via USB, optionally with a handshake. The handshake is performed by including the package ID into the first byte of the command after the package preamble and synchronizing it withg the received packages counter in the UDP package sent by the SoC. Commands can be of following types:

1 send port for UDP communication, no handshake

2 send intan commands, with receive package id (handshake), directly forwarded to ASIC by the FPGA

3 send fpga commands, with receive package id (handshake), these are register writes directly on the SoC for e.g. pumping

4 send reset command that sets receive package counter to 0, with receive package id (handshake)

`ControlPort` Set values for the environment controller can be set through an instance of the ControlPort class. Furthermore, the sensor data can be received for closed-loop control. Pumping and switching of the valves can be performed through the `Inkuflow` class, of which an instance is a member of the `ControlPort` class. A logging file is created by the instance with the sent commands and timestamps.

`Communication for stimulation` The `Communication for stimulation` instance receives spikes either from spontaneous recording (mode 1) or from the recording window during closed-loop stimulation (mode 2). Stimulation pulses are sent back as a matrix of the format $n \times 3$, where n corresponds to the number of pulses. The columns correspond to the delay of the stimulation pulse in samples before the recording window start, the network ID, and the electrode ID. The ID of the period for execution is derived by incrementing the last received period.

A.3 inkulevel

inkulevel is built around an ESP32 microcontroller, which can be programmed in C++ using the arduino IDE. The code is based on the arduino ESP webserver example: `https://github.com/espressif/arduino-esp32/tree/09a6770320b75c219053aa19d630afe1a7c61147/libraries/ESP32/examples/Camera/CameraWebServer`[1] The main functionality is the detection of the laser reflection on the sensor area. UART communication is used to change the settings of the camera, the point detection, and send out the data. The details for extraction and communication can be extracted from the code documentation.

B System characterization

This section contains additional information that shows the performance of inkube. The limits of the actuators are tested. Additionally, the controller performance is tested by demonstrating the response to a change of the set value. Subsequently, the electrophysiology is covered with showing the spike detection and stimulation.

B.1 Actuator and controller performance

B.1.1 Reservoir temperature

The reservoir heating is tested with 8 Ω parallel to 12 Ω resistive heaters. Additionally, the water bath is heated with a 14.4 Ω heating cartridge to hold the relative humidity between 60 and 70%.

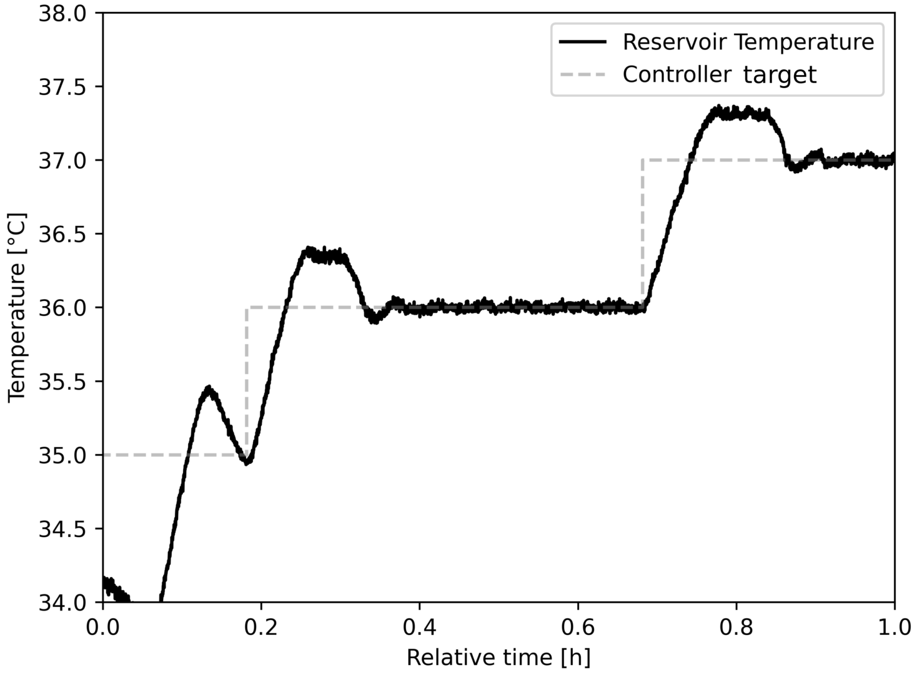

Figure S9 Reservoir temperature when changing the set values (dashed line).

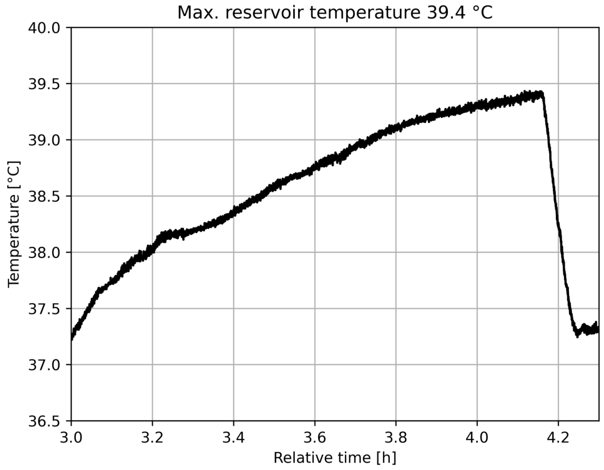

Figure S10 Maximum reservoir temperature achieved with the MEA temperatures at 37 °C and the reservoir set to maximum heating.

B.1.2 Humidity

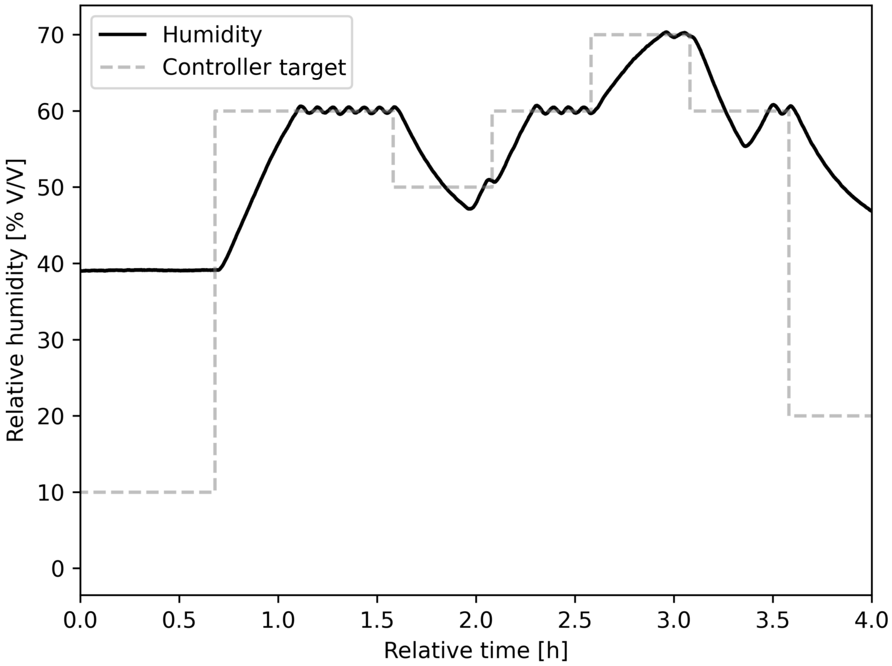

Figure S11 Reservoir relative humidity when changing the set values (dashed line).

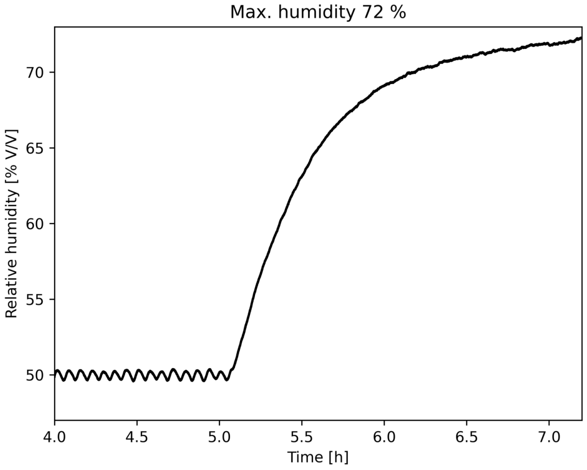

Figure S12 Maximum reservoir relative humidity achieved with the MEA and reservoir temperature at 37 °C and the bath heater set to maximum heating.

B.1.3 CO_2 calibration and transient behavior

The CO_2 valve is supplied with 100% CO_2 at approximately 1.5 bar. The CO_2 sensor has an initialization routine, which is automatically performed at system start-up and a calibration routine, where the current CO_2 measurement is set to 0%. Due to the temperature dependence of the sensor, it is recommended to call the calibration routine once the reservoir has been successfully heated up to 37 °C.

B.1.4 MEA heating and cooling

A class B (according to EN 60 751) PT1000 is used for measuring the liquid temperature, as temperature stability is prioritized over temperature accuracy. Class B implies an absolute accuracy of the impedance of $\pm 0.12\%$, which limits the absolute accuracy of the temperature to roughly 360 mK at 37 °C. Drifts due to self heating are not expected to have a noticeable effect with respect to the quantization error.

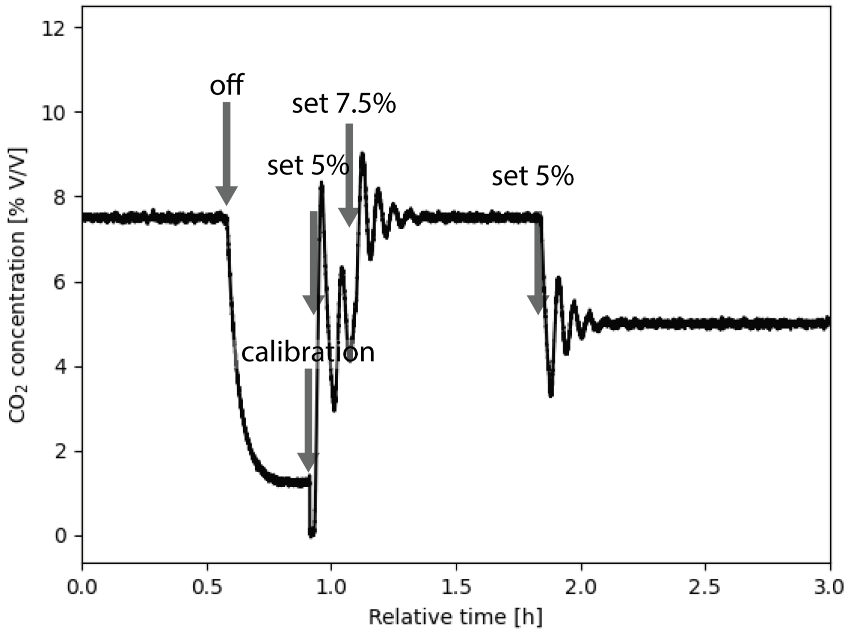

Figure S13 CO_2 value upon turning the valve off, calling the calibration routine, turning it on again at a set value of 5%, increasing the set value to 7.5%, and reducing it to 5% again. Time points are indicated with grey arrows.

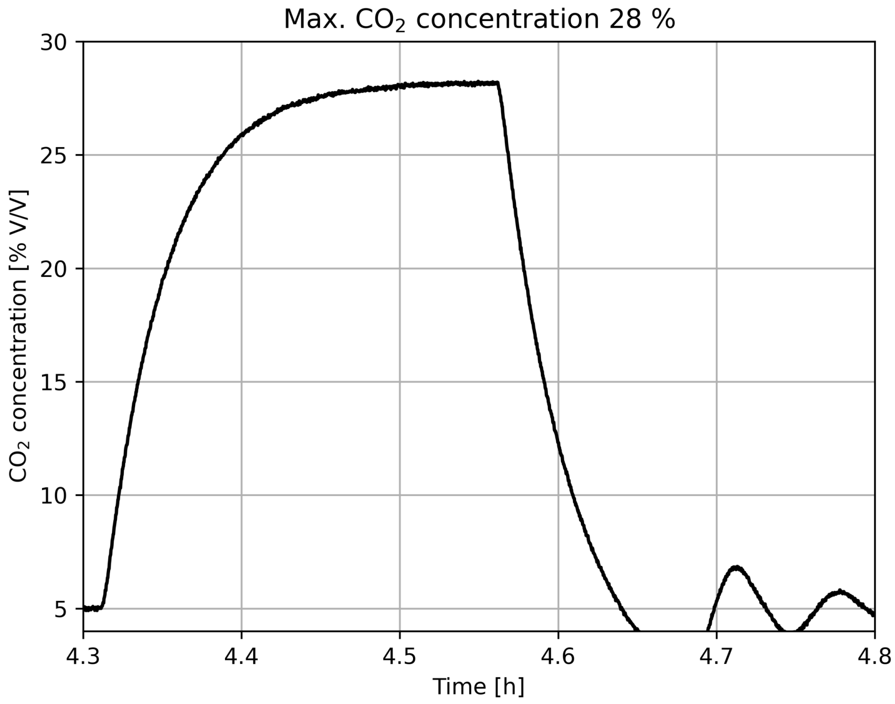

Figure S14 Maximum CO_2 value with the reservoir at 37 °C. The pulse width modulation of opening the valve is limited in order to increase the resolution at smaller values.

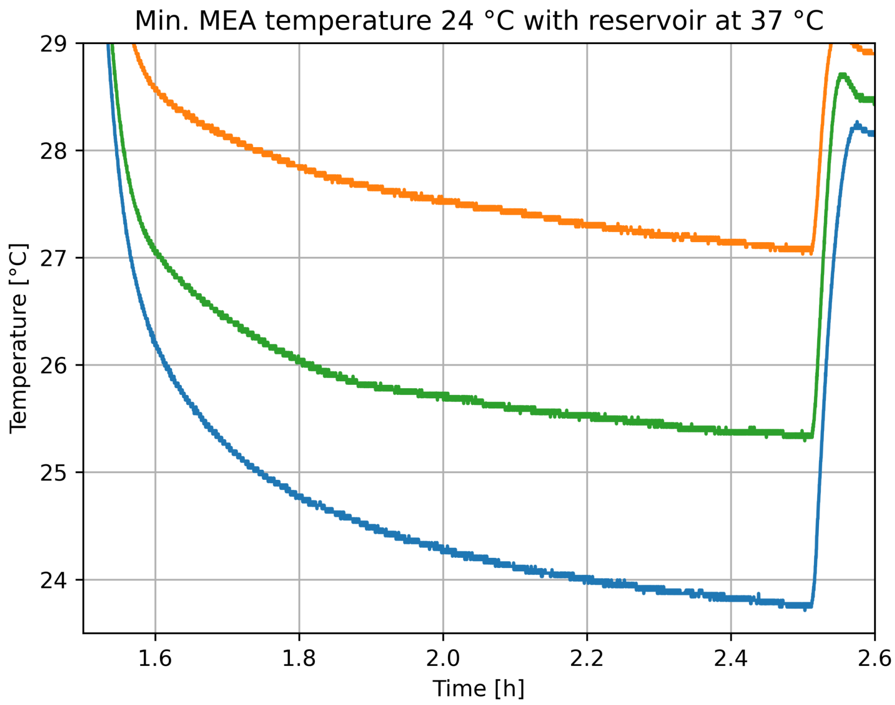

Figure S15 Minimum medium temperature achieved with the reservoir temperature at 37 °C and the thermoelectric devices set to maximum cooling.

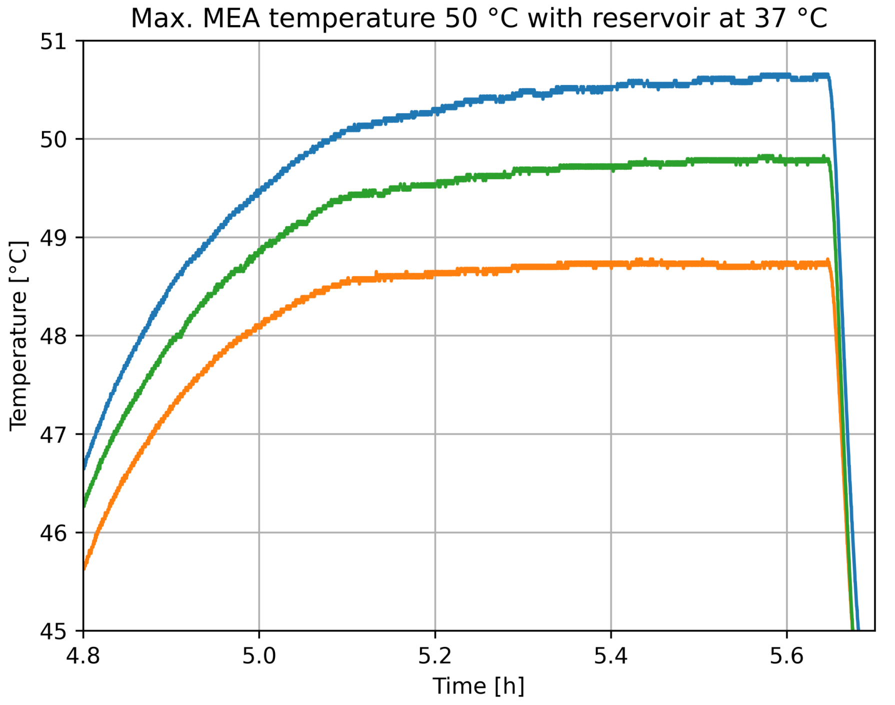

Figure S16 Maximum medium temperature achieved with the reservoir temperature at 37 °C and the thermoelectric devices set to maximum heating.

B.2 Fluidics

The stepper motor based syringe pump is characterized in the following section. Measuring the pumped volume yielded 108.025 μL for 10,000 steps (σ=0.255 μL, n=5). The linearity of the pump across the range of the stepper motor is tested by performing pumping of 100, 500, 1,000, 5,000, and 10,000 steps on the motor, starting at 20,000 and 60,000 steps. The measurement was repeated 3 times with negligible variations between iterations. The influence of the starting position can be neglected. The results can be seen in Fig. S17.

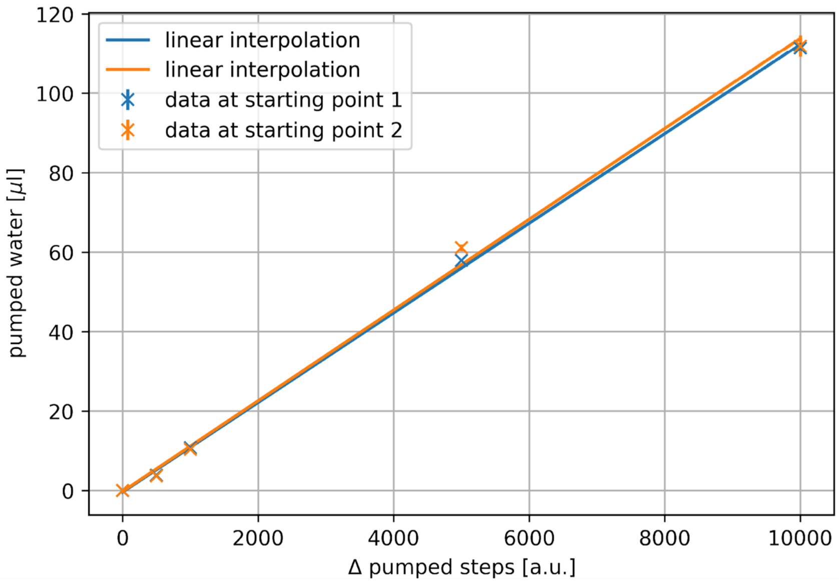

Figure S17 Linearity measurements on the pump, with starting point 1 at 20'000 steps and starting point 2 at 60'000 steps on the motor (n=3).

The hysteresis, the difference between adding and retrieving liquid, of the pump was tested by pumping liquid with 2,500 and 5,000 steps in both directions. The results are shown in Fig. S18 and should be considered when operating the pump without feedback.

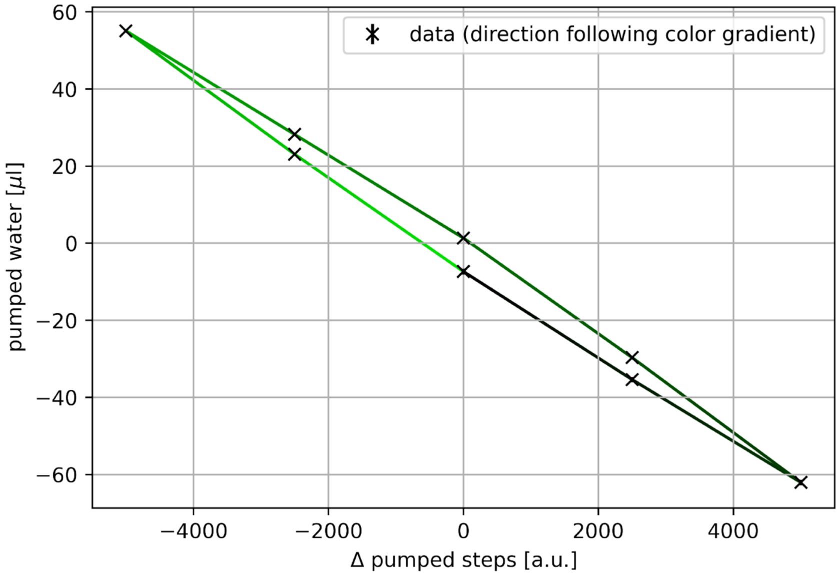

Figure S18 Hysteresis measurement on the pump, direction of pumping and measurement follows the color gradient from dark to light green (n=3). The measurement points are marked with an 'x'.

The minimum dry bath temperature was tested by connecting the Peltier elements to 9 V, while the reservoir was heated to 37 °C. The results are shown in Fig. S19.

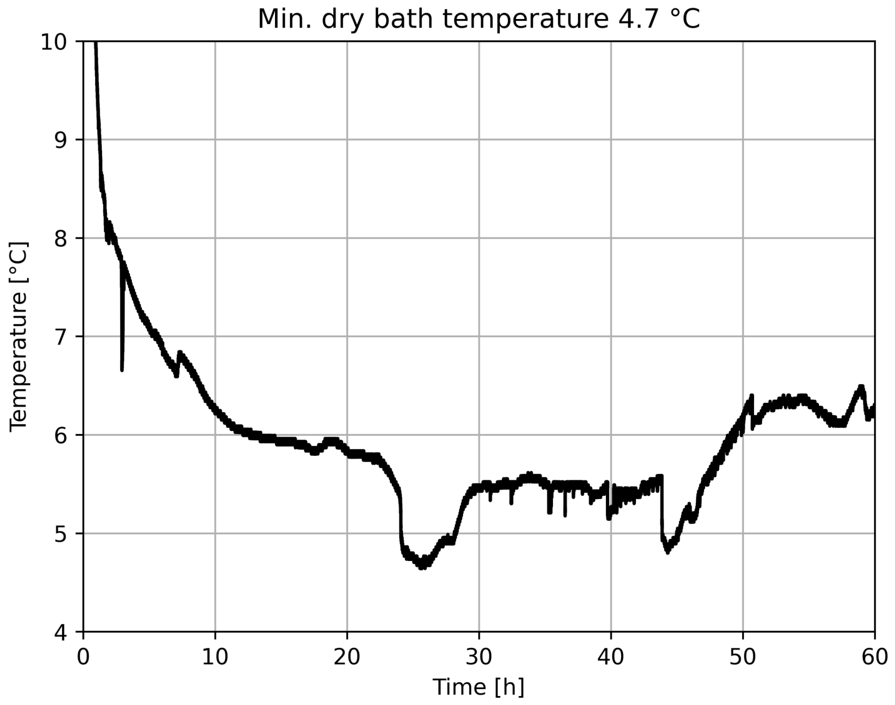

Figure S19 Measurement of the temperature of the liquid in the dry bath falcon tube measured by the liquid temperature sensor usually used for the MEAs.

B.3 Correlation: volume versus camera signal

Due to the cylindrical nature of the liquid reservoir, we assume that the liquid height is proportional to the medium volume. Furthermore, we present in Equation 1, that the reservoir height is proportional to the camera signal detected by inkulevel. In Fig. S20 we present a graphical argument for Equation 1.

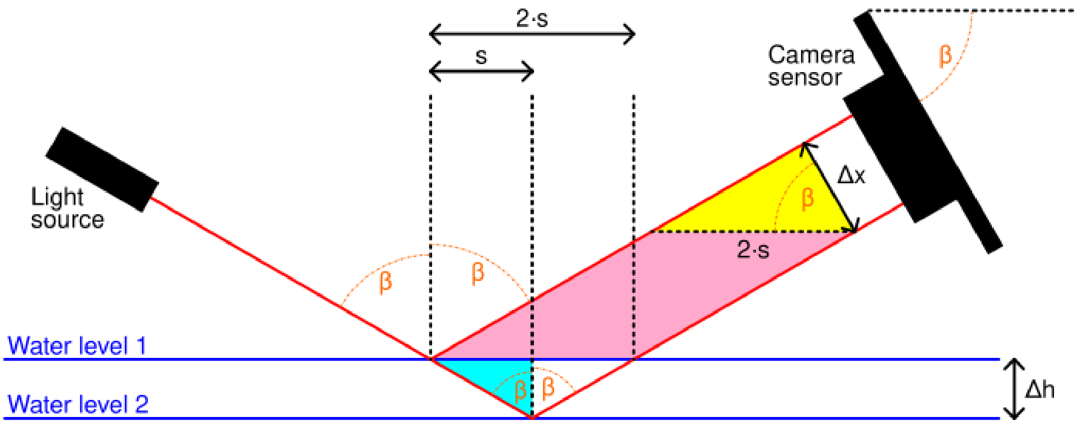

Figure S20 Graphical argument for Equation 1. The angle between the laser beam and the water surface normal is β. Due to the cyan triangle, we get that $s = \Delta h \cdot \tan\beta$. Furthermore, the hypotenuse of the yellow is $2 \cdot s$ due to the light red parallelogram. The yellow triangle gives us $\Delta x = 2 \cdot s \cdot \cos\beta$. By plugging in the value of s, we get $\Delta x = 2 \cdot \Delta h \cdot \sin\beta$ (Equation 1).

B.4 Spike shapes

Example spike shapes of a single electrode are provided in Fig. S21.

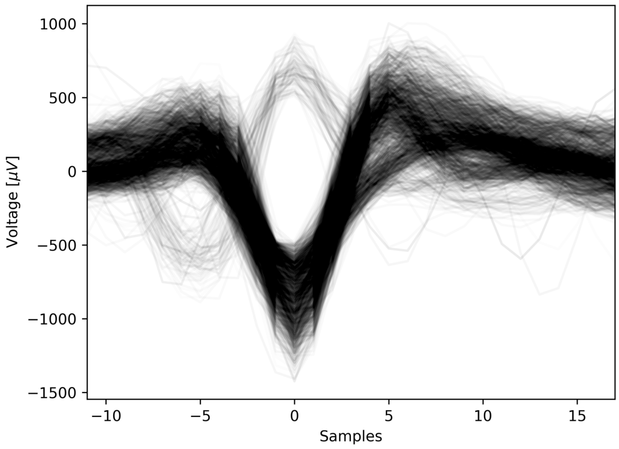

Figure S21 Example spike shapes of a single electrode, detected on the filtered signal with a threshold of 6. All shapes are aligned to the detected maximum.

B.5 Noise spectrum during perfusion

The power spectra of the filtered signal of an MEA with PBS are compared with a liquid path with PBS connected before and during active perfusion, shown in Fig. S22. RMS noise is unaffected by the liquid path but increased by a factor of roughly 2.5 during pumping, as can be seen in Fig. S23. The elevated noise level should be taken into account when measuring spikes during the pumping steps, as could be of interest during drug testing. As visible in the power spectra, increasing the order or cutoff frequency of the high-pass filter could resolve the issue, as pumping adds noise mostly below 1 kHz. Considering the required amounts to counteract evaporation, the pumping should not affect the results of long-term recordings.

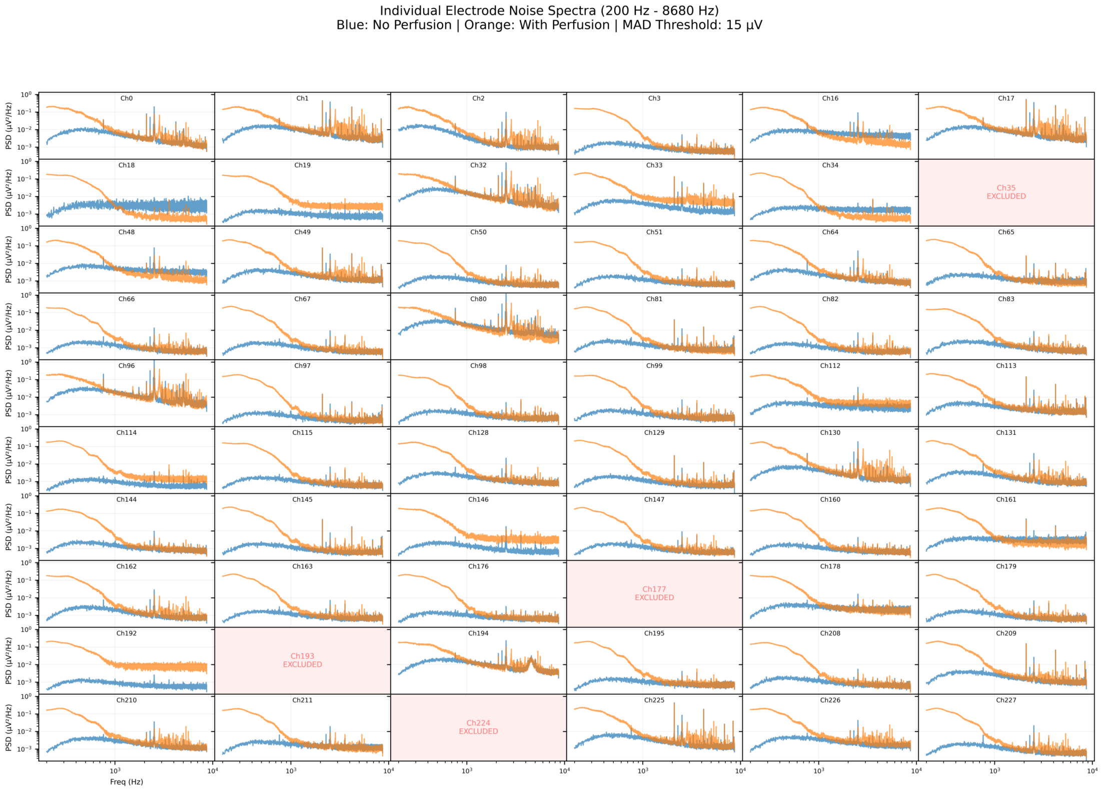

Figure S22 The power spectrum of a voltage recording of roughly 30 s is analyzed with and without perfusion. Perfusion is performed by adding an amount of 500 μL in 50 steps with breaks of 200 ms. Electrodes with an MAD of more than 15 μV were excluded, as this was assumed to be caused by a broken electrode or pogo pin.

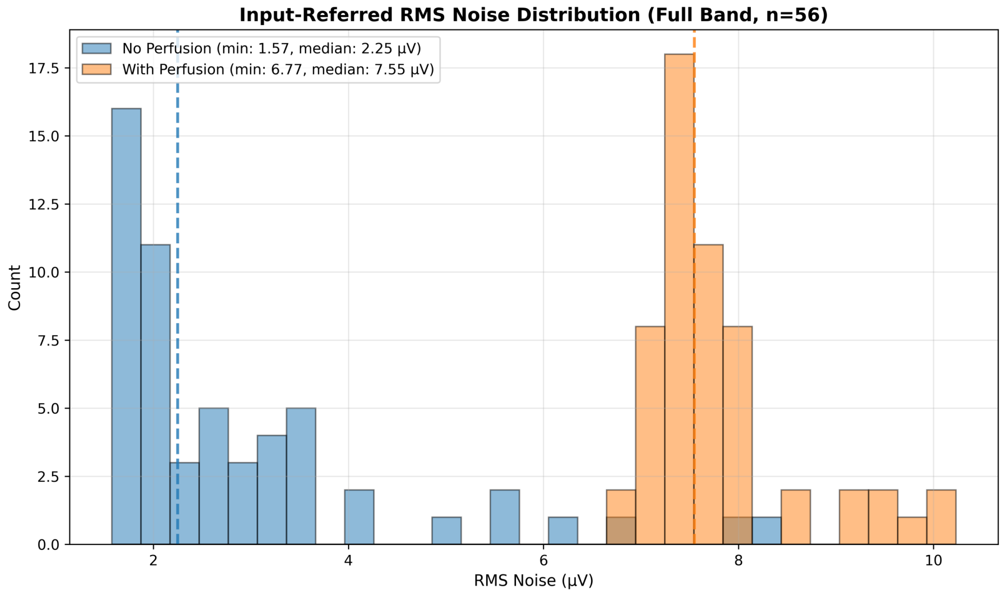

Figure S23 The RMS noise with and without perfusion.

B.6 Stimulation

An example trace of 16 evenly spaced pulses at 100 Hz is shown in Fig. S24. To reduce the impact of the stimulation artifact, an active discharge is performed on all electrodes of a stimulated chip. The discharge time is usually set between 0.5 and 1 ms.

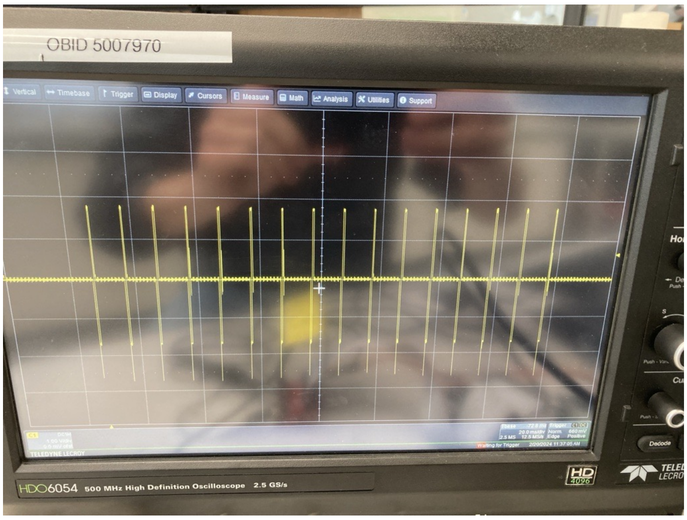

Figure S24 Stimulation pulses.

C Additional experimental data

Additional experimental data of the experiments shown in the main text are provided in this section.

C.1 Commercial system temperature characterization

The performance and stability of the temperature control of inkube is compared with a commercial system (MEA2100-Mini-60-System and MEA2100-System, Multi Channel Systems MCS GmbH, Reutlingen, Germany). The temperature is measured with the inkube PT1000 sensor inside the liquid. To create similar conditions to a mounted a inkube, the PT1000 is attached to a membrane cap reducing evaporation (ALA MEA-MEM, Multi Channel Systems MCS GmbH, Reutlingen, Germany). When the temperature control of the MEA2100 headstage is set to 37 °C, the liquid temperature settles at roughly 3.5 °C below at a ambient temperature of about 23 °C Fig. S25. The start-up is shown in Fig. S26 with an exponential fit.

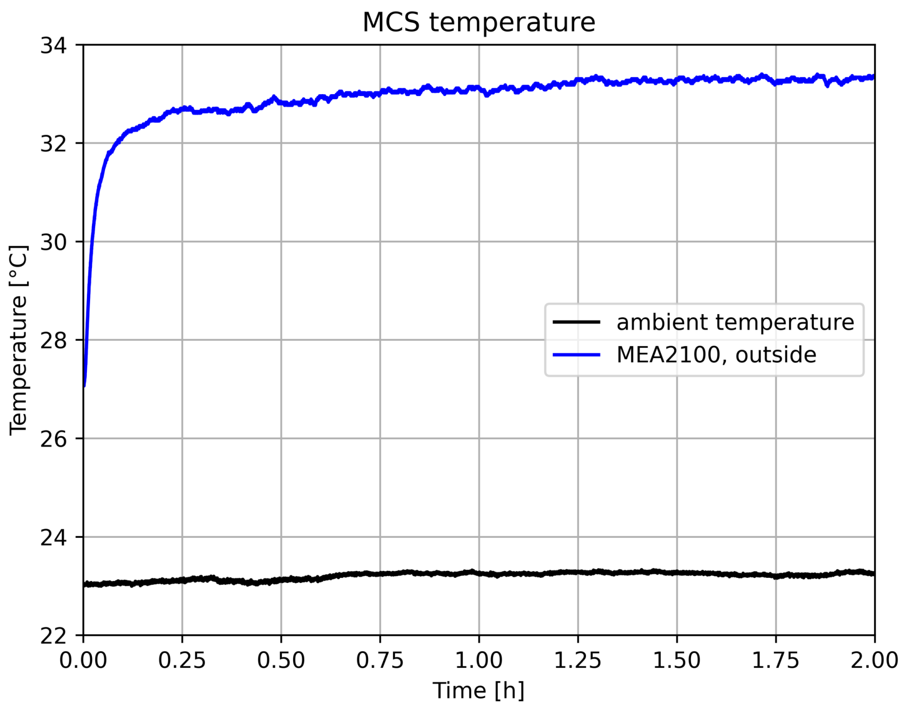

Figure S25 Ambient temperature and liquid temperature of a MEA inside the MEA2100 headstage, with the controller set to 37 °C.

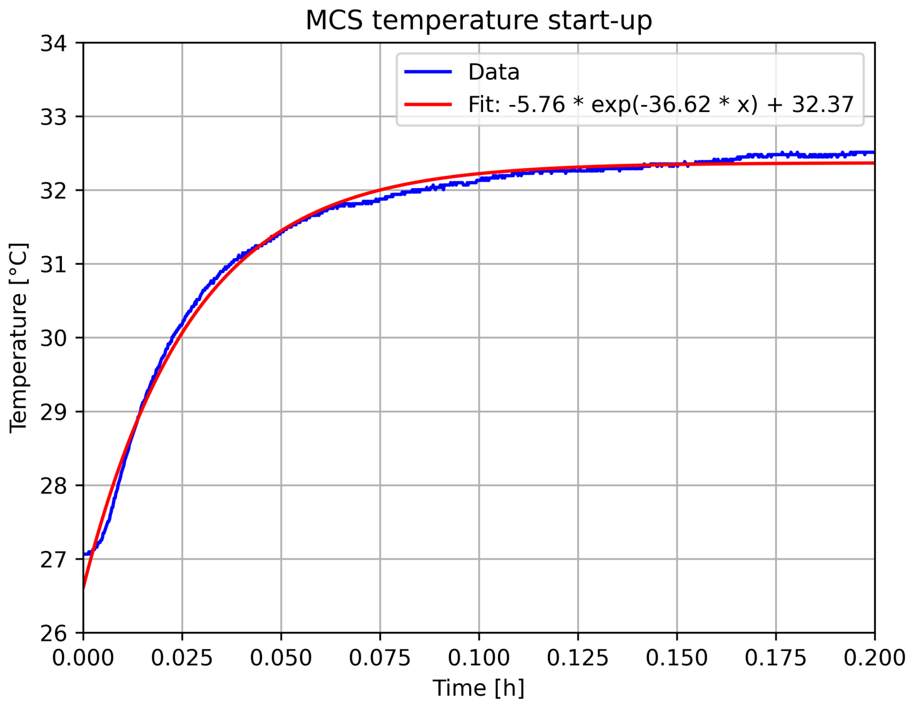

Figure S26 Exponential fit to the start-up of Fig. S25.

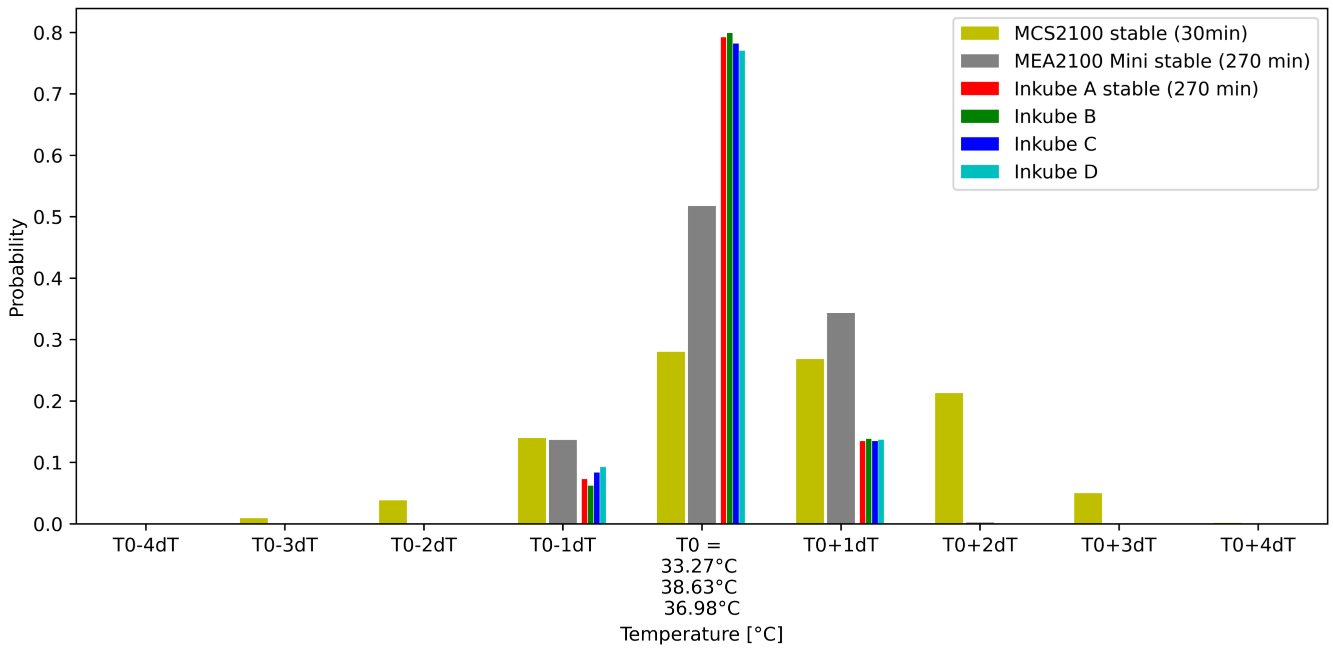

Figure S27 Temperature stability for 3 different devices. MCS2100 is the histogram of Fig. S25 between 90 and 120 min. MCS2100 Mini is the liquid temperature inside an MEA placed in a MEA2100 Mini with set temperature at 37 °C in an incubator (CB 170, BINDER Inc., Bohemia, NY, USA) with set temperature at 35 °C at steady state. The inkube MEAs are a replicate of Fig. 3A for direct comparison. The T0 for the 3 different devices in the x-axis label is listed in order of the histograms from left to right. dT corresponds to 31.7 mK.

C.2 Unordered data with temperature steps

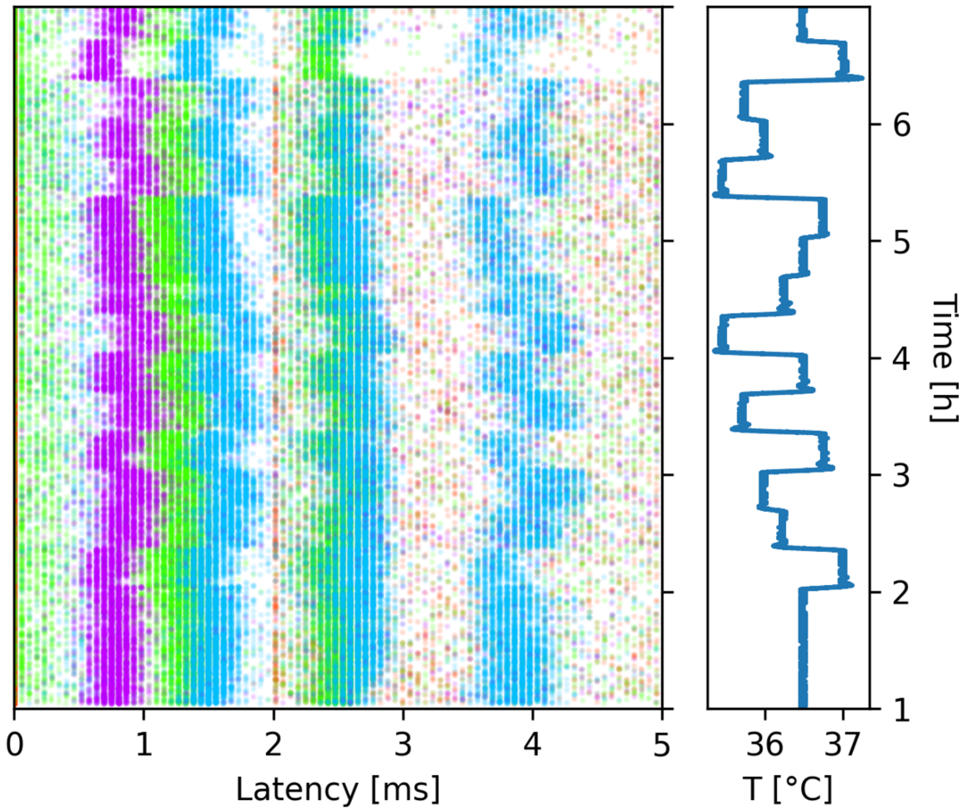

Figure S28 Unordered data from Fig. 4C.

C.3 Analysis for additional bands in Fig. 4C

.

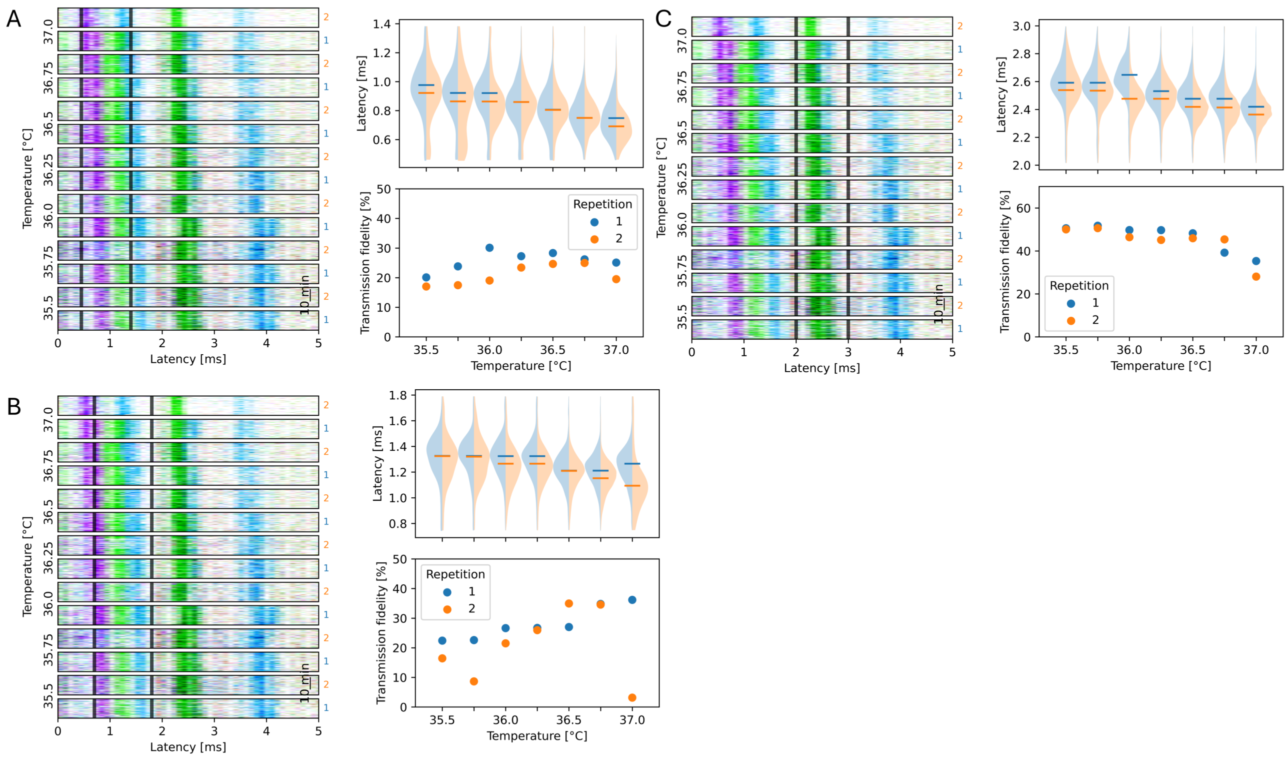

Figure S29 **(A)** Observation electrode 3 (starting at 0), band limits [0.45, 1.4] **(B)** Observation electrode 1 (starting at 0), band limits [0.7, 1.8] **(C)** Observation electrode 1 (starting at 0), band limits [2, 3]

C.4 Additional networks from the experiment shown in Fig. 5.

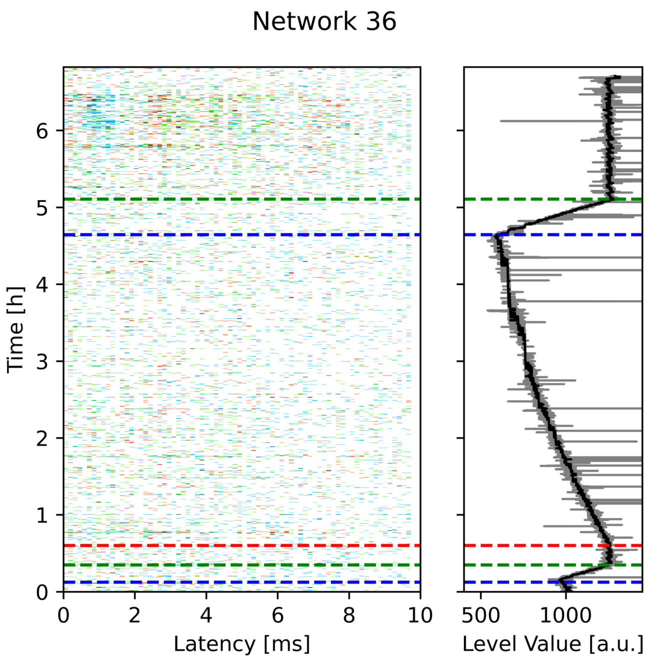

Figure S30 Network 7 on MEA C during evaporation.

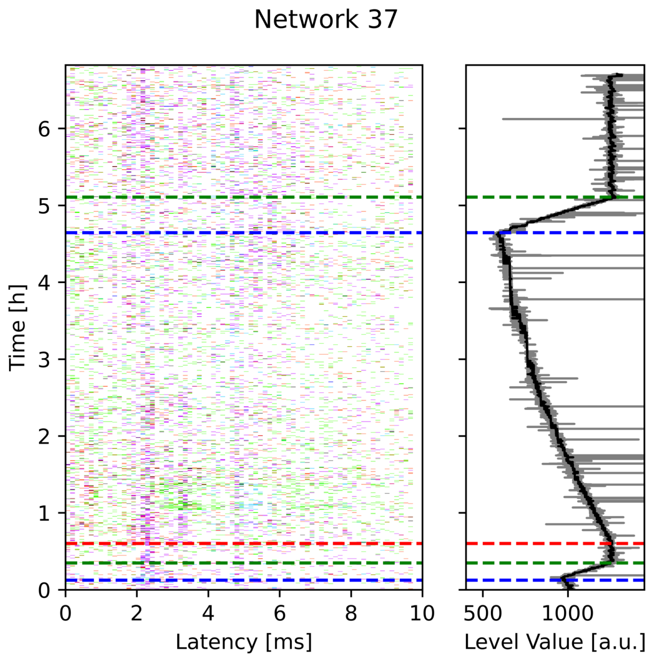

Figure S31 Network 8 on MEA C during evaporation.

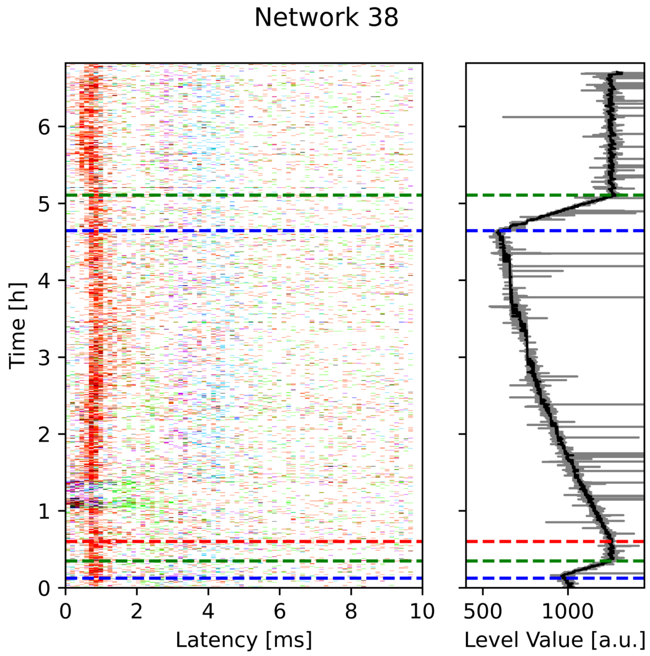

Figure S32 Network 9 on MEA C during evaporation.

C.5 Histogram of network responses and spontaneous recordings at different Magnesium concentrations

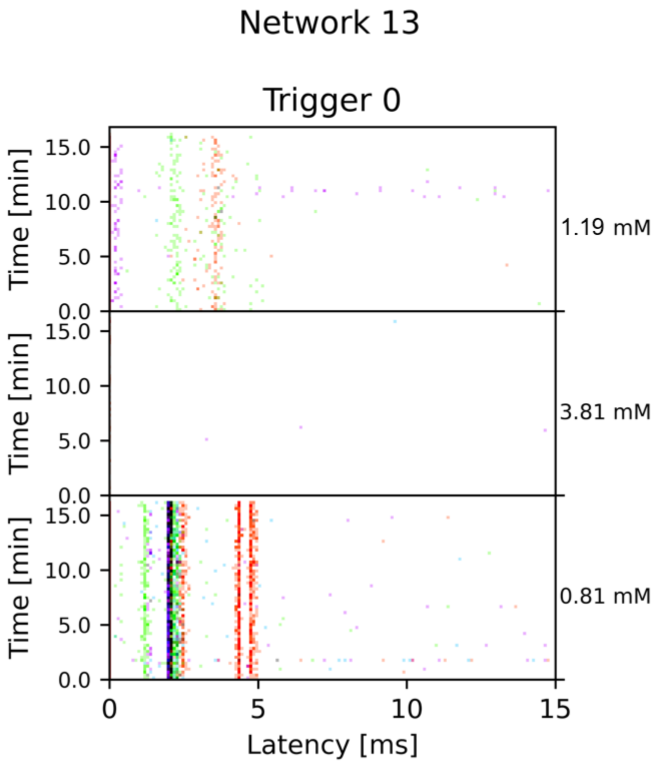

Figure S33 STTRPs of experiment in Fig. 6 at 0.81, 3.81 and 1.19 mM (second measurement after wash-out) before stimulus.

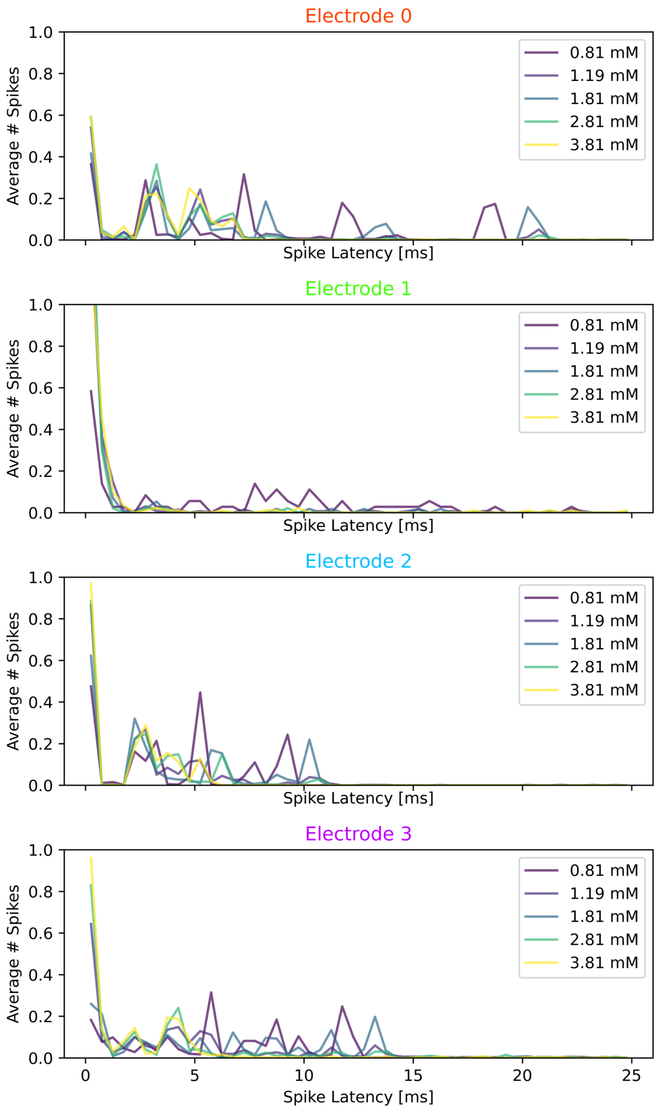

Figure S34 Spike count post stimulus with data from Fig. 6B binned to 0.5 ms.

C.6 Additional data from different networks at varying Magnesium concentration

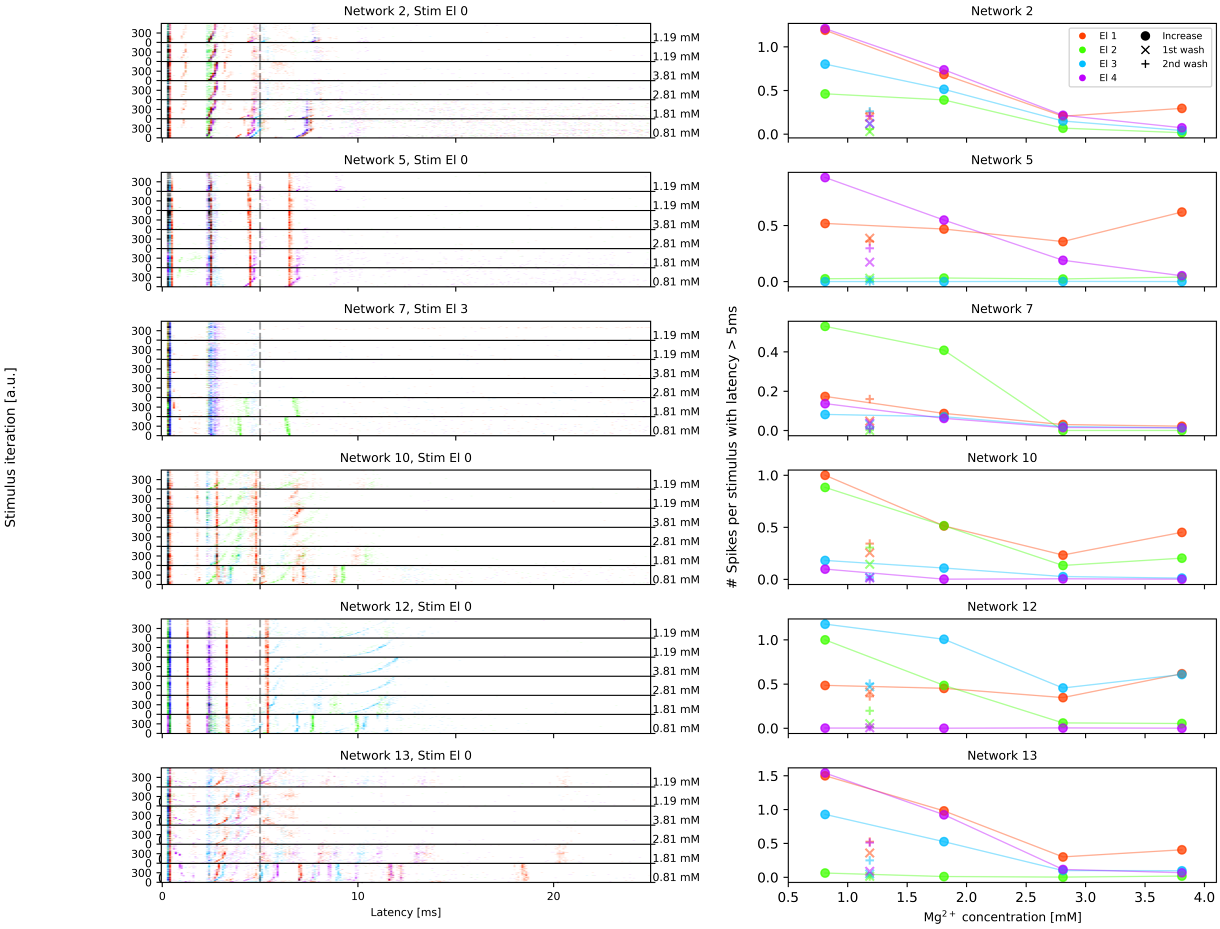

Figure S35 Data from 6 networks processed as described in Fig. 6.

C.7 Medium perfusion experiment

MEAs with iPSC-derived neurons were cultured in Neurobasal medium (NB) and BrainPhys Neuronal Medium (STEMCELL Technologies, Vancouver, BC, Canada) (BP). This experiment is showcasing the parallelization capabilities of inkube. Two MEAs of each condition are placed in the system and a half medium exchange is performed by removing 500 μL of culture medium and adding 500 μL of fresh medium, with a spontaneous recording of 10 min performed before and at least 15 min after. The mean firing frequency is determined per electrode and electrodes outside the range of 0.1 Hz and 50 Hz are excluded. The heatmaps before and after exchange on DIV 41 are presented in Fig. S36. Brainphys medium was supplemented with the same growth factors as the NBD medium described in the methods section. The 2 cultures were seeded into NBD using the aforementioned protocol and Brainphys medium was added by fully exchanging the medium on DIV 10. Afterwards, medium exchanges were performed by replacing half the medium every 3 to 4 days.

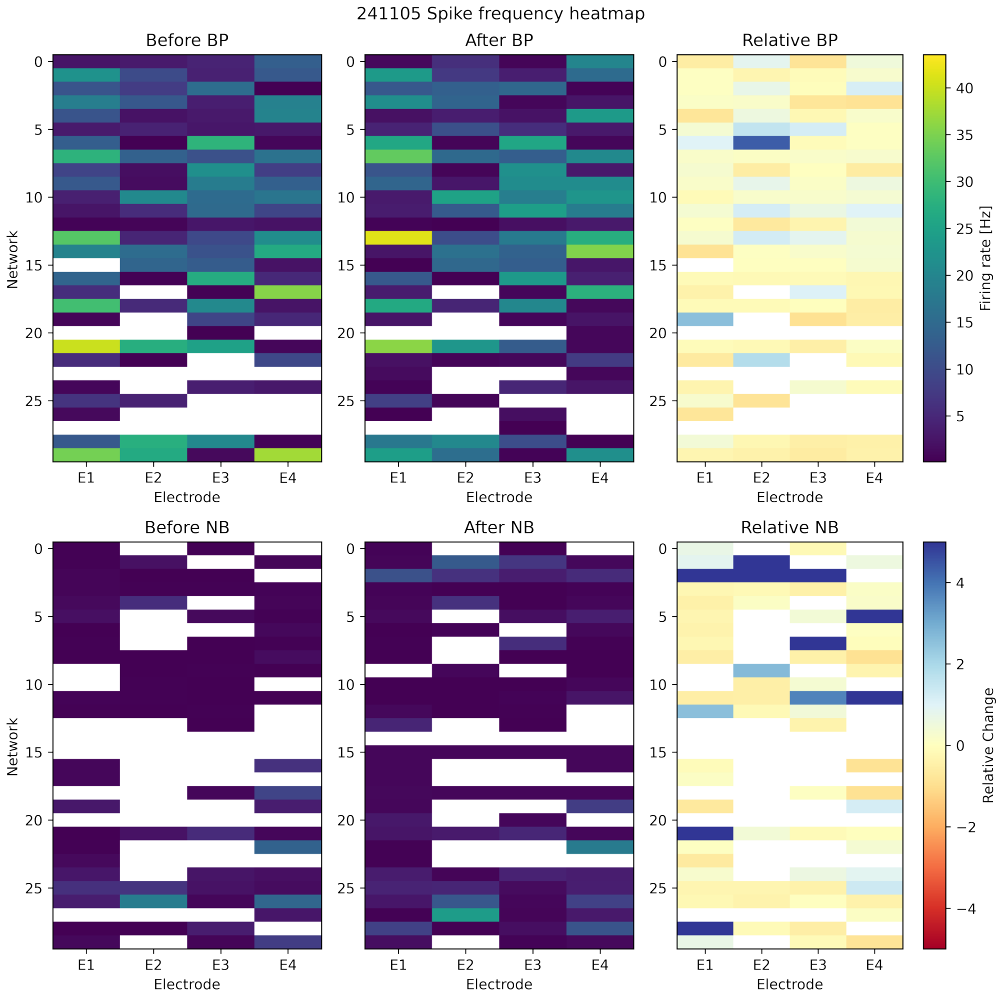

Figure S36 Frequency heatmap of all 240 electrodes before and after a medium exchange experiment. iPSC-derived neurons are recorded on DIV 41.

References

[1] Espressif Systems, 2023. Camerawebserver. https://github.com/espressif/arduino-esp32/tree/09a6770320b75c219053aa19d630afe1a7c61147/libraries/ESP32/examples/Camera/CameraWebServer. Accessed: 2024-11-13.
